# Stilbenes Against Alzheimer’s Disease: A Comprehensive Review of Preclinical Studies of Natural and Synthetic Compounds Combined with the Contributions of Developed Nanodrug Delivery Systems

**DOI:** 10.3390/molecules30091982

**Published:** 2025-04-29

**Authors:** Esra Küpeli Akkol, Gökçe Şeker Karatoprak, Berrak Dumlupınar, Özlem Bahadır Acıkara, Reyhan Arıcı, Çiğdem Yücel, Leyli Can Aynal, Eduardo Sobarzo Sánchez

**Affiliations:** 1 Department of Pharmacognosy, Faculty of Pharmacy, Gazi University, Ankara 06330, Türkiye; 2 Department of Pharmacognosy, Faculty of Pharmacy, Erciyes University, Kayseri 38039, Türkiye; gskaratoprak@erciyes.edu.tr; 3 Department of Nutrition and Dietetics, Faculty of Health Sciences, Istanbul Okan University, İstanbul 34959, Türkiye; berrak.dumlupinar@okan.edu.tr; 4 Department of Pharmacognosy, Faculty of Pharmacy, Ankara University, Ankara 06560, Türkiye; obahadir@ankara.edu.tr; 5 Department of Pharmacognosy, Faculty of Pharmacy, Ankara Medipol University, Ankara 06570, Türkiye; reyhan.arici@ankaramedipol.edu.tr; 6 Department of Pharmaceutical Technology, Faculty of Pharmacy, Erciyes University, Kayseri 38039, Türkiye; cyucel@erciyes.edu.tr; 7 Etlik City Hospital, Department of Neurology, Ankara 06170, Türkiye; leylican.aynal@saglik.gov.tr; 8 Centro de Investigación en Ingeniería de Materiales, Facultad de Medicina y Ciencias de la Salud, Universidad Central de Chile, Santiago 8330507, Chile; 9 Department of Organic Chemistry, Faculty of Pharmacy, University of Santiago de Compostela, 15782 Santiago de Compostela, Spain

**Keywords:** stilbene, Alzheimer’s disease, natural compound, structure–activity relationships, nanoformulation

## Abstract

This review covers preclinical studies of stilbene derivative compounds (both natural and synthetic) with potential preventive and therapeutic effects against Alzheimer’s disease (AD). AD is a worldwide neurodegenerative disease characterized by the destruction of nerve cells in the brain and the loss of cognitive function due to aging. Stilbenes are a unique class of natural phenolic compounds distinguished by a C6-C2-C6 (1,2-diphenylethylene) structure and two aromatic rings connected by an ethylene bridge. Stilbenes’ distinct features make them an intriguing subject for pharmacological research and development. Several preclinical studies have suggested that stilbenes may have neuroprotective effects by reducing Aβ generation and oligomerization, enhancing Aβ clearance, and regulating tau neuropathology through the prevention of aberrant tau phosphorylation and aggregation, as well as scavenging reactive oxygen species. Synthetic stilbene derivatives also target multiple pathways involved in neuroprotection and have demonstrated promising biological activity in vitro. However, some properties of stilbenes, such as sensitivity to physiological conditions, low solubility, poor permeability, instability, and low bioavailability, limit their usefulness in clinical applications. To address this issue, current investigations have developed new drug delivery systems based on stilbene derivative molecules. This review aims to shed light on the development of next-generation treatment strategies by examining in detail the role of stilbenes in Alzheimer’s pathophysiology and their therapeutic potential.

## 1. Introduction

Alzheimer’s disease (AD) is a neurological disorder that primarily affects older persons and is distinguished by memory loss, behavioral decline, performance impairment, and slow thinking. The buildup of oxidative damage to the brain’s proteins, mitochondria, and nucleic acids causes cognitive and neurological dysfunction [1]. Neurofibrillary tangles (NFTs) and senile plaques (SPs) are thought to be the two main pathology signs of AD. Amyloid β (Aβ) accumulation in the brain, oxidative stress, environmental factors, genetics, inflammation of neurons, and mitochondrial dysfunction are some of the various processes of neurodegeneration [2]. Current treatment options for AD patients are supportive, improving memory and alertness, and slowing down the overall progression of the disease. Preventing cognitive decline and maintaining a healthy lifestyle have shown the importance of diet and exercise in this disease. Although there are some FDA-approved drugs prescribed for AD, it is known that the results are often unsatisfactory and there is still a need for alternative molecules, especially those obtained from plants [3].

The field of pharmaceutical research is increasingly focusing on natural compounds, particularly those derived from plants, due to their potential to treat diseases with minimal side effects. One such group of natural compounds is stilbenes. Due to their intense absorption and fluorescence properties, they are named after the Greek word “stilbos”, which means “shining”. They are also recognized as phytoalexins and are found in several plant families. They are synthesized by plants as defense mechanism products serving as defensive substances in response to various stress factors such as pathogenic attacks, bacterial and fungal growth, and the damaging effects of UV light [4,5,6]. Stilbenes also play essential roles in the interactions between plants and symbiotic bacteria. Certain bacteria, including Bacillus and Photorhabdus, can synthesize specific stilbene derivatives [4,6].

Stilbenes are a distinctive group of natural phenolic compounds not found in all plant families because the critical enzyme responsible for their production, stilbene synthase (STS), is not universally present. They are also present in limited amounts in the human diet, primarily in foods such as grapes, red wine, peanuts, and berries. Biochemically, stilbenes encompass a diverse group of compounds derived from the phenylpropanoid pathway, including basic stilbenes, dihydrostilbenes, (bis) bibenzyls, phenanthrenes, and related substances. These specialized metabolites are characterized by a C6–C2–C6 (1,2-diphenylethylene) structure and defined by two aromatic rings linked by an ethylene bridge. These unique properties of stilbenes make them a fascinating area of study in pharmaceutical research and development. The intricate chemical compositions and diverse biological properties of stilbenes also contribute significantly to their prominence within the phenolic compound category. For example, resveratrol, the most well-known stilbene, and combretastatin A-4 demonstrate therapeutic and preventive capabilities [6,7,8]. Resveratrol, known for its anti-inflammatory effects and association with the “French paradox”, is the primary reason for the increasing interest in these compounds [5,9]. This compound has garnered extensive scientific attention over the past two decades and exhibits a broad range of biological activities. Studies have revealed its ability to influence cell proliferation, angiogenesis, redox status, mitochondrial activity, adipocyte lipolysis, and the suppression of inflammation [6,10]. Recent research has also delved into other stilbenes, particularly monomer and dimer compounds. Stilbene derivatives exhibit a wide range of structural diversity, from individual units to octamers. They can encompass a variety of substituents, such as glycosyl, hydroxyl, methyl, or isopropyl groups positioned at different locations [11]. The modifications to resveratrol can result in stilbenes with a broad spectrum of properties, allowing for potential interactions with the immune system. The placement of hydroxyl groups within the stilbene structure is crucial in dictating anti-inflammatory characteristics and cellular permeability. Stilbene derivatives possess antioxidant properties and can modulate inflammation markers, suggesting potential protection against chronic diseases [5,6,7,12]. Numerous studies have also demonstrated that stilbenes can have neuroprotective effects on the molecular mechanisms that cause AD. They can act as antioxidants or anti-neuroinflammatory drugs, influencing the reduction in the neurotransmitter ACh in cholinergic neurons, the hyperphosphorylation of tau protein, and the aggregation of β amyloid peptides [13,14,15,16,17].

Many comprehensive reviews are focusing on resveratrol, but there is a gap in the current literature with regards to studies examining the bioactivity of other stilbenes, synthetic stilbenes, and their nanoformulations concerning AD. Therefore, this review aims to shed light on the development of new-generation treatment strategies by examining the chemistry of stilbenes, their synthesis pathways, the results of natural stilbenes and synthetic stilbenes in preclinical studies related to AD, and also the possible advantages of their nanoformulations from a broad perspective.

## 2. Natural Sources for Stilbene Derivatives

**Figure 1:**
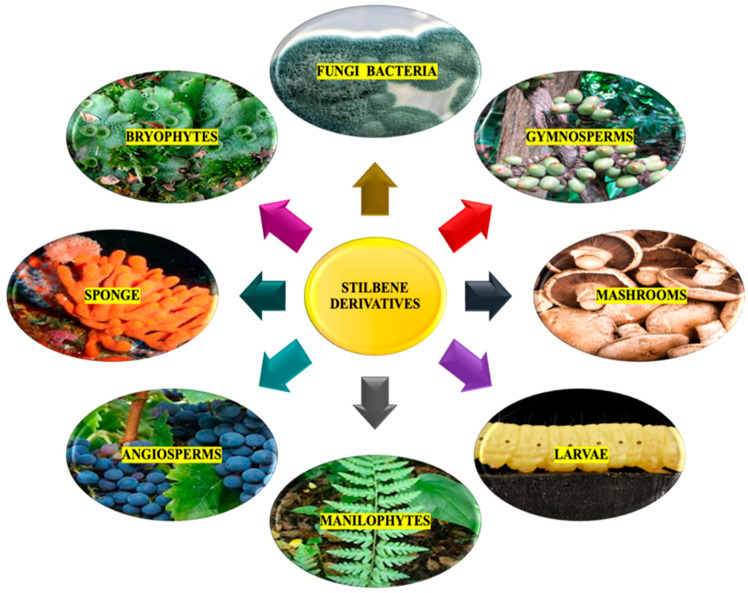
Natural sources for stilbene derivatives.

Advancements in analytical and spectroscopic techniques have greatly improved understanding of the complex structures of stilbenes despite their limited presence in the plant kingdom; especially significant progress has been made in elucidating the structures of many oligomeric stilbenes. A comprehensive analysis has revealed that more than 1000 distinct stilbenes have been isolated from various plant species belonging to 275 different species from 105 genera and 55 family sources [18]. While extensive reviews have been on resveratrol and its analogs, focusing on their pharmacological mechanisms and clinical applications, the exploration of stilbenes in phytochemical investigations has mainly centered on a select few families. Stilbenes are natural compounds found widely in various natural sources. They are present in Bryophytes, particularly in the Marchantiales order, Monilophytes including the Ophioglossales and Polypodiales orders, and in gymnosperms, especially the Gnetales and Pinales orders. Several species of bryophytes have been used to isolate many methoxylated stilbenoids. Additionally, stilbene derivatives can be found in angiosperms, both in Monocots and Eudicots. The presence of stilbenes is notable in several plant families, such as Dipterocarpaceae, Moraceae, Gnetaceae, Orchidaceae, Polygonaceae, Euphorbiaceae, Stemonaceae, Cyperaceae, Pinaceae, Fabaceae, Vitaceae, and Asparagaceae. These families represent diverse botanical lineages, indicating the widespread occurrence of stilbenes in various plant families, accounting for 84% of the total stilbene content. The families Dipterocarpaceae and Gnetaceae are abundant sources of natural oligomeric stilbenes, representing more than 50% of the total reported natural oligostilbenes. In contrast, the family Leguminosae contained the highest number of new monomeric stilbenes [7,8,11,18]. Over a few oligomers, monomers, and unique stilbene hybrids have been isolated from numerous Gnetum species [7,19,20]. Moreover, the genus *Welwitschia* [21] and *Picea* species have been found to contain stilbene derivatives mainly [6,8,22,23]. In numerous research studies, stilbenes, including t-resveratrol and other stilbenoids, have also been identified in a diverse range of sources. These sources include plant endophytic fungi, entomopathogenic bacteria, various edible mushrooms, sponges, and even moth larvae (Figure 1) [24,25,26,27,28,29].

Resveratrol, a well-known stilbene derivative, has been extracted from a variety of sources, including peanuts, grapes, bilberries, and cranberries, among others, comprising 100 species from 34 different families of medicinal plants, foods, and other natural sources [30]. In particular, *Vitis* in the Vitaceae family is the primary source of dietary stilbenes, particularly E-resveratrol in grapes and red wine [31]. Stilbenes are mainly found in grapes and wine, but their levels can vary. Factors like grape variety, soil type, temperature, and pathogen attacks influence their production. As natural plant defenses, stilbene levels change based on conditions. Additionally, processing grapes into juices or wines can affect stilbene amounts due to postharvest and winemaking practices [6,12,32]. The glucosylated form (piceid) of resveratrol can also be found in several varieties of *Vitis* species and vines, the amount of which can vary. Resveratrol, piceid, piceatannol, astringin and pterostilbene as the other types of stilbenes, as well as dimeric types of resveratrol such as pallidol, ε-viniferin, and δ-viniferin resveratrol–resveratrol and piceatannol–piceatannol homodimers together with their heterodimers and modified types of dimers such as O-glycosylated, methoxylated and oxidized, multiple trimers and tetramers have also been detected in varying amounts, which indicated the chemical complexity of stilbenes in grapes and wines [12,33,34,35].

## 3. Chemistry and Structural Classification of Stilbenes

**Figure 2:**
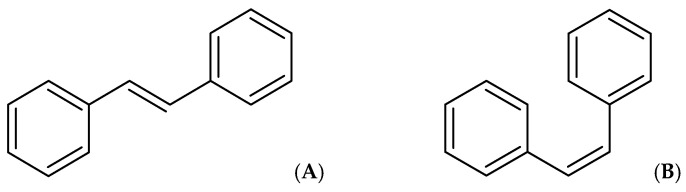
(*E*)-stilbene (trans-stilbene) (**A**) and (*Z*)-stilbene (cis-stilbene) (**B**).

Stilbenoids belong to a group of natural phenolic compounds known for their distinct stilbene backbone structure, which consists of two benzene rings connected by an ethylene segment. These compounds exist naturally in two isomeric forms: (E)-stilbene (trans-stilbene), which is not hindered by steric interactions, and (Z)-stilbene (cis-stilbene) (Figure 2), which is less stable due to steric hindrance between the aromatic rings [36].

Based on their chemical structure, stilbenoids can be classified into four major groups:

Group 1: Simple stilbenes
Group 2: Prenylated and geranylated stilbenes
Group 3: 2-phenyl-benzofuran derivatives
Group 4: Carbon-substituted stilbenes (excluding prenylated and geranylated stilbenes) which have acyl, benzyl, and carboxyl groups [37].

Stilbenes can also be categorized based on their molecular structure. These categories include monomeric stilbenes (individual units), oligomeric stilbenes including dimeric (formed by the combination of two units), trimeric (formed by the combination of three units), tetrameric (formed by the combination of four units), as well as a further combination of stilbenes monomers and polymeric stilbenes that composed of multiple units in a polymerized form and heteromeric stilbenes which are formed by the combination of different molecular skeletons [38,39].

### 3.1. Simple Stilbenes

**Figure 3:**
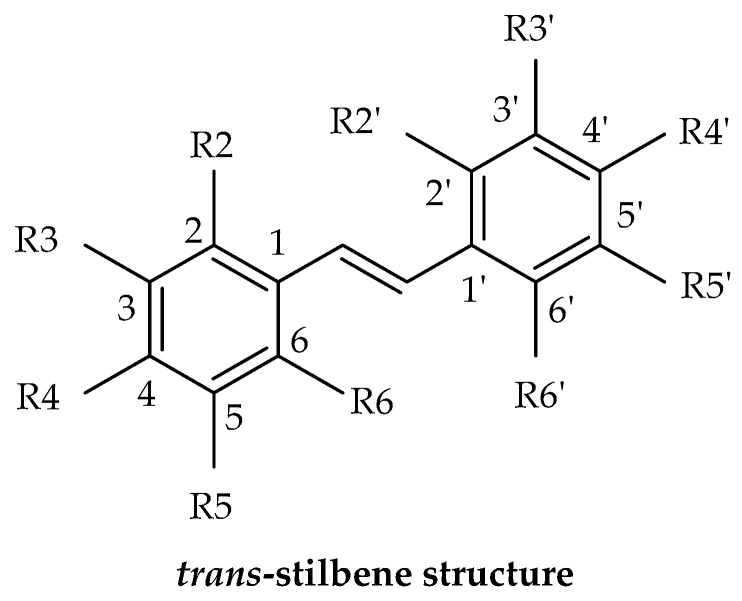

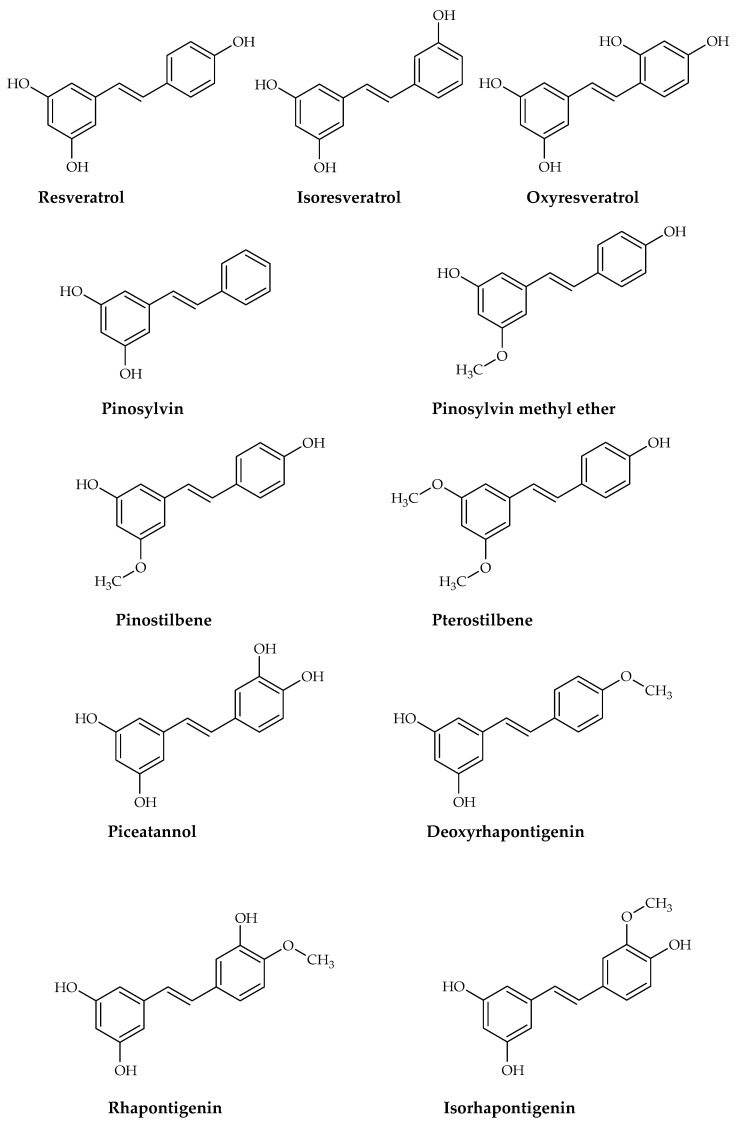
The main structure of stilbene derivatives and some simple stilbene derivatives isolated from natural sources.

Simple stilbenes are described as the first category. It is important to note that these derivatives show various substitution patterns on the aromatic rings, which results in different types of stilbenes and significantly impacts their chemical properties and biological activities. Most stilbenes in this category are 3,5-dihydroxy substituted, and the second aromatic ring may have hydroxyl or methoxy groups. Resveratrol is one of the best-known examples of stilbene derivatives and is widely found in natural sources. Resveratrol was first isolated from *Veratrum grandiflorum* in 1940 [40]. Since then, the chemical synthesis of resveratrol has been extensively documented [41,42]. Although the yields from chemical synthesis are relatively high, the process is complex, and contamination is a significant issue [43]. The examples in this group are displayed in Figure 3.

### 3.2. Prenylated and Geranylated Stilbenes

**Figure 4:**
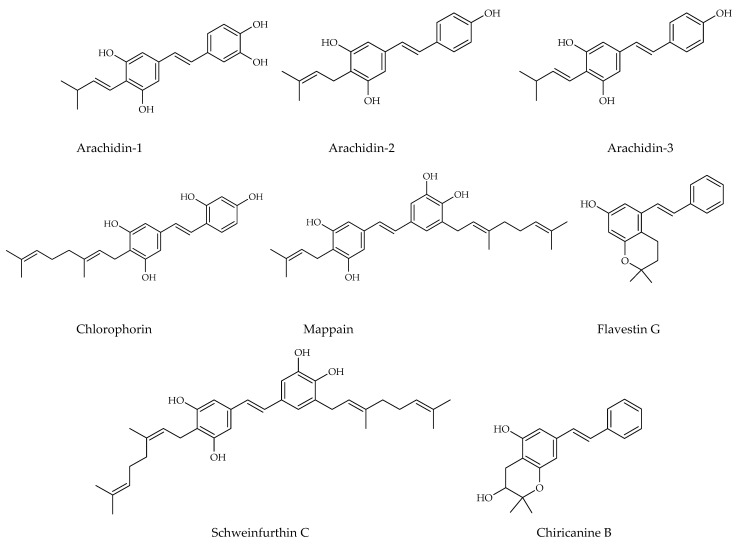
Prenylated stilbene structures isolated from natural sources.

Prenylated and geranylated stilbene derivatives have additional carbon-carbon bonds in the main core, featuring terpenic substituents like prenyl, geranyl, and hydroxyprenyl groups. These groups, derived from isoprenoid precursors, typically consist of five and ten carbon atoms, respectively, and are incorporated through enzymatic reactions in plants, fungi, and microorganisms. The most suitable positions for terpenic substitution are 2, 4, 6, and/or 5′. Modifications such as cyclization and hydroxylation can also occur, leading to unique compounds with enhanced lipophilicity and bioavailability. These type of stilbenoids are predominantly found in certain plant families like Moraceae and Leguminosae, with over 400 derivatives identified from natural sources as shown in Figure 4 [31,39,44,45].

Arachidin-1, arachidin-2, arachidin-3, arachidin-5, arahypin 4-7, 13-15, and chiricanine A were isolated from *Arachis hypogea* [46,47,48,49]. Prenylated stilbenoids have also been reported from *Artocarpus* species. 3-(,3-Dihydroxy-3-methylbutyl) resveratrol, 3-dimethylallyl-resveratrol and 5-dimethylallyl-oxyresveratrol from *Artocarpus dadah*, artocarbene from *Artocarpus incisus* [50,51], (*E*)-4-isopentenyl-3,5,2′,4′-tetrahydroxystilbene and (*E*)-4-(3-methyl-*E*-but-1-enyl)-3,5,2′,4′-tetrahydroxystilbene from *Artocarpus nobilis* [52], and 7-[(1*E*)-2-(4-hydroxyphenyl)ethenyl]-2,2-dimethyl-2H-1-benzopyran-5-ol from *Artocarpus altilis* [53] have been reported. *Lonchocarpus chiricanus* from Leguminosae family contains prenylated stilbene derivatives named chiricanines A–E together with longistylines C and D [54,55,56]. Mappain is a cytotoxic stilbene found in *Macaranga mappa* which is a derivative of piceatannol that is substituted with a prenyl and a geranyl group [57]. Schweinfurthins A, B, and D are structurally similar hexahydroxanthene stilbenes isolated from *Macaranga schweinfurthii* [58,59].

### 3.3. 2-Phenyl-Benzofuran Derivatives

**Figure 5:**
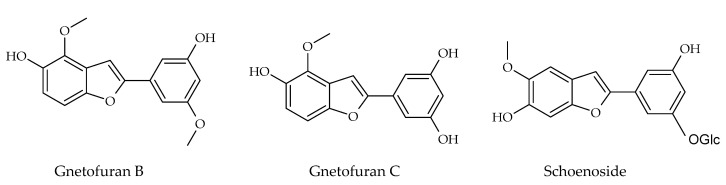

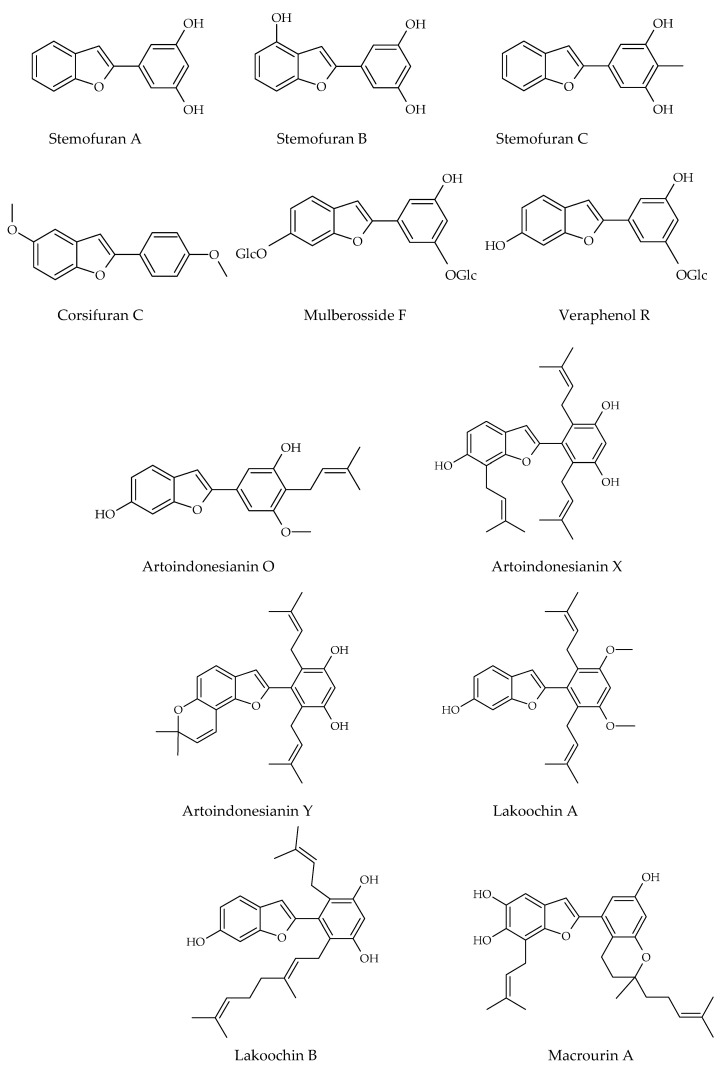
Prenylated 2-arylbenzofuran stilbene structures isolated from natural sources.

Besides these above-mentioned two groups, other types of stilbenes are found in nature. These include 2-phenyl-benzofuran derivatives, naturally occurring compounds comprising a benzofuran nucleus (rings A–C) and a phenyl unit (ring B) substituted at carbon 2. Arylbenzofuran derivatives are a group of special stilbenes formed by C_7_–O–C_7_ linkage, and they are naturally occurring compounds with various pharmacological activities. The 2-aryl benzofurans are found in only a few numbers of known families: Corsiniaceae, Gnetaceae, Melanthiaceae, Stemonaceae, Moraceae, Fabaceae, and Vitaceae [7,8,39]. For example, artopithecins A–D are prenylated derivatives from *Artocarpus pithecogallus.* Lakoochin A has two prenyl groups, while lakoochin B has a prenyl substituent at C-2 and a geranyl substituent at C-6 from *Artocarpus lakoocha*. Artoindonesianin derivatives also have been isolated from *Artocarpus gomezianus*. Regiafurans A–C have been isolated from *Chlorophora regia* and a series stemofurans A–K (1–11) from the *Stemona collinsiae* roots [60,61,62]. Gnetifolins and gnetofurans have been isolated from *Gnetum* species, while bulbophyllin and densiflorol A have been isolated from *Bulbophyllum protractum* and *Dendrobium densiflorum*, respectively [37,63]. Corsifuran C has been isolated from the liverwort *Corsinia coriandrina*. Schoenoside, as a 2-arylbenzofuran glucoside, has been isolated from the rhizomes of *Schoenocaulon officinale* (Liliaceae), veraphenol and mulberroside F, of which the last is a diglucoside and is obtained from *Veratrum taliense* and *Morus alba,* respectively. Another benzofuran derivative, macrourin A, from the barks of *Morus macroura*, carries both prenyl and geranyl moieties. Figure 5 displays some examples of 2-aryl benzofuran and prenyl-substituted 2-arylbenzofuran stilbene structures from natural sources [64].

### 3.4. Carbon-Substituted Stilbenes

**Figure 6:**
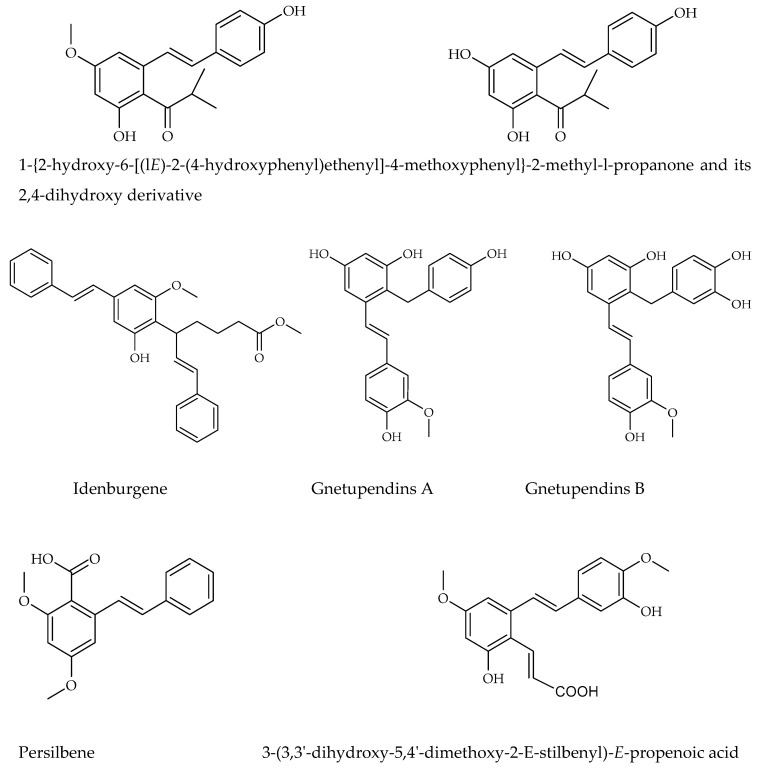

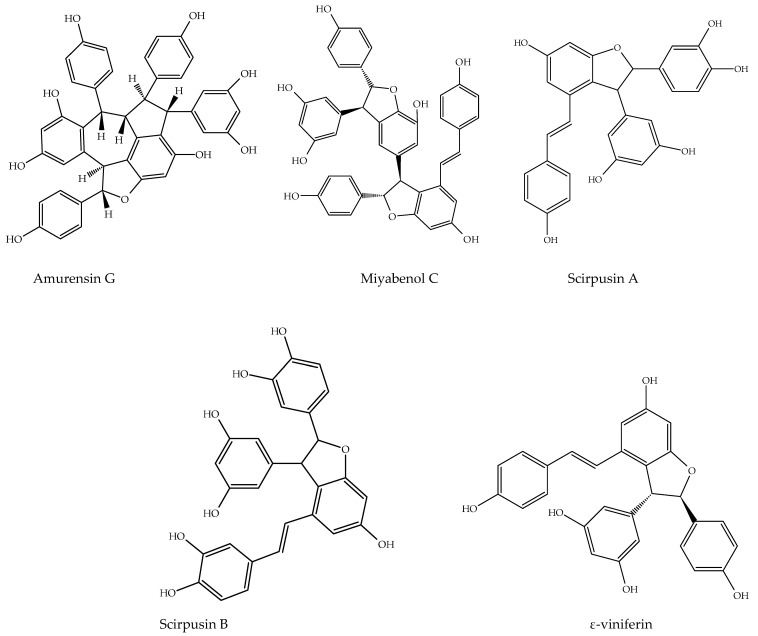
Other stilbene structures isolated from natural sources.

The other stilbene derivatives (Figure 6), as the fourth group, involve the derivatives which could not have been classified under the first three classes. Carbon-substituted stilbenes which do not contain prenyl and/or geranyl substitution are also categorized under this class. The compounds may involve acyl, benzyl, and carboxyl substitutions. There are various other stilbene structures, which these compounds could not be classified due to their distinctive chemical cycle [37]. Isobutanoyl derivatives which are described as 1-{2-hydroxy-6-[(l*E*)-2-(4-hydroxyphenyl) ethenyl]-4-methoxyphenyl}-2methyl-l-propanone and its 2,4-dihydroxy derivative have been isolated from *Ekebergia benguelensis*. Idenburgene isolated from *Cryptocarya idenburgensis* which has a unique structure, gnetupendins A and B from the *Gnetum pendulum* with benzyl substituents, 2-carboxy stilbene which named as persilbene from *Polygonum persicaria* as well as 3-(3,3′-dihydroxy-5,4′-dimethoxy-2-E-stilbenyl)-*E*-propenoic acid from *Convolvulus hystrix* and 5-O-terpinen-4-yl derivative purified from *Alpinia katsumadai* could be categorized under this class [37,63,65].

Stilbenes can be classified by alternative classification method which is based on the number of stilbenes structures they contain. This method is potentially simpler than the previous categorization system. Monomeric stilbenes have a basic structure based on the stereochemistry of the C-C double bond; they can exist in a cis- or trans-configuration. The trans-stilbene isomer (*E*) is more stable, constituting 89% of the total. Oligomeric stilbenes consist of a combination of different numbers of stilbene structures, ranging from two to ten. These types are produced by coupling homogeneous or heterogeneous monomers of stilbene. All stilbene may exist as free phenolic derivatives (aglycone) or conjugated as glucosides. One of the most well-known types is dimeric stilbenes. Over 240 dimeric stilbene components have been identified in recent years, predominantly isolated from plant families, including Dipterocarpaceae, Polygonaceae, Vitaceae, Leguminosae, and Gnetaceae. These discoveries emphasize the diverse sources of dimeric stilbenes and their potential pharmacological importance. Trimeric stilbenes and tetrameric stilbenes from Dipterocarpaceae, Leguminosae, and Vitaceae have also been isolated from natural resources. Polymeric stilbenes are a type of natural stilbene derivatives referred to as phenolic compounds. They are derived from plants and over twenty polymeric stilbenes have been identified. These include stilbene pentamers, stilbene hexamers, stilbene heptamers, and stilbene octamers. These compounds have been found exclusively in the Dipterocarpaceae and Vitaceae families. Heteromeric stilbenes [7,11,63].

Various patterns mentioned can be utilized during the production of oligomeric stilbenes, resulting in structures with intricate skeletons, complex configurations, and varying levels of oligomerization due to the different possible units. Based on the biosynthetic pathway of the oligomeric stilbenes, the construction patterns are divided into four major groups based on the number of connecting bonds between two monomeric stilbene units:

- Two individual units linked by only one C-C or C-O-C bond (with two connection points)
- Two individual units connected by two C-C or C-O-C bonds (with four connection points), often forming a ring. For instance, the distinct dihydrobenzofuran segment is typically formed by two units with a C-C and a C-O-C bond.
- Two individual units connected by three C-C or C-O-C bonds (with six connection points) create two rings.
- Two individual units connected by four C-C or C-O-C bonds (with eight connection points). This configuration is rare [7].

## 4. Structural Characteristics of Stilbene Derivatives

Stilbenes are a group of fascinating compounds found in plants, existing in two natural isomeric forms: trans-stilbenes (*E*-stilbenes) and cis-stilbenes (*Z*-stilbenes). Trans-stilbenes are particularly captivating due to their well-documented biological activities, while cis-stilbenes are formed as a degradation product following the photooxidation of trans-stilbenes. It is worth noting that stilbenes, particularly trans-stilbenes, are highly sensitive to light. Exposure to light causes trans-stilbenes to change into the less bioactive cis-isomer, a degradation product of the molecule. For example, in the case of resveratrol, this transformation occurs after several hours of exposure to sunlight or when the molecule is exposed to ultraviolet light. The degradation of cis-resveratrol can result in the formation of compounds such as 4a,4b-dihydrophenanthrene, and trihydroxydihydrophenanthrene through photocatalytic processes. Additionally, glycosides are more sensitive to light than their aglycones, making them more susceptible to cis isomerization and the subsequent formation of a phenanthrene ring. Studies have shown varying half-lives for different trans-stilbenes in vitro. Other than photosensitivity, pH, and higher temperatures also affect the stability of stilbenes due to their unstable hydroxyls and C-C double bond. Resveratrol remains stable at room or body temperature under acidic conditions but degrades rapidly under alkaline pH. Oxygen plays a vital role in this degradation process. To enhance stability, it is imperative to reduce the pH and temperature, and strictly limit exposure to oxygen and light [9,30].

The term “stilbene” is derived from the Greek word “στίλβω”, which means shining. This unique property of stilbenes plays a key role in their identification and structure determination. When these compounds are exposed to ultraviolet light, they exhibit fluorescence. Most natural stilbenes emit fluorescence at 370–400 nm. For instance, trans-resveratrol and its derivatives, demonstrate their highest absorption peak in the ultraviolet (UV) region, specifically within the range of 300 to 330 nm. Notably, trans-stilbenes, including trans-resveratrol, exhibit peak absorbance at 304–308 nm, while cis-resveratrol absorbs at a slightly lower λmax of 286 nm. The glycoside of resveratrol, trans-piceid, presents a λmax of 318.4 nm, with its cis-isomer at 284.4 nm. Additionally, the absorbance ratio at 290 and 310 nm can be utilized to ascertain the correlation between cis- and trans-oxyresveratrol. The addition of hydroxyl groups on the structure in different positions slightly changes these spectrophotometric values. In the case of *trans*-piceatannol (hydroxyls at R3′, R4′, R3 and R5), its maximum emission is recorded in the range of 400 nm [66,67], oxyresveratrol (hydroxyls at R2′, R4′, R3 and R5) is another stilbene with its λem in the range of 400 nm [68]. By contrast, gnetol (hydroxyls at R2′, R6′, R3 and R5) has a different emission spectrum with a main peak in the range of 503 nm and a secondary peak at 390 nm [9]. Other stilbenes with fewer hydroxyl groups, like pinosylvin (hydroxyls at R3 and R5) and pterostilbene (hydroxyl at R4′), were reported to emit at a lower wavelength. As a result, the variations in the structural composition of stilbenes unequivocally lead to pronounced changes in their spectrophotometric properties, which are absolutely critical for definitively determining the chemical structures of various stilbenes, even offering valuable insights into the molecule’s degradation level, stability, and structural changes [9,69].

The process of substituting various functional groups, such as hydroxyl (-OH) and methoxy (-OCH₃), into the fundamental structure of stilbene at different positions and in varying quantities gives rise to a wide array of derivatives, each exhibiting unique physicochemical properties. Numerous studies focusing on the biological activities of stilbenes have revealed different results. For example, in vitro antioxidant effects of these derivatives have revealed a notable trend: the antioxidant activity tends to escalate with an increasing number of hydroxyl groups present in the structure. However, this beneficial effect diminishes when hydroxyl groups undergo methylation or when they form heterosides, leading to differences in performance. Particularly significant findings from the research indicate that the positioning of hydroxyl groups, especially at the C-4′ position and in close proximity to another -OH group at the ortho position, plays a crucial role in enhancing antioxidant activity [9,70,71]. Additionally, methylation can either enhance or diminish the activity of stilbene derivatives. For instance, when assessing aglycone compounds for their antimicrobial properties, the stilbene derivatives, characterized by their higher lipophilicity and the presence of methoxy substitutions, demonstrate superior effectiveness compared to compounds which contain solely hydroxyl groups [9,72]. Similarly, data indicate that compounds exhibiting high lipophilicity demonstrate more potent anticancer and/or cytotoxic activity, which has been proven by some studies, such as pterostilbene, which was found to be more effective than resveratrol. It could be suggested that the bioavailability of pterostilbene is higher than that of resveratrol, which could be the reason for the observed higher activity. Methoxylated stilbenes show increased lipophilicity, enhancing bioavailability and making them promising for clinical trials. Moreover, ortho-hydroxylated stilbenes may offer superior antioxidant, anti-inflammatory, and anticancer properties compared to meta-hydroxylated ones, likely due to a stable semiquinone radical formation [6,9,44,73,74].

Hydroxylated and glycosylated stilbenes have improved aqueous solubility, aiding their integration into hydrophilic matrices, but their overall low solubility limits high concentration. On the other hand, glucosidic forms of stilbene compounds exhibit higher polarity, which also contributes to enhanced solubility in water, addressing a significant challenge associated with the therapeutic application of stilbenes: low bioavailability. The presence of stilbenes in heterosidic form alters their physical, chemical, and biological properties, thereby enhancing their average lifespan and solubility in aqueous media. This transformation offers greater bioavailability and helps to reveal higher biological activities. Typically, stilbenes undergo glycosylation; for example, polydatin, in its glycoside form, can reach concentrations nearly six times higher than that of resveratrol [9,75].

Research into stilbenes with oligomeric structures has shown considerable promise, particularly regarding neurological health. A notable study examined alpha viniferin, a dimeric stilbene derivative, and demonstrated its activity in a fractionation study focused on acetylcholinesterase (AChE) activity. This finding suggests that oligomeric stilbene derivatives could provide valuable insights into their biological effects, especially in relation to AD. However, the limited number of studies investigating dimeric and other oligomeric forms of stilbenes highlights a significant gap in the literature, indicating that this area deserves further exploration [63].

## 5. Biosynthesis of Stilbenes

**Figure 7:**
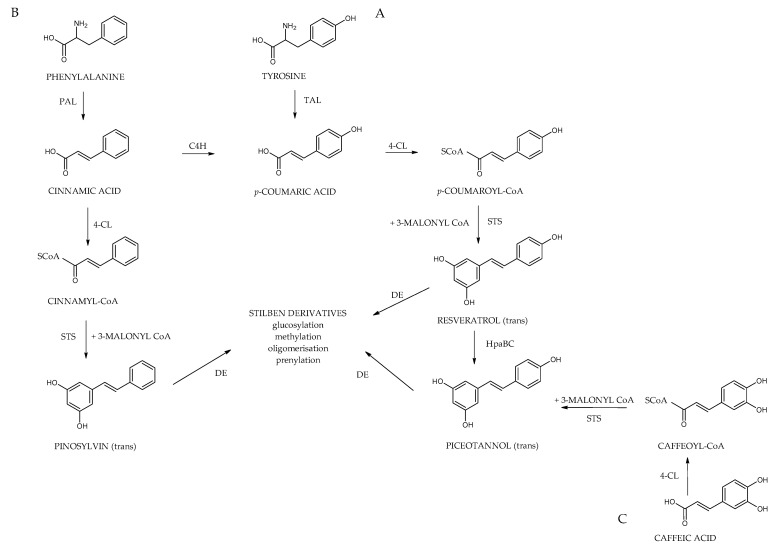
Biosynthetic pathway of stilbenes; phenylalanine ammonia-lyase (PAL) and tyrosine ammonia-lyase (TAL); cinnamate 4-hydroxylase (C4H), coumarate CoA ligase (4-CL), stilbene synthase (STS), HpaBC.

Plants produce stilbenoids, as a defense mechanism in response to viruses, fungi, and bacteria attacks. These compounds are derived from the phenylpropanoids pathway, illustrated in Figure 7. The shikimate pathway is a specific metabolic pathway for stilbenes. This process begins with the conversion of L-tyrosine or L-phenylalanine into *p*-coumaric acid or cinnamic acid achieved by enzymes called tyrosine ammonia lyase (TAL) or phenylalanine ammonia lyase (PAL). These pathways are shown in Figure 7 as “A” and “B”. Additionally, cinnamic acid can be further transformed into *p*-coumaric acid by the enzyme cinnamate 4-hydroxylase (C4H). Then, the molecules are converted into cinnamoyl-CoA or *p*-coumaryl-CoA by an enzyme called coumarate CoA ligase (CL). CoA ligase turns *p*-coumaric acid into *p*-coumaryl CoA, which interacts with three molecules of malonyl CoA. The synthesis of stilbenes in plants involves combining one CoA-ester of a cinnamic acid derivative and three malonyl-CoAs by utilizing stilbene synthase (STS) and resulting in a polyketide intermediate from one cinnamoyl-CoA or *p*-coumaryl-CoA as an important step in the synthesis of stilbenes. STS (stilbene synthase) is a vital enzyme involved in plant metabolism. The tetraketide formed undergoes an aldol reaction by stilbene synthase and forms resveratrol. However, the distribution of stilbenes in the plant kingdom is limited, as the enzyme STS is not present in most plant species. For synthesis of trans-resveratrol, stilbene synthase is necessary as well as STS can modify stilbenes through glycosylation, methylation, or prenylation, diversifying the range of biologically active compounds [6,36].

Additionally, cinnamic acid can be further transformed into *p*-coumaric acid by the enzyme cinnamate 4-hydroxylase (C4H). Then, the remaining part is carried out the same way as A; the molecule is converted into *p*-coumaric-CoA by an enzyme called coumarate CoA ligase (CL). It catalyzes the aldol condensation reaction, a key step in the biosynthesis of stilbenoids. Stilbene-2-carboxylic acid, a crucial intermediate, is produced due to this reaction. This molecule is a precursor for synthesizing various stilbenoids, including the well-known compound trans-resveratrol, recognized for its potential health benefits [6,29].

Subsequently, Cinnamyl-CoA and coumaroyl-CoA are transformed into the monomeric stilbenes pinosylvin and resveratrol. These stilbenes act as the building blocks for various other stilbenes, undergoing modifications such as methylation, hydroxylation, glycosylation, isomerization, and oligomerization. Additionally, the way “C”, as shown in Figure 7, uses similar enzymes, caffeic acid serves as the precursor of piceatannol, which is another monomeric origin of certain stilbenes. Cinnamate-4-hydroxylase (CL) is a crucial enzyme involved in plant metabolism. Its main function is to catalyze the conversion of caffeic acid to caffeoyl-CoA, a central metabolite in the biosynthesis of various phenolic compounds. An interesting hypothesis suggests that stilbene synthase (STS) utilizes caffeoyl-CoA as a substrate for producing piceatannol, which broadens the possible metabolic pathways for synthesizing biologically active plant compounds [9].

**Figure 8:**
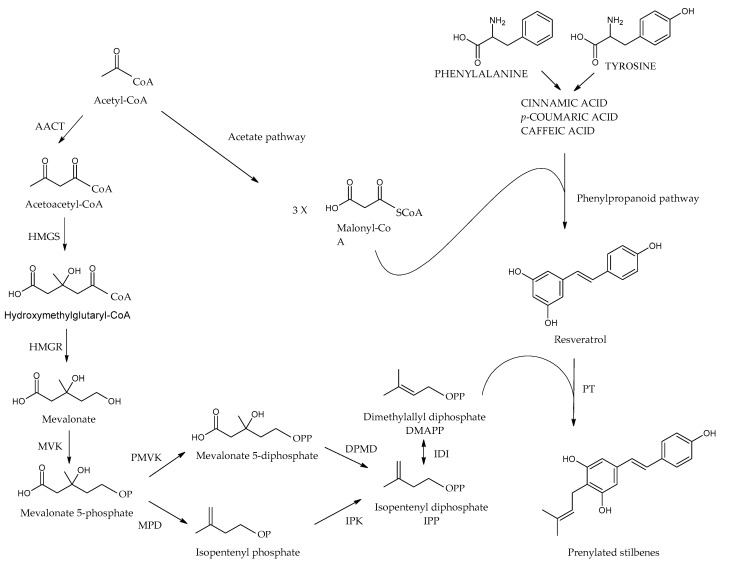
Prenylated stilbenes biosynthesis. AACT: acetoacetyl-CoA thiolase, HMGS: 3-hydroxy-3-methylglutaryl-CoA synthase, HMGR: 3-hydroxy-3-methylglutaryl-CoA reductase, MVK: mevalonate kinase, PMVK: phosphomevalonate kinase, MPD: mevalonate phosphate decarboxylase, DPMD: Diphosphomevalonate decarboxylase, IPK: isopentenyl phosphate kinase, IDI: IPP/DMAPP isomerase, PT: prenyltransferase.

Due to structural differences in prenylated stilbenes biosynthesis of these structures involves different steps. Similarly, to the stilbenes phenylpropanoid pathway is known as the basic as well the acetyl coenzyme A pathway contributes (Figure 8). Resveratrol is synthesized by stilbene synthase, which is part of the mevalonate pathway, and the prenyl group is derived from acetyl CoA. The last step which catalyzes by prenyltransferase enzyme involves addition of prenyl/geranyl substitution to the stilbene main cycle to form prenylated resveratrol [43,45,76,77].

## 6. Alzheimer Disease

Alzheimer’s disease (AD) is the most prevalent neurodegenerative disorder and the leading cause of dementia in developed countries [78]. It is anatomically characterized by early involvement of the hippocampus and entorhinal cortex, with progressive memory loss and functional decline resulting from cerebral atrophy [79]. As the disease advances, symptoms such as apathy, depression, impaired communication, disorientation, judgment difficulties, dysphagia, gait disturbances, and behavioral changes become increasingly apparent. The onset and progression of symptoms are influenced by factors such as age, genetics, and sex [80].

Since the first pathological description by Alois Alzheimer in 1906, the identification of extracellular SPs and intracellular NFTs has remained central to AD diagnosis [81,82]. Today, AD accounts for approximately 75% of all dementia cases, affecting more than 50 million individuals globally. With aging as the major risk factor and life expectancy continuing to rise, this number is projected to exceed 106 million by 2050 [83,84,85].

Epidemiological data indicate a sharp increase in AD prevalence after age 65, with women disproportionately affected across all age groups [86]. The associated medical, social, and economic burdens have grown accordingly, creating major challenges for both healthcare systems and families [78,86,87]. Following diagnosis, average life expectancy ranges from 5 to 8.5 years, during which time patients often require increasing levels of care and experience substantial declines in quality of life [87]. Furthermore, it is imperative to acknowledge that in advanced stages, patients experience a significant deterioration in their quality of life and become incapable of independently sustaining their daily lives [87].

### 6.1. Etiology of Alzheimer’s Disease

While the etiology of AD remains incompletely elucidated, it is postulated to be multifactorial, with risk factors playing a pivotal role in its development [87]. Genetic cases, referred to as familial AD, constitute less than 3% of all AD subtypes and manifest earlier (approximately 10–12 years earlier) compared to idiopathic AD forms known as sporadic AD [88]. Mutations accountable for familial AD with a dominant inheritance pattern occur in genes such as the amyloid precursor protein (APP), presenilin 1, and 2, which induce the formation of Aβ peptides [89]. Among other mutations predisposing to an increased risk of AD dementia are mutations in the APOE gene, with the APOE4 variant being a predisposing factor [90]. In addition to genetic factors, other contributors to AD development include hypertension, obesity, hypercholesterolemia, sedentary lifestyle, tobacco use, low educational level, diabetes mellitus, or hyperinsulinemia [91,92,93,94,95].

### 6.2. Pathophysiology of Alzheimer’s Disease

AD is pathologically defined by widespread neuronal and synaptic loss, which varies across brain regions and contributes to diverse clinical presentations. Neuropathological hallmarks include intracellular NFTs, composed of hyperphosphorylated tau protein, and extracellular Aβ plaques, commonly referred to as SPs [96,97]. These abnormalities are often accompanied by neuroinflammation, progressive neuronal death, and cerebral atrophy, particularly in regions involved in memory, such as the hippocampus and entorhinal cortex [98,99].

Notably, the accumulation of Aβ and tau begins years before clinical symptoms emerge, with a latent phase lasting approximately 10 to 12 years [98]. While SPs are widely distributed throughout the affected brain regions, NFTs tend to localize in areas essential for cognitive function. The presence and distribution of these lesions form the basis of neuropathological diagnosis, typically confirmed postmortem [96]. The amyloid cascade hypothesis remains a prevailing model explaining disease initiation, suggesting that Aβ accumulation triggers downstream events that contribute to the development and progression of AD dementia [96].

#### 6.2.1. Amyloid-β Pathology

SPs, composed of extracellular Aβ deposits, are one of the primary pathological features of Alzheimer’s disease (AD) and are strongly associated with synaptic loss and neuronal degeneration [76,91]. Aβ is a peptide of 39–43 amino acids, generated through the proteolytic cleavage of amyloid precursor protein (APP) by β- and γ-secretase complexes, with presenilins as key catalytic components [94,95]. Under physiological conditions, the non-amyloidogenic pathway involving α-secretase prevents Aβ formation. Among Aβ isoforms, Aβ40 and Aβ42 are the most common, with Aβ42 being the most fibrillogenic and neurotoxic [96,97].

Aβ monomers tend to oligomerize in the extracellular space, forming dimers and trimers, which aggregate into SPs. This accumulation impairs synaptic function by disrupting neurotransmitter receptor activity and ion channel balance, leading to defective signal transmission between neurons [99,100]. As Aβ levels increase, synaptic density declines, ultimately contributing to cognitive impairment, which is more closely correlated with synaptic loss than with plaque burden alone [101,102,103].

While Aβ accumulation is an early event in AD pathology, it does not always correlate with immediate neuronal atrophy. Tau pathology, in contrast, is more directly linked to neuronal death and brain volume loss [104]. Aβ clearance occurs through vascular excretion and enzymatic degradation, processes that are often impaired in AD. Aβ40 is particularly associated with cerebral amyloid angiopathy (CAA), which disrupts vascular perfusion and increases the risk of cerebral ischemia [105,106,107,108].

Neuronal enzymes such as neprilysin and insulin-degrading enzyme (IDE) play important roles in Aβ degradation. Reduced IDE activity has been observed in AD, and competitive degradation between insulin and Aβ may explain the pathological overlap with type 2 diabetes [109,110,111,112]. Experimental models have shown that enhancing IDE activity can reduce Aβ accumulation and mitigate AD pathology [113,114,115,116], suggesting that upregulation of IDE may represent a potential therapeutic strategy.

#### 6.2.2. Tau Pathology

Tau is a microtubule-associated protein predominantly expressed in the central and peripheral nervous systems. It binds to tubulin to support microtubule assembly, contributing to neuronal structure, axonal transport, and synaptic plasticity [101,117]. In its normal state, tau stabilizes axons by maintaining microtubule integrity. However, abnormal phosphorylation—especially hyperphosphorylation—disrupts this function, leading to microtubule destabilization, synaptic dysfunction, and eventually neuronal death. Hyperphosphorylated tau also propagates between neurons, further promoting tau pathology.

NFTs, composed of aggregates of hyperphosphorylated tau, accumulate within the soma and proximal dendrites of neurons [118,119]. Their presence is strongly associated with the clinical severity of Alzheimer’s disease and is considered the neuropathological marker most closely linked to cognitive decline [120]. Tau pathology typically originates in the anterolateral region of the entorhinal cortex—responsible for episodic memory—and gradually spreads to the hippocampus. In contrast, the posteromedial region of the entorhinal cortex, which is involved in spatial memory, is affected at later stages.

#### 6.2.3. Neuroinflammation and Neuron Loss

In addition to Aβ and tau pathologies, neuroinflammation and oxidative stress are major contributors to the neurodegenerative processes observed in AD [121]. While inflammation serves as a protective mechanism, its chronic activation leads to tissue damage [122]. Microglial cells, the resident immune cells of the central nervous system, play a key role in mediating the inflammatory response. In AD, alterations in microglial morphology and distribution—driven by elevated levels of cytokines and pro-inflammatory mediators—are widely documented [123].

Microglia act as phagocytes, removing toxic substances and maintaining homeostasis [81]. However, in AD, microglial activation occurs even before Aβ plaque formation and persists throughout disease progression [124]. Although initially capable of encircling plaques, microglia fail to eliminate them, resulting in sustained inflammatory activity. This chronic activation promotes a pro-inflammatory cytokine environment that exacerbates oxidative stress and contributes to neuronal damage and death [125]. Moreover, prolonged inflammation impairs Aβ clearance, further accelerating disease progression [126].

Oxidative stress, characterized by excessive production of reactive oxygen species (ROS), damages lipids, proteins, and nucleic acids, overwhelming the brain’s antioxidant defense systems [127]. A reduction in antioxidant enzyme activity has been linked to increased neuronal vulnerability and cell death [128]. Ultimately, the progression of AD involves a convergence of Aβ accumulation, tau pathology, sustained neuroinflammation, and oxidative stress, all of which lead to neuronal death and brain atrophy, particularly in the hippocampus and cerebral cortex [97,121].

### 6.3. Current Pharmacological Treatments

Current therapeutic approaches to Alzheimer’s disease (AD) aim not only to alleviate symptoms but also to modify the underlying disease mechanisms. Non-pharmacological strategies, such as lifestyle interventions and cognitive training, are considered essential for delaying the progression of cognitive impairment [129,130,131,132,133]. On the pharmacological front, treatments are increasingly focusing on disease-modifying agents that target Aβ accumulation, tau protein aggregation, synaptic dysfunction, neuroinflammation, and metabolic disturbances [134]. Among these, monoclonal antibodies (mAbs) designed to target extracellular tau aim to limit its intercellular spread and inhibit neurofibrillary tangle formation [135].

Despite the emphasis on Aβ in drug development, many anti-Aβ agents have failed to yield significant cognitive benefits in clinical trials [136]. Passive immunotherapy using anti-Aβ mAbs is regarded as one of the most promising approaches due to its selectivity and tolerability [137,138]. However, currently available pharmacological options largely offer symptomatic relief rather than halting disease progression [139]. The potential for Aβ-targeted therapies to slow disease progression underscores the importance of early intervention [138,140].

In this context, natural compounds such as stilbenes have drawn growing attention due to their multi-target neuroprotective properties and potential role in modifying early-stage pathologies.

## 7. Potential Benefits and Limitations of Natural Stilbenes in AD

### 7.1. Potentitail Benefits

#### 7.1.1. Resveratrol

Among the stilbenes, resveratrol (Figure 3), which attracts the most attention with its neuroprotective properties, has been found in many plants from red grapes to cranberries, peanuts, and blueberries. It may ameliorate AD by reducing Aβ generation and oligomerization, increasing Aβ clearance, and regulating tau neuropathology by preventing aberrant tau phosphorylation and aggregation. ROS, hydrogen peroxide free radicals, NO, and other intracellular and extracellular toxins associated with neurodegenerative disorders have all been linked in studies to resveratrol’s protective action on neurons. Particularly notable for its anti-inflammatory qualities, resveratrol shields microglia and astrocytes from Aβ-induced neuroinflammation and toxicity and has been linked to protective mechanisms [14].

Molecular investigation of resveratrol interactions with fibrils and Aβ_42_ peptide revealed that the aggregation-inducing hydrophobic segments of Aβ_42_ peptide are protected by resveratrol [141]. By specifically targeting proteins involved in proteostasis, resveratrol at 100 µM mitigates the toxicity of Aβ in the *Caenorhabditis elegans* AD model, thereby reducing the quantity of aggregated Aβ [142]. Al-Edresi et al. discovered that resveratrol inhibits Aβ_1-42_ aggregation by fragmenting it into smaller peptides [15]. Resveratrol treatment for 20 h reduced the peak height of the Aβ_1-42_ peptide monomer in HPLC analysis, indicating a drop in monomeric Aβ_1-42_ peptide levels. It was observed that in the resveratrol-containing group, the Aβ_1-42_ monomer vanished more quickly than in the resveratrol-free group. It was also proved in Aβ_1-42_ induced SH-SY5Y (human neuroblastoma cells) that resveratrol prevents cells from the cytotoxic effects of Aβ_1-42_ [15]. Another study revealed that the loss of residue from the N-terminus of the Aβ_1-42_ peptide resulted in a lipophilic peptide and enhanced retention time. In contrast, the loss of residue from the C-terminus resulted in peaks with diminished retention time [143]. The β-secretase activity was found to be considerably reduced by oligomeric resveratrol from *Paeonia lactiflora* and resveratrol (IC_50_ 11.9 µM) was also shown to inhibit β-secretase [144]. Resveratrol and its analogs were also proven to be inhibitors of BACE-1. According to the results of Koukoulitsa et al. BACE-1 inhibitory activity of resveratrol was found to be 28 µM [145]. In a different study it was determined that resveratrol suppressed Aβ_1-42_ aggregation, the resveratrol concentration, which reduced aggregation by 50%, was reported as 40.8 nM [146].

The impact of resveratrol on the formation of amyloid plaques was investigated in another in vivo study. Resveratrol at a dose of 60 mg/kg for sixty days was given orally to Tg6799 mice, which express the human APP and PS1 genes. The treatment group’s hippocampal regions exhibited a notable decrease in amyloid plaques along with decreased levels of Aβ_42_ and Aβ_40_ in contrast to the control group. The Western blot (WB) method examined the underlying mechanism, and a significant decrease in APP, sAPPα, sAPPβ, and BACE1 (β-secretase) expression levels was detected. Interestingly, no change in SIRT1 (Sirtuin 1) levels was observed [14]. However, it has previously been stated that resveratrol activates SIRT1 in the SAMP8 age-related AD mouse model. WB examination of the cortex and hippocampus of the two groups demonstrated that the resveratrol group mice had higher SIRT1 levels than the control group, and there was a decrease in the acetylation of p53, SIRT1’s substrate, in the cortex and hippocampal regions. Furthermore, increased amounts of phosphorylated AMPK (p-AMPK) were discovered in the cortex region of the resveratrol group, but no changes were observed in AMPK levels. One of the most important results can be summarized as the presence of scarcely any Aβ granules in the resveratrol group and the detection of a few Aβ_42_ and Aβ_40_ granule clusters in the control group. According to the experimental results, in which the levels of two enzymes tasked with the amyloidogenic/non-amyloidogenic processing of APP, namely α- and β-secretases, were measured, no change was observed in the β-secretase enzyme in the resveratrol group, while an increase in the α-secretase enzyme was detected in both the cortex and hippocampus. Another indicator of the study was the rise in the amounts of GSK3β phosphorylated at Ser9; this was reflected in the decreased pTau levels in the resveratrol-fed group [147]. In p25 transgenic mice neuroprotection of resveratrol (5 μg/μL, 3 weeks, i.c.v injection) linked with reduced acetylation of the known SIRT1 substrates PGC-1α and p53. Lower levels of the apoptotic marker activated caspase 3 and an astrogliosis marker, GFAP, indicated that resveratrol decreased neurodegeneration in the CA1 and CA3 areas of the hippocampus [148]. Sirtuins are deacetylases that have been identified in experimental animal studies to have anti-aging characteristics as well as stress resistance. SIRT1 regulates cell processes by deacetylating key substrates such as p53, FOXO transcription factors, PGC-1α, NFκB, and others, which are connected to age-related illnesses [149]. SIRT1 induction may play an essential part in the life-prolonging effects of calorie restriction, and resveratrol is thought to replicate the effect [150,151]. Two distinct investigations have revealed that resveratrol’s effects on SIRT1 initiation occur through the CamKKβ-AMPK pathway by inhibiting phosphodiesterases particular to cAMP [152,153]. In a different study, a group of male C57Bl/6J mice was used to test the metabolic impact of resveratrol. The mice were fed a chow diet or a high-fat (HF) diet for 15 weeks, at a dose of 200 or 400 mg/kg/day resveratrol. As a result of the examination in the gastrocnemius muscle, it was determined that resveratrol significantly induced PGC-1α mRNA, which in turn translated into an increase in PGC-1α protein. An increase in PGC-1β, mitochondrial transcription factor A (Tfam), and a target of NRF-1 has also been observed. To investigate if SIRT1 and PGC-1α mediate Resveratrol (RSV)’s impacts on mitochondrial function, scientists co-infected C2C12 myotubes with an adenovirus that expressed PGC-1α along with a control or a targeted short hairpin RNA (shRNA) that was directed against SIRT1. The study found that high PGC-1α expression remained but endogenous SIRT1 levels were significantly lowered. It has been interpreted that resveratrol is effective against diet-induced obesity and insulin resistance and SIRT1 is a crucial regulator of energy and metabolic homeostasis [154]. Although there are different studies in which resveratrol upregulates SIRT1 [155,156,157,158]. Dasgupta and Milbrandt also reported that it activates SIRT1-independent AMP kinase in neurons. Neuro2a cells were treated with 10 μM resveratrol for 2 h, either with or without 10 μM sirtinol and splitomycin, and 10 mM nicotinamide (SIRT1 inhibitors). Resveratrol’s substantial activation of AMPK, as evidenced by increased phosphorylation of AMPK and its downstream target acetyl-CoA carboxylase, was unaffected by any of the SIRT1 inhibitors. Similarly, SIRT1 inhibitors did not affect resveratrol’s ability to enhance Neuro2a neurite outgrowth. These findings further demonstrated that resveratrol’s effects on AMPK were independent of SIRT1 activation [159].

Microglia, the predominant resident immune cells of the central nervous system (CNS), are intimately associated with neuroinflammatory priming in the brain, which happens after repeated external assaults and stress. Furthermore, a variety of age-related neurodegenerative disorders involve cognitive impairments associated with an imbalance in the neuroinflammatory response coming from microglia. Lowered SIRT1 levels in microglia upregulate IL-1β, leading to cognitive decline in mice with age [160]. In a study supporting these findings, it was discovered that resveratrol was useful in LPS-stimulated cell lines and that it contributed to the growth, proliferation, and apoptosis of mouse microglia cell lines (N9 cell line) by activating SIRT1. The inhibitory effect of resveratrol (15 and 30 μM) on the upregulation of MMP-9, iNOS, IL-1β, and IL-6 was diminished in N9 cells that had SIRT1 expression knocked down [161]. In a separate investigation, it was demonstrated that resveratrol (5–20 μM) decreased the SIRT1 Michaelis constant for both acetylated substrate and NAD (+) and increased the longevity of *Saccharomyces cerevisiae* cells by inducing SIRT1-dependent p53 deacetylation [162].

Price et al. demonstrated that mice overexpressing SIRT1 (without receiving resveratrol treatment) exhibited significant increases in AMPK activity in the skeletal muscle. SIRT1 knockout mice (6-month-old C57BL/6J) did not exhibit any of these effects, even after receiving 8 months of treatment with resveratrol at doses of 25 mg/kg/day and 215 mg/kg/day. Furthermore, unlike SIRT1 knock-in (KI) mice, resveratrol treatment did not significantly enhance mtDNA, PGC1-*α* levels, or mitochondrial respiration in the absence of SIRT1. Consequently, even though Price et al.’s study concentrated on skeletal muscle, the results indicate that SIRT1, both by itself and in response to resveratrol activation, is essential for AMPK-mediated enhancements in mitochondrial health and function, which may also apply to the brain, emphasizing SIRT1 as a major target for resveratrol’s neuroprotective benefits [156].

According to reports, Aβ accumulation can lead to AD pathology when there are abnormalities in the activity of Aβ-degrading enzymes, such as plasmin, neprilysin (NEP), IDE, angiotensin-converting enzyme (ACE), and endothelin-converting enzyme. Melzig and Escher (2002) reported that cellular enzyme activity of NEP and ACE linked to an inhibition of cellular proliferation was induced by long-term incubation of the SK-N-SH cells with quercetin, resveratrol, and a combination of both phenolics for 4 days at concentrations lower than necessary for inhibition of NEP and ACE activity [163]. In a different study, resveratrol, at 30 µM concentration, revealed a significant decrease in protein secretion only in the terminal step of the endothelin-1 pathway in the HUVEC cell line and reduced endothelin-converting enzyme-1 mRNA levels [164]. Since neprilysin is primarily affected by estrogen, the effect of resveratrol in regulating the estradiol and neprilysin pathways in the LPS model of AD was investigated. According to the experimental procedure, mice induced with 0.8 mg/kg LPS were given i.p. 4 mg/kg resveratrol for 7 days. In light of the experimental findings, a significant increase in estradiol and NEP levels was observed, and the decrease in different types of memory was reversed by resveratrol [165]. The tissue-type plasminogen activator-plasmin (t-PA) system helps remove Aβ in mice brains and prevents neurotoxicity, suggesting a link to late-onset AD, also AD risk has been linked to urokinase-type plasminogen activator (u-PA) gene polymorphisms [166]. It was determined that resveratrol applied to HUVEC cells at a dose of 10 µM increased t-PA and u-PA antigen levels. At the same time, a significant increase in t-PA and u-PA mRNA levels was found, 5- to 6-fold and 3- to 4-fold, respectively [167].

Aβ deposits in AD are richer in Cu and Zn, which are soluble in vitro by Cu/Zn-selective chelating agents. In AD transgenic mice it has been shown that Cu/Zn-selective chelating agent clioquinol (antibiotic) reduced Aβ accumulation in the brain by 49% [168]. According to reports, resveratrol chelates copper and prevents human low-density lipoproteins (LDL) from oxidizing. As a result, resveratrol-induced increases in cysteine may assist lower plaques by chelating copper, as has been demonstrated in studies on Cu/Zn chelators [169,170].

In the study examining the effects of stilbenes, trans/cis-resveratrol mixture, oxyresveratrol, veraphenol, and cis-scirpusin A on BACE1 inhibition, the IC50 values were found to be 1.5 × 10^5^, 7.6 × 10^6^, 4.2 × 10^6^, and 1.0 × 10^5^ M, respectively. The stilbenes appeared to be rather specific BACE1 inhibitors because they showed reduced inhibition of α-secretase (TACE) and other serine proteases such as chymotrypsin, trypsin, and elastase [171]. Conversely, data from Marambaud et al. reveals that resveratrol encourages intracellular breakdown of Aβ by a process involving the proteasome rather than inhibiting the formation of Aβ because it has no impact on the enzymes that produce Aβ, β- and γ-secretases. They highlighted this mechanism in their research using APP695 (transmembrane precursor of Aβ)-transfected HEK293 cells and neuroblastoma N2a cells. After 24 h of incubation, 20–40 µM resveratrol significantly lowered total secreted Aβ levels, including secreted Aβ_40_ and Aβ_42_ in HEK293 cells. It was determined that it also inhibited total secreted Aβ in N2a cells at the same concentration. Resveratrol showed no influence on α, β, or γ-secretase-mediated cleavages of APP, APP C-terminal fragments C99, C89, and C83, or the APP intracellular domain. These results suggest that resveratrol has no effect on the generation of Aβ [172]. In a later study, the same team further defined the signaling pathway involved in the control of Aβ levels by resveratrol. One of the key actions of resveratrol (10, 20 30, and 40 µM) is to target AMPK and boost its phosphorylation at Thr-172 in HEK293 cells. Resveratrol activated AMPK, inhibited mTOR, and initiated autophagy and lysosomal clearance of Aβ. Similarly, in N2a cells, a robust increase in the phosphorylation of AMPK and ACC resulted in the reduction in Aβ including Aβ_1–40_ and Aβ_1–42_ with resveratrol. After determining that AMPK plays a role in the effect of resveratrol on Aβ levels in cell culture research, a similar technique was carried out in an in vivo investigation. The effectiveness of resveratrol on Aβ and amyloid accumulation in APP/PS1 mice was also determined. After 15 weeks of feeding mice 0.4% resveratrol, the levels of soluble and insoluble Aβ_1-40_ (30%) and insoluble Aβ_1-42_ (25%) in total brain homogenates decreased significantly. Resveratrol administered orally passes the blood–brain barrier, activates brain AMPK, and lowers Aβ levels and accumulation in the cerebral cortex, confirming the in vitro findings [173]. In another study, 45-day-old Tg19959 mice’s diets were supplemented with 0.2% resveratrol, and it was found that resveratrol intake reduced both the quantity and area of plaques in various brain regions, with the exception of the hippocampus region. They noted that these effects occurred without noticeable activation of SIRT-1 or alterations in APP processing. Lower plaque formation has been linked to elevated cysteine and diminished GSH levels [174].

A supporting in vivo study was conducted in Tg2576 mice with Cabernet Sauvignon wine containing 0.2 mg/L resveratrol. The findings indicate that Cabernet Sauvignon has a positive effect via boosting non-amyloidogenic processing of APP, hence avoiding the generation of Aβ peptides [175]. According to consistent epidemiological research, moderate red wine drinking is linked to a lower risk of dementia and AD [176,177].

Research reveals that neuroprotectin levels are decreased in AD patients, which correlates with greater neuroinflammation and neuronal damage. Its decline may exacerbate the progression of AD pathology, including the accumulation of Aβ plaques and NFTs, hallmark features of the disease. Its decrease could accelerate the development of AD pathology, which includes the build-up of the disease’s signature Aβ plaques and NFTs [178]. Several studies have clarified the neuroprotective properties of various stilbene compounds. It was discovered that 50 μM catechin and 10 μM resveratrol or 25 μM resveratrol and 10 μM catechin were protective in the study examining the possible synergistic protective effects of resveratrol and catechin against Aβ-AP_1–41_-induced Aβ toxicity in PC12 cells. A concentration-dependent response was found for both resveratrol and catechin in protection against ROS toxicity [179]. In a similar study, the neuroprotective effects of resveratrol were observed in the same cell line. Resveratrol (25 μM) reduced intracellular ROI formation, inhibited PARP cleavage and JNK phosphorylation, and increased expression of Bcl-X_L_ therefore preventing Aβ_25–35_ induced cytotoxicity [180]. Rhaponticin isolated from Rhei rhizome and its aglycone metabolite, rhapontigenin, were tested in IMR-32 (human neuroblastoma cells) against Aβ_1-42_-induced toxicity. With the treatment of this stilbenes at 30 µM bcl-2 expression was upregulated and the proapoptotic bax gene was downregulated [181].

Resveratrol clearly causes dephosphorylation of the microtubule-associated protein Tau both in vitro and in vivo, according to Schweiger et al. In the in vitro analysis resveratrol (100 µM) interferes with the MID1 (Midline 1) complex assembly and reduces the MID1 transcript and protein level in the HEK293T cell line. Inhibiting the MID_1-α4_ complex, which triggers the degradation of the catalytic subunit of Protein Phosphatase 2A (PP2Ac), could lead to new therapy options for AD. Resveratrol also raises PP2A activity and dephosphorylates Tau at PP2A-sensitive locations in primary cortical neurons. In in vivo studies, resveratrol was administered intraperitoneally (daily) to wild-type mice at a dose of 25 mg/kg for two weeks. Resveratrol-treated animals showed a substantial reduction in Tau phosphorylation at epitope S202, in line with the cell culture models [182]. Resveratrol also was found to drastically reduce tau aggregation and cytotoxicity brought on by tau oligomers in Sun et al.’s study. It also prevented N2a cells from absorbing external tau oligomers. For five weeks, 6-month-old male PS19 mice were given either 40 mg/kg body weight of RSV or a vehicle orally once daily. RSV treatment successfully restored cognitive deficits by lowering the levels of phosphorylated tau, neuroinflammation, and synapse loss in the mice’s brains [183]. Tau hyperphosphorylation is mediated by several kinases in the brain. Porquet et al. examined the effect of resveratrol against abnormal tau phosphorylation in the SAMP8 brain, which is achieved by activating various Tau kinases such as CDK5, GSK3β, or JNK. SAMP8 mice were fed with the standard or resveratrol (1 g/kg) supplemented diet for seven months. The findings show that resveratrol therapy reduces the activity of two major tau kinases in AD, CDK5, and GSK3β, in the cortex of SAMP8 mice, and that tau phosphorylation in Ser^396^ is prevented by inhibiting these tau kinases. However, no discernible alterations in JNK were discovered [147]. In a recent study investigating the effects of resveratrol and donepezil on Aβ and neurofibrillary tangle (NFT) accumulation in an AD rat model, the combination of resveratrol and donepezil significantly reduced Aβ and NFT accumulation in both treatment and prophylaxis groups. The best results were obtained with prophylactic resveratrol 10 mg and the resveratrol + donepezil combination, demonstrating resveratrol’s neuroprotective and synergistic effects [184]. The mechanisms that show how resveratrol has fulfilled its potential in preclinical investigations on AD are listed in Table 1.

Various research on the effects of resveratrol on Aβ and tau have produced diverse findings. Resveratrol seems to lower Aβ accumulation on the one hand; its effects on direct Aβ generation or interfering enzymes are not very clear. One study found that resveratrol lowered Aβ levels by means of increased Aβ degradation via the proteasome, not stopping Aβ generation [172]. Resveratrol thereby guarantees later clearance of Aβ without suppressing Aβ synthesis. Conversely, several investigations have shown that resveratrol inhibits enzymes that generate Aβ, therefore preventing the formation of the molecule. These contradicting findings imply that the effect of resveratrol against Aβ pathogenesis is complicated and can change depending on the context. The dosage and method of administration will affect the possible effects of resveratrol. Resveratrol has shown protective effects against tau oligomers, lowers hyperphosphorylation of tau, and inhibits tau aggregation, according to many research. Resveratrol, several of these studies contend, does not directly influence tau’s phosphorylation or synthesis [182,183]. For instance, although the Schweiger et al. (2017) study underlined that resveratrol dephosphorylates tau phosphorylation, the Porquet et al. (2013) study concentrated on resveratrol inhibition of tau kinases (CDK5, GSK3β, JNK) [147,182]. This paradox suggests that the precise processes of action of resveratrol in tau disease remain unknown.

Resveratrol seems to have neuroprotective benefits connected to SIRT1 activation, according to many research. Some research, nevertheless, also points to resveratrol’s SIRT1-independent properties. Resveratrol, according to Price et al.’s 2012 study, activates SIRT1 and sets AMPK activity, so enhancing mitochondrial health. This work argues that SIRT1 largely determines the neuroprotective effects of resveratrol [156]. Resveratrol’s effects were observed to be restricted in SIRT1 knockout mice, therefore stressing the critical function of SIRT1. Other studies, notably those by Schweiger et al. (2017), have concentrated on the effects of resveratrol apart from SIRT1. This work showed that independent of SIRT1 activation, resveratrol might influence tau dephosphorylation by the MID1-α4 complex. Based on this information, resveratrol might have neuroprotective properties apart from SIRT1 [182].

Moreover, the quantities and effects of resveratrol used in different investigations differ greatly. While some research claims that low dosages of resveratrol (e.g., 25–30 μM) have neuroprotective effects, others contend that higher concentrations are better. For instance, whilst Price et al. (2012) claimed that higher concentrations (25 mg/kg and 215 mg/kg) were unsuccessful or limited, Sun et al. (2017) found that a 40 mg/kg dosage of resveratrol lowered tau phosphorylation and stopped neuroinflammation [156,183]. This implies that the type of the effective dose could affect the dose-dependent effects of resveratrol, hence changing their nature.

The contradicting research results show that numerous unanswered concerns surround the precise mechanism behind the neuroprotective properties of resveratrol. Resveratrol’s effects on Aβ and tau pathologies, variables including SIRT1 activity and dosage dependency, may vary depending on different experimental conditions. Therefore, the focus of more long-term clinical studies and research has to be on more comprehensive mechanisms. Considering elements such as dosage, mode of administration, length of therapy, and personal biological reactions helps one to fully understand the neuroprotective features of resveratrol.

#### 7.1.2. Piceatannol (Astringenin)

Piceatannol (trans-3,4,3′,5′-tetrahydroxystilbene) (Figure 3) gained attention with its structure similarity to resveratrol. It is found in a variety of fruit and herbal plants, including *Rheum officinale*, *R. palmatum*, *Rhodomyrtus tomentosa*, *Passiflora edulis*, *Vitis vinifera*, *V. amurensis,* and *V. thunbergi*. Piceatannol has four hydroxyl groups and one more hydroxyl group compared to resveratrol [186].

In a study in which Aβ_25-35_ (25 µM)-induced PC12 cells were exposed to resveratrol and piceatannol at concentrations of 10 and 20 µM, it was found that piceatannol was more strongly protective than resveratrol. Reduction in ROS formation and inhibition of PARP (poly (ADP-ribose) polymerase) cleavage have been implicated as responsible mechanisms. The strong activity of piceatannol against Aβ-induced toxicity may be due to an additional aromatic hydroxyl group between it and resveratrol [187]. To elucidate the effects of stilbene secondary metabolites on molecules related to APP processing in Neuro2a neuroblastoma cells, resveratrol, oxyresveratrol, and piceatannol were compared. In light of the results, the compounds that prevent sAPP secretion were determined as oxyresveratrol at 40-80 µM concentrations and piceatannol at 10–20 µM concentrations. 5–20 μM resveratrol, 40–80 μM oxyresveratrol, and 10–20 μM piceatannol reduced γ-Secretase activity at higher concentrations. What makes piceatannol different is that at low concentrations in the range of 2.5–10 µM, it increases α-secretase activity, which may be associated with a decrease in Aβ levels without triggering cell death. These secondary metabolites activated the Aβ carboxypeptidases cathepsin B and MMP-9 (matrix metalloproteinase-9) at particular concentrations but inhibited the Aβ endopeptidases NEP and IDE at nearly all concentrations examined. In the Aβ quantification experiment in human Aβ_1-40_ and Aβ_1-42_ secreting HEK293 APPsw (human embryonic kidney 293) cells, it was found that piceatannol reduced the amounts of Aβ_1-40_ and Aβ_1-42_ without causing cell death, in contrast to resveratrol and oxyresveratrol, which made it stand out [188]. Furthermore, in E11 cells generated from human rheumatoid arthritic synovial fibroblasts, piceatannol was demonstrated to control MMP-9 expression [18].

Kawakami et al. conducted a study to examine the effects of piceatannol and resveratrol on SIRT1 protein levels in THP-1 (human leukemia monocytic cell line) cells. SIRT1 protein expression rose in a concentration-dependent manner following a 24 h treatment with piceatannol. In particular, after receiving 6 μM and 10 μM piceatannol, respectively, SIRT1 levels increased by 2.0 and 2.5 times, respectively. Comparably, resveratrol similarly increased SIRT1 expression in a concentration-dependent manner, with a 1.8-fold rise at 3 μM and a 2.0-fold increase at 6 μM. In the same study metabolites of piceatannol and resveratrol (all metabolites at 6 μM dose); isorhapontigenin and rhapontigenin and resveratrol-3-*O*-glucuronide, resveratrol-4′-*O*-glucuronide, and resveratrol-3-*O*-sulfate also examined for their effects on SIRT1 expression. Increased SIRT1 mRNA expression in THP-1 cells occurred after 15 h of stimulation with isorhapontigenin or rhapontigenin. Conversely, glucuronide and sulfate metabolites of resveratrol did not affect SIRT1 mRNA expression [189].

In a more recent study, the possible effect of piceatannol on the “mitochondrial biogenesis” signaling pathway was evaluated through impairments in animal models of stress with the use of reserpine or gamma-irradiation. Rats were administered piceatannol orally (10 mg/kg BW/day) for one week following an acute dose of γ-radiation (6 Gy) or a single subcutaneous injection of reserpine at 1 g/kg BW. The pathways associated with the reduction in oxidative stress, inflammation, and apoptotic responses in the piceatannol treatment group are increased expression of SIRT1/p38-AMPK, PGC-1α signaling pathway, and tissue protein content (improvement in mitochondrial biogenesis) [16].

According to Wang et al., piceatannol protected hippocampal neurons against injury by activating the Sirt1/FoxO1 pathway. In the mouse model of cerebral ischemia/reperfusion injury (CIRI), oral administration of piceatannol (10 mg/kg/day or 20 mg/kg/day) was given to 8-week-old C57BL/6 mice one hour after CIRI and once a day for the following six days. Experimental data showed that treatment after piceatannol reduced hippocampal neuronal pathology and improved neurological functions. Furthermore, there was a decrease in intracellular ROS levels as well as the expression levels of Bax and caspase-3 [190].

Improvement in mitochondrial content and function was observed with piceatannol (5–20 μM) treatment in H_2_O_2_-induced PC-12 (pheochromocytoma-12) cells. These effects were noted as increased expression of mitochondrial transcription factor (TFAM), peroxisome-proliferator-activated receptor-γ coactivator-1a (PGC-1a), and mitochondrial Complex IV. Another important finding from the study is that the SIRT3 protein level, which decreased in cells with exposure to H_2_O_2_, improved positively after piceatannol treatment [191]. Similarly, piceatannol applied to Neuro2a cells exposed to high glucose reduced ROS levels and mitochondrial superoxide production and stabilized mitochondrial membrane potential at concentrations of 5 and 10 μM [192].

Numerous studies conducted on SIRT 1, it has been determined that piceatannol and resveratrol stilbenes are effective in experimental models. In studies comparing both piceatannol performs better than resveratrol. Hosoda et al. emphasized piceatannol in their study as follows: The increase in mitochondrial ROS level caused by antimycin A was reduced by 10 μM piceatannol, and this decrease was greater than that of resveratrol. Piceatannol inhibited antimycin A-induced apoptosis more strongly than resveratrol, and furthermore, the antiapoptotic activity of resveratrol was abolished by inactivation of SIRT1, whereas the antiapoptotic activity of piceatannol was only partially prevented [193].

Riviere et al., in their study with a series of stilbenes in 2007, stated that the most effective ones on Aβ fibrils were resveratrol and piceatannol. Resveratrol, piceid, resveratrol diglucoside, piceatannol, astringine, and viniferin were studied at 10 µM concentration to evaluate against anti-amyloidogenic curcumin. While resveratrol and piceatannol were determined to be approximately twice as active as curcumin at the same concentration, the activity of the other compounds was found to be lower than curcumin [194].

In a study conducted with piceatannol, gnetol, rhapontigenin, and isorhapontigenin, their effects on radical scavenging, AChE inhibition, and Aβ peptide aggregation, as well as their effects on learning in scopolamine-induced amnesiac ICR mice with cognitive dysfunction, were examined. With half-maximal inhibitions of 40.2, 271.74, and 0.48 μM, piceatannol was shown to be the most active in DPPH**^.^** radical scavenging, inhibitory actions against AChE, and Aβ peptide aggregations. In scopolamine-induced mice, it was determined that the stilbene that had the strongest effect on learning was piceatannol (50 mg/kg) [186]. The IC_50_ value of piceatannol obtained from *Passiflora edulis* by Dos Santos et al., which inhibits the AChE enzyme, was found to be 29.420 μg/mL [195].

Piceatannol at 1 μM concentration exhibited its effect in ndSH-SY5Y (human neuroblastoma cell line) cells by suppressing Aβ-induced neurite disintegration and neuronal cell death [196]. Previous studies have similarly shown that piceatannol (25 μM) has a neuroprotective effect against Aβ-induced neurotoxicity in PC12 (rat pheochromocytoma) cells [197]. Wen et al. (2018) examined the antioxidant and neuroprotective effects of trans-4-hydroxystilbene, resveratrol and piceatannol stilbenoids via the PI3K/Akt signaling pathway in rat primary cortical neurons [198]. In the research, experiments focused on the ability of these compounds to scavenge free radicals, reduce intracellular ROS levels, and support cell survival in neurons exposed to Aβ_25-35_-induced neurotoxicity. The mechanisms underlying the neuroprotective effects of stilbenoids were examined through their interaction with the PI3K/Akt pathway to alleviate the damage caused by Aβ. It has been determined that the PI3K/Akt signaling pathway mediates the neuroprotective effects of stilbenoids in rat primary cortex neurons. Promoted Akt phosphorylation by stilbenoids inhibited cell apoptosis and promoted cell survival. Furthermore, additional evidence for neuroprotective effects is the inhibition of expression of Bcl-2/Bax proteins and caspase cleavage. The interaction of stilbenoids with this pathway caused a decrease in intracellular ROS levels and therefore a decrease in oxidative stress, while also preventing neuronal damage caused by Aβ_25-35_ application [198]. Likewise, the underlying mechanism of piceatannol-3′-O-β-D-glucopyranoside (5 and 50 μM) in preventing colistin-induced neurotoxicity in N2a cells has been associated with suppressing ROS levels, enhancing the activities of GSH, CAT, and SOD, activating the NRF2/HO-1 pathway and thus preventing the apoptosis of nerve cells [199]. The studies analyzed regarding piceatannol and their key findings are summarized in Table 2.

It is noted that the effects of piceatannol in several cell models and experimental settings concentrate on diverse directions. Apart from its neuroprotective and antioxidant properties, studies reveal that piceatannol is efficient by focusing on several metabolic pathways. Sometimes, nevertheless, there are contradicting findings between these impacts. varied investigations yield varied effects of piceatannol on SIRT1. Piceatannol raised SIRT1 levels, according to one study, and this was linked to favorable effects on anti-apoptotic and mitochondrial biogenesis at the cellular level [189,190]. Notwithstanding these findings, another investigation found that piceatannol either demonstrated reduced efficacy relative to resveratrol or compromised the function of SIRT1. Specifically, studies show that whilst piceatannol has a more significant impact but this effect is not dependent on SIRT1, SIRT1 guards against oxidative stress and death generated by antimycin A. These contradicting results could suggest that piceatannol might affect SIRT1 in distinct cellular settings and pathways differently. Depending on the dose and cellular environment, piceatannol is probably going to cause either raising or decreasing SIRT1 activity. Different research has explored the impact of piceatannol on Aβ aggregation in various approaches. While another study found piceatannol had a modest ability to suppress Aβ, some claimed it stopped or removed Aβ aggregation. This discrepancy could imply that variations in concentration, timing, compound formulation, or model systems applied in the investigations could be the causes of the action of piceatannol. Studies on Piceatannol have shown conflicting findings, hence additional experimental data are required to grasp the effects of the chemical in a larger perspective.

#### 7.1.3. Oxyresveratrol

Oxyresveratrol (2,3′,4,5′-tetrahydroxystilbene) (Figure 3), a natural stilbene, has recently attracted attention due to its simple chemical structure and various possible medical applications [200]. The fact that neuroinflammation is one of the key parameters in the emergence and development of AD and that oxyresveratrol has anti-inflammatory potential has inspired the studies. Hankittichai et al. described that oxyresveratrol (10, 20, and 40 µM) inhibited the activation of the PI3K/AKT/p70S6K pathway in IL-1β-induced HMC3 (human microglia cell) cells and significantly reduced the triggered IL-6 and MCP-1 release [201]. An alternative study results also revealed that oxyresveratrol (10 μM) acts through MAPKs and NF-κB signaling pathways to suppress inflammation by preventing the synthesis of pro-inflammatory mediators in lipopolysaccharide (LPS)-induced BV-2 cells [190].

The effectiveness of oxyresveratrol in primary rat cortical neurons induced with 10 µM Aβ (25–35) was studied at 1 and 10 µM concentrations. The subsequent findings demonstrated that oxyresveratrol inhibits increased [Ca^2+^]_c_, glutamate release, and ROS production while simultaneously averting apoptosis [202]. In a different investigation, Andrabi et al. used a transient mouse middle cerebral artery occlusion (MCAO) paradigm to show the neuroprotective effects of oxyresveratrol. Oxyresveratrol improved neurological impairments brought on by I/R damage, dramatically decreased the brain infarct volume of MCAO rats, and blocked both caspase-3 activation and cytochrome c release in MCAO rats at different doses of 10 or 20 mg/kg [203].

In a study examining the neuroprotective effects of oxyresveratrol (10 and 20 µM) in rotenone-induced SH-SY-5Y cell line, it was determined that oxyresveratrol increased cell viability and GSH amount, decreased MMP and ROS levels, Bax and cytochrome C protein expression and caspase-3 activity [204].

In the study examining the effects of trans/cis-resveratrol mixture, oxyresveratrol, veraphenol, and cis-scirpusin A, stilbenes, on BACE1 inhibition, IC_50_ values were found to be 1.5 × 10^5^, 7.6 × 10^6^, 4.2 × 10^6^, and 1.0 × 10^5^ M, respectively. The stilbenes appeared to be rather specific BACE1 inhibitors because they showed reduced inhibition of a-secretase (TACE) and other serine proteases such as chymotrypsin, trypsin, and elastase [171].

Previous research suggests that astrocytes may directly play a role in amyloid plaque development in the adult mouse astrocyte model [205,206]. For this reason, the efficacy of oxyresveratrol to decrease APP formation in a mouse astrocyte model produced by glucocorticoid corticosterone and glucocorticoid dexamethasone was studied. The co-administration of glucocorticoid corticosterone and oxyresveratrol (10 µM) resulted in a substantial reduction in APP synthesis, but oxyresveratrol treatment alone did not. Moreover, LC3 (microtubule-associated protein light chain 3) synthesis was markedly elevated in glucocorticoid corticosterone and oxyresveratrol co-treated cells, indicating that oxyresveratrol inhibited astrocyte autophagy [17].

Hasriadi et al. investigated the potential neuroprotective effects of *Artocarpus lakoocha* extract and oxyresveratrol against toxicity induced by hydrogen peroxide in SH-SY5Y cells. The findings suggest that both *Artocarpus lakoocha* extract and oxyresveratrol (5–100 μM) exhibit a neuroprotective effect by mitigating oxidative stress-related damage (removing ROS and preventing lipid peroxidation) to neurons, which is significant in the context of neurodegenerative disorders [207]. In vitro and in vivo studies are summarized in Table 3.

The prospective neuroprotective effects of oxyresveratrol are mostly due to its anti-inflammatory, antioxidant, and apoptosis-inhibiting capabilities. The effects of oxyresveratrol resemble those of resveratrol; however, it is noted to possess a more potent antioxidant impact and the ability to suppress Aβ aggregation. Divergent findings point to different effects of oxyresveratrol depending on dosage, context, and cell type. Furthermore, many oxyresveratrol mechanisms—including BACE1 inhibition and autophagy inhibition—have variable effects depending on the study. This implies that the effects of the molecule depend more on certain conditions and need assessment over a larger spectrum of possibilities.

#### 7.1.4. Pterostilbene

Pterostilbene (trans-3,5-dimethoxy-4-hydroxystilbene) (Figure 3) has been shown to act by different mechanisms in certain animal models of AD and cell culture models of neurotoxicity. Pterostilbene protects neuroblastoma cells from high glucose-induced damage by reducing ROS formation in a dose-dependent manner, delaying cell death, and enhancing the activities of mitochondrial complexes I and III, mitochondrial cytochrome C, and mitochondrial membrane potential. Furthermore, treatment with pterostilbene increased the amount of Nrf2, HO-1, and glutathione S-transferase (GST), suggesting protection against neuronal oxidative stress [208]. Additionally, Wang et al. demonstrated that pterostilbene reduced glutamate-induced oxidative stress in neurons via activating Nrf2 signaling [209].

In a study investigating the neuroprotective effects of pinosylvin, pterostilbene, pinostilbene, and 4-methoxy-trans-stilbene, H_2_O_2_ was first used as an inducer in PC12 cells. In these preliminary studies, the strong protective effects of stilbenes at a dose of 10 μM were determined and detailed examination was carried out in the OGD/R (oxygen and glucose deprivation/reperfusion) model. Among the stilbene derivatives studied, pinosylvin was found to be the most effective compound that reduces cell death. The underlying mechanisms include raising the ratio of BCL-2 to Bax protein expression, partially regaining mitochondrial function, and reducing the release of cytochrome C from mitochondria, reducing the generation of ROS, and activating intracellular antioxidant enzymes. Subsequent investigations revealed that pinosylvin triggered the Nrf2 pathway and PINK1/Parkin-related protective mitophagy, as evidenced by the increased protein levels of LC3 II, Beclin1, PINK1, and Parkin, as well as the translocation of Nrf2 to the nucleus [210]. In a different study, it was stated that pterostilbene (25 and 50 μM) increased the viability of the cells while reducing the level of apoptosis and intracellular ROS activity in PC12 cells induced by Aβ_25-35_. The key finding has been that the anti-apoptosis mechanism is associated with activation of the PI3K/Akt signaling pathway, which promotes Akt phosphorylation at Ser-473 [197].

Application of pterostilbene at 5 and 10 μM concentrations to BV-2 cells exposed to Aβ_1−42_-induced cytotoxicity showed a suppressive effect on NO synthesis as well as iNOS mRNA and protein expression. Furthermore, it inhibited the Aβ_1−42_-activated NLRP3/caspase-1 inflammasome, reduced TNF-α, IL-1β, and IL-6 expression and secretion levels, and lessened Aβ_1−42_-induced cytotoxicity in microglia. These findings suggest that PTS could be a useful treatment for AD by preventing hyper-activated microglia [13].

SAMP8 mice were used in the study by Chang et al. comparing resveratrol and pterostilbene. For eight weeks, either pterostilbene or resveratrol at the same dose (120 mg/kg of diet) or a control diet was given to five-month-old SAMP8 mice. Pterostilbene restored cognitive performance to SAMR1 control levels in the Radial Arm Water Maze Test. It is interesting to note that the resveratrol-fed group learned more than non-fed SAMP8 animals, but this difference was not statistically noteworthy. Similarly, while pterostilbene was found to be effective against Tau phosphorylation, resveratrol was determined to be ineffective. Interestingly, it was shown that SIRT1 expression did not differ significantly between groups. Acetylation of p53, which is known to be inhibited by SIRT1, was also measured and it was concluded that acetylated p53 did not change significantly with either stilbene derivative treatment [211]. The effect of pterostilbene on cognitive function was later also proven by Naik et al. Pterostilbene treatment (10, 30, and 50 mg/kg, orally) alleviated the streptozotocin-induced memory loss in rats. It has also been stated that pterostilbene improves cholinergic transmission by inhibiting cholinesterases (ChEs). Overall, brain antioxidant measures improved, including increased catalase and superoxide dismutase activity, greater GSH levels, decreased nitrite levels, lipid peroxides, and carbonylated proteins [212]. Similarly, in streptozocin-induced rats, Li et al. stated that pterostilbene administered at 20 mg/kg/day for 5 weeks alleviated body weight loss and memory impairment by regulating monoamine oxydase B (MAO-B). Other findings of the study are that it alleviates Aβ_1−42_ accumulation and tau hyperphosphorylation by downregulating MAO-B expression, inhibition of streptozocin-induced neuronal apoptosis, increase in SOD and GSH levels, decrease in pro-inflammatory cytokines TNF-α, IL-1β, and IL-6, as well as neuroinflammatory effects [213].

Pterostilbene has been proven to mitigate neuron loss and ROS buildup in the brain of mice treated with Aβ_1-42_–induced cognitive impairment. In addition, 10, 20, and 40 mg/kg oral pterostilbene treatment groups showed increased transcription and expression of antioxidant genes like heme oxygenase-1 and superoxide dismutase, as well as promoted nuclear factor-E2 p45-related factor 2 (Nrf2) nuclear translocations [214].

In two different studies, the effects of pterostilbene on cognitive ability were examined using different tests. The first study found that the detrimental effects of aging on cognitive performance, particularly working memory, were reversed in the treated groups in a dose-dependent manner when pterostilbene doses of 40 mg/kg diet, 0.004% *w*/*w*, and 160 mg/kg diet were applied [215]. In the second trial, aged rats given a diet containing 22.5 mg/kg of pterostilbene daily showed improvements in behavioral testing and memory consolidation. Additionally, it has been reported that the dentate gyrus contains higher levels of REST, PSD-95, and mitochondrial porin1 and that the T-maze test score and the phosphorylation levels of cAMP-sensitive element binding protein (CREB) are positively correlated [216].

The pterostilbene-induced activation of SIRT1 increased Nrf2 expression, increased SOD levels, inhibited the Aβ_25-35_-induced mitochondrial death pathway, improved neural plasticity, and slowed down neuronal loss. In isolated mouse neurons, administration of the SIRT1 inhibitor EX527 resulted in a decrease in mitochondrial membrane potential and a reduction in the protective effects against Aβ_25-35_-induced damage. In the in vivo part of the same study, it was clarified that intragastric administration of 40 mg/kg pterostilbene showed effective neuroprotection on Aβ_25-35_-induced cognitive dysfunction in the novel object test, Y-maze test, and Morris water maze tests. In light of these findings, it is possible that pterostilbene inhibits the mitochondrial apoptotic pathway via SIRT1/Nrf2-mediated antioxidant processes, potentially improving learning, memory, and neuroplasticity in AD rats [217]. Table 4 lists the mechanisms via which pterostilbene has demonstrated its potential in preclinical studies on AD.

Pterostilbene is an effective antioxidant that reduces the production of ROS, which in turn protects cells from harm. It may also mitigate neuroinflammation through its anti-inflammatory effects, impede the accumulation of Aβ, and decelerate the progression of AD by diminishing neuroinflammation. Research on the neuroprotective benefits of pterostilbene predominantly yields favorable outcomes; nonetheless, discrepancies exist among the findings. These contradictions may fluctuate based on the cell types employed, doses, variations in experimental paradigms, and the heterogeneity of the mechanisms of action. Some research unequivocally illustrates the anti-inflammatory and anti-apoptotic properties of pterostilbene; however, in other studies, these effects are less definitive. The activation of SIRT1 is a significant mechanism of pterostilbene, which has been shown to positively influence Nrf2 activation, elevate SOD levels, maintain mitochondrial membrane potential, and enhance neuroplasticity [217]. A study on SIRT1 inhibition of p53 acetylation revealed that pterostilbene therapy did not produce substantial alterations in p53 acetylation [211]. This study indicates that pterostilbene does not uniformly influence SIRT1, and its effects may alter based on varying dosages or situations.

#### 7.1.5. Gnetol

Although there have been few investigations on gnetol (2,3′,5′,6-tetrahydroxy-trans-stilbene) (Figure 3) and AD, its partial effects have been investigated in two separate studies. According to one report, gnetol reversibly and competitively inhibits BChE, which may be useful in the treatment of AD [218]. In the other study, the protective effects on N2a cells induced by malathion, a neurotoxic organophosphate, were examined. By controlling the expression of vascular endothelial growth factor (VEGF) and heart fatty acid binding protein 3 (hFABP3), at 5, 10, and 20 µM concentrated genetol prevented cells from progressing into apoptosis. This improved the cellular and nuclear morphological alterations brought on by malathion. Additionally, gnetol administration increased the level of NGF and neurite outgrowth [219].

#### 7.1.6. Amurensin G

Amurensin G (C_42_H_32_O_9_) (Figure 6) is a trimer of resveratrol found in *Vitis amurensis* Rupr. (Vitaceae) [220]. In an in vivo study with *V. amurensis* (50 and 100 mg/kg, p.o.), the plant effectively protected mice against Aβ_25-35_-induced memory deficit and histological examinations showed that *V. amurensis* treatment prevented Aβ_25-35_-induced neuronal death in the hippocampus. In AD, Aβ accumulation is accompanied by an increase in ChE activity [221]. To slow disease progression and improve cognitive function, it is recommended to inhibit the breakdown of acetylcholine by ChE, thereby restoring cholinergic balance [222,223].

*V. amurensis* inhibited Aβ_25-35_-induced [Ca^2+^]_i_ increase, ROS generation and neuronal cell death in cultured cortical neurons. *V. amurensis* prevents Ca^2+^ entry through voltage-dependent calcium channels (VDCC) and/or N-methyl-D-aspartic acid (NMDA) receptor-coupled channels, but the mechanism by which *V. amurensis* blocks these channels is unclear [224].

The presence of antioxidant species in *V. amurensis* suggests that it inhibits Aβ_25-35_-induced neuronal death through ROS scavenging activity. In addition, resveratrol derivatives have also been shown to inhibit Ca^2+^ entry through Ca^2+^ channels [225], suggesting the possibility that these compounds may inhibit Aβ_25-35_-induced neuronal death by contributing to the inhibitory activity of *V. amurensis* on Ca^2+^ entry. The resveratrol derivatives amurensin G, r-2-viniferin and trans-ε-viniferin isolated from *V. amurensis* were able to inhibit Aβ_25-35_-induced [Ca^2+^]_i_ increases, ROS formation and ultimately apoptotic neuronal death. Studies have been shown that *V. amurensis* can inhibit caspase activity to reduce Aβ_25-35_-induced neuronal apoptosis [202,226]. Aβ_25-35_ injected intracerebroventricularly into rodents has been shown to cause memory impairment [221,227].

High levels of Aβ may lead to the generation of a number of ROS (O^2−^ and NO) and, in a rapid interaction, ONOO^−^ production, thus inducing oxidative stress. All this can cause the damage seen in AD [228,229]. An ONOO^−^ scavenger protects against Aβ_25-35_-induced memory impairment [229], while antioxidants such as α-tocopherol protect against in vitro cytotoxicity and Aβ-induced learning and memory deficits [230,231]. In cultured neurons treated with 10 μM Aβ_25-35_, ROS levels were significantly increased and this effect was inhibited by *V. amurensis* [224]. The antioxidant components present in the leaves and stem of *V. amurensis* have been attributed to the inhibition of ROS formation [232]. The ChE inhibitor donepezil has also been reported to contribute to neuroprotective activity in AD through its blocking effect on VDCC [233].

#### 7.1.7. Miyabenol C

Miyabenol C (C_42_H_32_O_9_) (Figure 6) is found in *Vitis* species (Vitaceae) [234], *Carex* species (Cyperaceae) [23], *Caragana* species (Fabaceae) [235], *Parthenocissus quinquefolia* L. (Vitaceae) [236] and *Sophora davidii* Franch. (Fabaceae) [237]. It is also a resveratrol trimer in the stem and leaf extract of *Vitis thunbergii* var. *taiwaniana* F. Y. Lu [238] (VTT) (Vitaceae), a wild grape native to Taiwan. It has been shown to be a significant β-secretase inhibitor and may reduce Aβ levels in vitro/in vivo. Aβ is a proteolytic product of APP and is produced by sequential cleavages by enzymes called β- and γ-secretases [239,240].

The transmembranous aspartic protease β-site APP cleaving enzyme 1 (BACE1) is known to be the main β-secretase in vivo [241,242]. BACE1 levels and activity have been found to be high in postmortem brains of sporadic AD patients [19,243,244], suggesting a role for BACE1 in AD. Therefore, moderate inhibition of β secretase activity has been considered promising for AD intervention. Since APP cleavage by γ-secretase is the final step for Aβ production, it was possible that miyabenol C inhibited γ-secretase activity and reduced Aβ production. In addition, treatment with miyabenol C did not affect the protein levels of PS1, a major γ-secretase component. These results demonstrated that miyabenol C does not inhibit γ-secretase activity. Furthermore, miyabenol C treatment did not affect the protein levels of APP, the major β-secretase BACE1 and the two major α-secretases ADAM10 and ADAM17 [245,246].

These results showed that miyabenol C did not reduce APP amyloidogenic processing by affecting α- and β-secretase protein levels. Since miyabenol C inhibited β-processing of APP without affecting BACE1 levels, it was considered to directly inhibit BACE1 activity. To explore this possibility, a cell-based assay was performed to measure β-secretase activity. Since there is another possibility that miyabenol C inhibits APP β-processing and Aβ formation by promoting α-secretase activity, it is used myabenol C-treated SY5Y cells (Cells are derived from neuroblastoma cell line. It is a model for neurodegenerative disorders as cells can be transformed into various functional neuron types by the addition of specific compounds.) TACE activity, a major α-secretase, was tested and it was found that myabenol C did not affect TACE activity. This suggests that miyabenol C does not affect α-secretase activity. In the light of the results obtained, it was shown that miyabenol C is an important β-secretase inhibitor and can reduce Aβ formation both in vitro and in vivo [247].

#### 7.1.8. Mulberroside A

Mulberroside A (2′,3,4′,5-tetrahydroxystilbene-3, 4′-β-D-glucoside) (Figure 3) is a natural polyhydroxylated stilbene compound found in high amounts in the roots and branches of *Morus alba* L. (Moraceae). It has nephroprotective, hypoglycemic and antidiabetic effects. Its metabolite oxyresveratrol has anti-inflammatory and neuroprotective effects. It has been suggested that Mulberroside A may be used in the treatment of brain ischemic injury and may elicit neuroprotective effects [28].

#### 7.1.9. Polydatin

Polydatin, also called piceid (3,4′,5- trihydroxystilbene-3-β-D-glucoside) (Figure 3) is a compound isolated from *Polygonum cuspidatum* Sieb. et Zucc. (Polygonaceae). It is also found in grapes, peanuts, hop cones and pellets, red wine, cocoa-containing products, chocolate products and in many daily diets [248].

Although the bioavailability of resveratrol, which has neuroprotective effects, is low, the bioavailability of polydatin, a resveratrol glucoside, is higher [249].

Most breast cancer patients receiving chemotherapy suffer from severe cognitive impairments called “chemobrain”. In a study, it was shown that polydatin treatment significantly protected against DOX-induced learning and memory impairment, suppressed DOX-induced oxidative stress by upregulating Nrf2, inhibited the inflammatory response by activating the NF-κB pathway, and reduced hippocampal apoptosis in a chemobrain model induced by intraperitoneal doxorubicin (DOX, 2 mg/kg) injection to rats once a week for 4 weeks. It was thought that polydatin may help prevent chemobrain after chemotherapy in cancer patients [250].

Polydatin has been shown to protect against H_2_O_2_-induced mitochondrial dysfunction and neurotoxicity and Ngb may play a key role in this regard. The antioxidative and neuroprotective role of polydatin was also reported to be mediated by activation of CREB/Ngb signaling. The results suggest that polydatin may be involved in the treatment of various oxidative stress-related neurological disorders with its strong neuroprotective effect [251].

Polydatin has been shown to prevent Aβ aggregation by inhibiting Aβ_25-35_ polymerization in vitro and mediate up-regulation of acetylcholine receptors by preventing Aβ_1-42_ induced α3 and α7 acetylcholine receptor number reduction. The increase in acetylcholine receptors suggests that there will be more acetylcholine binding to the receptor and that polydatin may be used against AD [252].

#### 7.1.10. Rhapontigenin

Rhaponticin (3,3′,5-trihydroxy-4′-methoxystilbene 3-*O*-D-glucoside) (Figure 3), whose aglycone is rapontigenin (3,3′,5-trihydroxy-4′-methoxystilbene), is a stilbene glucoside derived from rhubarb roots (*Rhei rhizoma*). In a study evaluating the protective role of rhapontigenin against Aβ_1-42_-induced toxicity, it was found to significantly preserve the viability of neuroblastoma cells (IMR-32) in a dose-dependent manner and to exert a protective effect on mitochondrial functionality. In the presence of rhaponticin and rhapontigenin, three major apoptosis-associated genes (Bax, Bcl-2 and mcl-1) were highlighted in Aβ-treated cells. When Aβ-treated IMR-32 cells were incubated in the presence of rhaponticin and rhapontigenin, Bax up-regulation was attenuated compared to that shown by cells treated with Aβ peptide alone. Up-regulation of bax causes the release of apoptosis-promoting factors into the cytoplasm. Therefore, the attenuation of Bax up-regulation is associated with the protective efficacy of rhaponticin and rhapontigenin. When Aβ-treated IMR-32 cells were incubated in the presence of rhaponticin and rhapontigenin, Bcl-2, an anti-apoptotic gene, was down-regulated compared to cells treated with Aβ peptide alone. Thus, stilbenes have been shown to have a protective role on Aβ-induced apoptosis as well as being potential proapoptotic agents [181].

Since the mechanism of Aβ-mediated toxicity appears to be mediated by an apoptotic pathway involving mitochondria and the Bcl-2 protein family [253], it is hypothesized that rhaponticin and rhapontigenin exert their protective role by interfering with some Aβ-mediated-apoptotic events. Rhaponticin and rhapontigenin were found to be able to alleviate the toxicity exerted by Aβ on mitochondrial respiration. Hydrogen peroxide and/or free radicals within mitochondria are largely thought to be the main inducers of Aβ-mediated apoptosis [254]. Therefore, the protective effect of rhaponticin and rhapontigenin at the mitochondrial level may be related to their antioxidant properties [255]. It should also be underlined that the antiapoptotic effect shown by rhapontigenin is stronger compared to that shown by rhaponticin. However, given that rhaponticin can be administered orally and that rhapontigenin is also metabolized by the human intestinal flora, rhaponticin may be considered an effective prodrug [256].

#### 7.1.11. Scirpusin A, B

The 70% acetone fraction of *Passiflora edulis* Sims (Passifloraceae) seeds is rich in polyphenolic stilbenes including trans-piceatannol, trans-resveratrol, scirpusin A and B (Figure 6) and cassigarol E. In silico analyses showed that trans-piceatannol and trans-resveratrol selectively inhibit AChE. In particular, two stilbene dimers, cassigarol E and scirpusin A, were found to potentially inhibit both AChE and BChE at nanomolar levels even lower than positive controls (donepezil and tacrine) [257].

Scirpusin A and ε-viniferin glucoside were reported to strongly inhibit Aβ fibril formation and have a very low IC_50_ value (≤1 μM). These two stilbene dimers with low IC_50_ values are effective in the inhibition of Aβ fibril formation even at low doses. Therefore, it is thought that they may have an important role in AD treatment [258].

#### 7.1.12. ε-Viniferin

ε-viniferin (C_28_H_22_O_6_) (Figure 6) is a phenolic stilbene derivative with resveratrol dimer. It is found in *Bombax malabarica* DC (Bombacaceae), *Cayratia trifolia* L. (Vitaceae), *Dryobalanops lanceolata* Burck (Dipterocarpaceae), *Vitis rotundifolia* Michx. (Vitaceae), *Vitis thunbergii* Siebold. & Zucc. (Vitaceae), *Vitis vinifera* L. (Vitaceae), *Hopea parviflora* Bedd. (Dipterocarpaceae)*, Rheum undulatum* L. (Polygonaceae), *Parthenocissus quinquefolia* L. (Vitaceae)*, Rheum lhasaense* A. J. Li et P. K. Hsiao (Polygonaceae)*, Gnetum microcarpum* Blume (Gnetaceae) [259].

Trans-ε-viniferin glucoside was reported to exhibit the strongest inhibition of Aβ_25-35_ aggregation in vitro [258,260]. In a similar study, trans ε-viniferin inhibited the most toxic forms of Aβ_40_ and especially Aβ_42_ peptides. The inhibition of Aβ_40_ and Aβ_42_ peptides by trans-ε-viniferin is due to the inhibition of fibril formation of these peptides in vitro. In conjunction with the inhibition of fibrillization, these peptides have been shown to have a strong protective effect against PC12 cell death [261]. PC12 cells are often used to study neuronal cell death and neurotoxic injury, are neuroblastic cells, have an embryological origin, and can easily differentiate into neuron-like cells. This differentiation means that they have similar properties to neurons [262].

In a cellular model of AD, trans-ε-viniferin was found to significantly reduce TNFα and IL-6 levels in cells stimulated with LPS or Aβ_42_ [263,264].

Methanol extract prepared from the subsoil parts of *Caragana chamlague* LAMRK (Leguminosae) and (+)-α-viniferin and cobophenol A isolated from it were found to inhibit AChE. (+)-α-viniferin has a bulky structure that masks AChE and prevents acetylcholine from binding to AChE in a non-competitive manner, suggesting that this compound may be an AChE inhibitor. However, cobophenol A (C_56_H_44_O_13_), which is in tetramer form, has a bulky structure but is difficult to access AChE, suggesting that its activity may be reduced [265].

Gnetol, amurensin G, polydatin, scirpusin A/B, ε-viniferin, and miyabenol C, which have limited research on AD, demonstrate diverse therapeutic approaches. Amurensin G and polydatin offer a synergistic effect on neuroprotection and disease modification through mechanisms such as BChE inhibition and antioxidant activity. Miyabenol C and ε-viniferin principally concentrate on regulating Aβ generation or aggregation, directly addressing AD pathogenesis. Amurensin G, polydatin, and scirpusin A/B exhibit extensive multi-target effects, influencing oxidative stress, calcium dysregulation, cholinergic pathways, and Aβ aggregation. Gnetol, scirpusin A, scirpusin B, and ε-viniferin all engage with cholinergic pathways, either by inhibiting BChE or modulating acetylcholine receptors, rendering them particularly pertinent for mitigating cognitive decline in AD. Their potential synergy, if cultivated together, could provide diverse treatment methods for AD.

### 7.2. Limits and Difficulties of Natural Stilbene-Based Therapies

The low bioavailability of stilbene-based compounds, notably resveratrol, is among their most important drawbacks. Rapid metabolism and poor absorption in the gastrointestinal tract can make it difficult for them to achieve effective concentrations in vivo, even if their in vitro and animal model effects seem promising. Being a resveratrol glucoside, polydatin has more bioavailability but still finds difficulty reaching therapeutic levels in the brain.

Although stilbenes have shown neuroprotective properties, in many cases the exact chemical processes are yet unknown. For instance, whereas amurensin G and miyabenol C stop Aβ aggregation, their precise method of action—that of either interacting with certain receptors, enzymes, or pathways—is sometimes not entirely known. Because it is challenging to maximize their use or forecast their effects in clinical settings, this lack of mechanistic clarity can impede the evolution of stilbene-based treatments.

Long-term safety data on stilbene derivatives is lacking; hence, although certain compounds have exhibited protective properties in preclinical tests, their efficacy and safety in people remain under research. For instance, miyabenol C has been found to be a strong β-secretase inhibitor; its effects on other proteolytic mechanisms or off-target effects have not been fully investigated. Furthermore, molecules such as rhapontigenin and scirpusin A, which have been demonstrated to alter death pathways, may have pro-apoptotic effects in some situations and could be detrimental in some cellular conditions.

While in vitro data and preclinical research show promise, clinical evidence supporting the use of stilbene-based medicines for treating neurodegenerative illnesses, including AD, is sorely lacking. While most investigations have been carried out in animal models or cell cultures, there is a dearth of extensive human clinical trials to validate their safety and efficacy over extended terms. Stilbene-based medicines will remain experimental until more strong clinical data are available.

Although stilbene-based drugs show great potential for the treatment of neurodegenerative illnesses, including AD their clinical relevance is still restricted by elements including poor bioavailability, unclear mechanisms of action, and insufficient safety evidence. To completely grasp the therapeutic possibilities of these molecules in human populations and to solve these constraints, more study is required. Though they constitute an interesting field of research right now, they are not without difficulties.

## 8. Synthetic Derivatives of Stilbenes and Their Neuroprotective Activity

A variety of methods may be employed to reduce Aβ_42_-induced neuroinflammation. These include the inhibition of the aggregation of toxic Aβ peptides, the inhibition of the NF-κB pathway and the scavenging of free radicals. It is thought that these methods may play a significant role in the reduction in neurodegeneration associated with AD. Wiciński et al. (2020) proposed that resveratrol derivatives may possess the capacity to diminish the levels of hyperphosphorylated tau protein, which is linked to neurodegeneration, by facilitating tau dephosphorylation [266]. It can be concluded that resveratrol derivatives have the potential to function as multifaceted compounds with neuroprotective properties. It is postulated that they may assist in the protection against oxidative-nitrosative stress, which is hypothesized to occur prior to the pathological changes associated with AD. Furthermore, they may enhance metabolic processes, such as glucose metabolism, which can have a beneficial impact on brain function and protect neurons. The study highlights the importance of these compounds in targeting various pathways that are implicated in neurodegeneration. These compounds are created by linking resveratrol to other pharmacophore molecules, resulting in novel compounds with diverse biological activities. Multi-targeted drug ligands (MTDLs) are currently being developed as potential treatments for neurodegenerative disorders, including AD. These compounds target multiple pathways involved in neuroprotection and have demonstrated promising biological activity in vitro. It has been demonstrated that specific compounds exert dual inhibitory effects on enzymes, including AChE and MAO-B. Examples of such compounds include pyridoxine-resveratrol Mannich base derivatives, tacrine-resveratrol fused hybrids and resveratrol-maltol hybrids. Moreover, these compounds have exhibited antioxidant properties and the capacity to chelate metals, which are vital for the management of oxidative stress and metal-induced neurotoxicity associated with AD [266]

**Figure 9:**
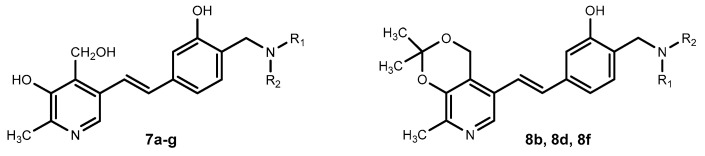
Pyridoxine-Resveratrol hybrids Mannich base derivatives **7a–g**, **8b**, **8d** and **8f**.

According to Yang et al. (2017) [208], the Mannich base derivatives of pyridoxine-resveratrol hybrids were found to have dual inhibitory activity against AChE and MAO-B (Figure 9). The pyridoxine–resveratrol hybrid derivatives **7d** ((E)-5-(3-hydroxy-4-(pyrrolidin-1-ylmethyl)styryl)-4-(hydroxymethyl)-2-methylpyridin-3-ol) and **8b** ((E)-5-(4-(piperidin-1-ylmethyl)-3-hydroxystyryl)-4-(hydroxymethyl)-2-methylpyridin-3-ol) were investigated for their inhibitory effects on AChE and MAO enzymes, in addition to their antioxidant and metal-chelating capacities [208]. Both compounds exhibited significant AChE inhibition, with IC_50_ values of 2.11 μM (**7d**) and 1.56 μM (**8b**), respectively. Notably, compound **7e** demonstrated the most potent MAO-B inhibition within the series (IC_50_ = 2.68 μM). Enzyme kinetics indicated that compound **7d** functions via a mixed-type inhibition mechanism on AChE. Molecular docking studies were conducted to elucidate the binding interactions of the compounds with MAO-B. Furthermore, the antioxidant and metal-chelating activities observed across the series may enhance their therapeutic potential as multifunctional agents in the treatment of AD, through the simultaneous modulation of cholinergic and monoaminergic pathways. The compounds **7d** and **8b** have been demonstrated to possess potent inhibitory properties with regard to AChE. The compound **7d** exhibits binding at both active sites of AChE, manifesting a mixed inhibitory activity. Furthermore, the derivatives exhibit antioxidant and metal chelating properties, which may contribute to the alleviation of oxidative stress and the reduction in metal-induced neurotoxicity associated with AD [208].

Two studies have investigated the potential of resveratrol derivatives in the treatment of AD, with a particular focus on the neuroprotective effects observed. Wiciński et al. (2020) highlight the considerable potential of resveratrol derivatives in reducing Aβ_42_-induced neuroinflammation, impeding the formation of toxic Aβ peptides, inhibiting the NF-κB pathway, and neutralizing free radicals [266]. Moreover, the authors highlight the ability of these compounds to facilitate tau dephosphorylation, reduce oxidative-nitrosative stress, and enhance metabolic functions such as blood sugar regulation. Furthermore, the study highlights the development of MTDLs designed to modulate multiple pathological pathways implicated in AD, including cholinergic dysfunction, oxidative stress, and metal ion dysregulation. Within this context, Yang et al. (2017) investigated a series of pyridoxine–resveratrol hybrid molecules, emphasizing their dual inhibitory activity against AChE and MAO-B, two key enzymes involved in the progression of AD [208]. Among these, compounds **7d** and **8b** demonstrated notable potency, as evidenced by their low IC_50_ values. In addition to their enzyme inhibitory properties, these hybrids exhibited significant antioxidant activity and metal-chelating capacity, supporting their potential as multifunctional therapeutic agents in the management of neurodegenerative disorders. The study offers a comprehensive insight into the mechanisms of enzyme inhibition and the potential of these hybrids as dual inhibitors for the treatment of AD. In conclusion, Wiciński et al. (2020) provide a comprehensive overview of the neuroprotective potential of resveratrol derivatives, while Yang et al. (2017) explore the specific enzyme inhibitory mechanisms of pyridoxine-resveratrol hybrids in depth [208,266]. Both studies highlight the potential of the compounds as a basis for developing new treatments for AD. The antioxidant properties of pyridoxine-resveratrol hybrids (Mannich base derivatives) have been demonstrated in previous studies. By neutralizing ROS and reducing brain damage, these derivatives may help to combat oxidative stress in AD. The ability of the compounds to chelate metals, including copper, iron, and zinc, has also been discussed. By preventing the excess of metal ions that contribute to the formation of amyloid plaques and NFTs, these compounds may be therapeutically useful in the treatment of AD by reducing the neurotoxic effects of metals [267,268,269,270].

**Figure 10:**
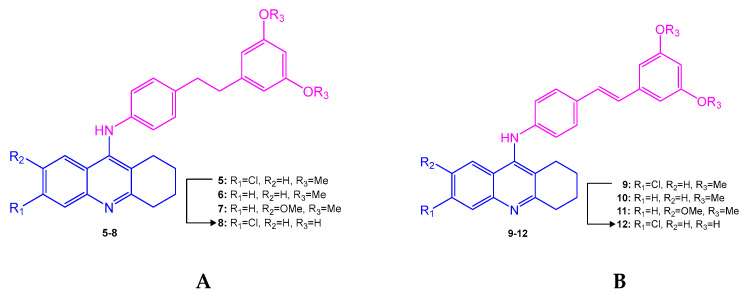
General synthetic schemes for tacrine–resveratrol hybrids: (**A**) compounds **5**–**8**, (**B**) compounds **9**–**12** with structural variations.

Jěrábek et al. (2017) developed MTDLs for AD by synthesizing tacrine-resveratrol hybrid compounds. The authors combined the structure of tacrines with that of resveratrol to design and synthesize tacrine-resveratrol hybrid compounds (**5**–**12**) (Figure 10) [271]. Compounds **5**–**12** are novel hybrid molecules that combine the structures of tacrine and resveratrol. These compounds have the potential to act as multi-targeted ligands in the treatment of AD. The compounds were designed and synthesized with the objective of targeting key proteins involved in the pathogenesis of AD, including AChE and Aβ self-aggregation. The study evaluated the neuroprotective and immunomodulatory properties of these compounds in a range of in vitro models, including AD cell models. The objective of the research was to identify safe and effective therapeutics for the treatment of AD by addressing multiple aspects of its pathogenesis. Compound **5** (6-chloro-N-(4-(3,5-dimethoxyphenethyl)phenyl)-1,2,3,4-tetrahydroacridin-9-amine) has the potential to serve as an effective ChE inhibitor in the context of the tacrine-resveratrol fused hybrids study, which is focused on the development of novel therapeutic strategies for the treatment of AD. In the study on tacrine-resveratrol fused hybrids, Compound **5** exhibited an IC_50_ value of 0.80 μM at a 10 mM concentration for human acetylcholinesterase (hAChE) and demonstrated a weak interaction with the Peripheral Anionic Site (PAS) of the enzyme. Moreover, it has been shown to impede Aβ self-aggregation, a pivotal process in the pathogenesis of AD, and has exhibited notable antioxidant activity. In the study of the tacrine-resveratrol fused hybrid, compound **8** (5-(4-(6-chloro-1,2,3,4-tetrahydroacridin-9-ylamino)phenethyl)benzene-1,3-diol) was identified as a potential inhibitor of hAChE, exhibiting an IC_50_ value of 1.3 μM. The efficacy of Compound **8** in inhibiting Aβ self-aggregation was demonstrated with an inhibition rate of 37.3%. In the study of the tacrine-resveratrol hybrid, compound **12** (5-(4-((6-chloro-1,2,3,4-tetrahydroacridin-9-yl)amino)styryl)benzene-1,3-diol) demonstrated inhibitory activity against hAChE with an IC_50_ of 8.8 μM. Furthermore, compound **12** demonstrated efficacy in inhibiting Aβ self-aggregation, with an inhibition rate of 31.2%. The findings of the study suggest that compound **12** has the potential to serve as both a ChE inhibitor and an anti-aggregation agent, which could indicate a role for it in the treatment of AD. The data indicate that compounds **8** and **12** exhibit enhanced inhibitory activity against Aβ self-aggregation and ChE compared to other derivatives under investigation [271].

This study investigates the potential of synthetic stilbene hybrids in the treatment of AD, demonstrating that the hybrid design approach can enhance biological activity. Of particular note is hybrid 12, which exhibits a low neurotoxicity profile; certain derivatives demonstrate effects such as cholinesterase inhibition and the prevention of amyloid aggregation. Nevertheless, some hybrids have limitations such as toxicity risk (especially hepatotoxicity) and low antioxidant activity compared to natural stilbenes. In conclusion, this study provides new candidates for the treatment of AD, but emphasizes that further optimization is required in terms of safety and efficacy.

In a recent study, Arbo et al. (2020) explored the potential therapeutic applications of resveratrol and its derivatives in the treatment of AD and PD [272]. The study examined the neuroprotective effects of resveratrol in experimental models of these neurodegenerative conditions, emphasizing its antioxidant and anti-inflammatory properties. Modifying the structure of the resveratrol molecule, for example, through glycosylation, alkylation, halogenation, hydroxylation, methylation, and prenylation, may result in derivatives with enhanced bioavailability and pharmacological activity. Derivatives of resveratrol have demonstrated potential for the inhibition of Aβ aggregation, antioxidant and metal chelating properties, and protection against oxidative stress. Furthermore, they have demonstrated neuroprotective effects. The identification of Compounds **1** and **2** as potential lead compounds for the treatment of AD was based on their significant inhibition of Aβ aggregation, antioxidant activity, and moderate AChE inhibition. These compounds, specifically 2-((4-(3,5-dimethoxystyryl)phenylamino)methyl)-4-(dimethylamino)phenol (Compound **1**) and (E)-5-(4-(5-(dimethylamino)-2-hydroxybenzyl amino)styryl)-benzene-1,3-diol (Compound **2**), were selected for further investigation due to their promising properties. In vitro studies demonstrated that Compounds **1** and **2** are capable of crossing the blood–brain barrier and exhibited low toxicity in mice, indicating that they are promising candidates for the treatment of AD. Compound **3** (4-(((2-hydroxyphenyl)imino)methyl)benzene-1,2-diol) was identified as a promising agent for the treatment of AD, demonstrating efficacy as a neuroprotectant and superior performance to resveratrol in neuroprotection. Compound **3** was observed to inhibit Aβ aggregation, act as an antioxidant and metal chelator, and demonstrate neuroprotective effects against hydrogen peroxide in neuroblastoma cells [272].

The present study aims to contribute to the development of drugs for AD by analyzing the neuroprotective mechanisms of synthetic stilbene derivatives. The analysis will demonstrate how structural modifications, especially prenylation, can enhance the compounds’ bioavailability and pharmacological efficacy, while providing targeted protective effects against Aβ and tau aggregations. The structure–activity relationships of these hybrid derivatives provide important information for pharmaceutical development. While synthetic derivatives offer significant advantages in terms of specific target-specific effects and reduced side effects, potential side effects such as immunosuppression and toxicity, and long-term safety deficiencies are fundamental limitations. Furthermore, the direct transferability of in vitro and animal model data to human applications is limited. Consequently, there is a necessity for comprehensive mechanism studies and advanced clinical studies to facilitate a more profound comprehension of the therapeutic potential of synthetic stilbenes.

The studies have provided focus on hybrid compounds incorporating resveratrol and their potential therapeutic effects on AD. The two studies indicate the potential therapeutic efficacy of hybrid compounds in the treatment of AD, although they differ in their methodological approaches. The research conducted by Jeřábek et al. (2017) concentrated on the dual inhibition of AChE and Aβ aggregation [271]. In contrast, Arbo et al. emphasized the neuroprotective properties of resveratrol derivatives, particularly their antioxidant, anti-inflammatory, and metal-chelating capabilities [272]. Both sets of compounds demonstrate potential, but the compounds developed by Arbo appear to offer more comprehensive protection beyond AChE inhibition, addressing multiple pathways involved in AD pathogenesis. In a study published in 2019, Tang et al. investigated the synthesis and evaluation of isoprenylation-resveratrol dimer derivatives for their potential efficacy in the treatment of AD [273]. The research concentrated on the biological activities of these derivatives, including their inhibitory activity against human monoamine oxidase B (hMAO-B) and antioxidant effects. The resveratrol dimer derivatives synthesized by Tang et al. were found to exhibit moderate inhibitory activity against hMAO-B [T4]. It is noteworthy that compounds **3** and **7** exhibited superior inhibitory activity for hMAO-B compared to other compounds tested. Compound **3** is 5-((E)-2-(3-(3,5-dihydroxy-4-(3-methylbut-2-en-1-yl)phenyl)-2-(4-hydroxyphenyl)-2,3-dihydrobenzofuran-5-yl)vinyl)-2-(3-methylbut-2-en-1-yl) benzene-1,3-diol, while compound **7** is 5-((E)-2-(3-(5-hydroxy-2, The 2-dimethylchroman-7-yl)-2-(4-hydroxyphenyl)-2,3-dihydrobenzofuran-5-yl)vinyl)-2-(3-methylbut-2-en-1-yl)benzene-1,3-diol exhibited superior inhibitory activity for hMAO-B compared to the other compounds tested. The study involved the modification of the resveratrol structure through the introduction of an isoprene group, resulting in the formation of dimer derivatives. Resveratrol was employed as the substrate for this modification, and the isoprene group was successfully introduced to yield the target compound **4**. Subsequently, compound **4** was dimerized to yield compound **3**. This modification strategy was developed based on the formation of the dehydrodimer of resveratrol. The inhibitory activity of compounds **3** and **7** against hMAO-B is notable, and this could prove advantageous in the treatment of AD. The inhibition of hMAO-B by these compounds may assist in the reduction of oxidative stress, which is a pivotal factor in the progression of AD. Inhibition of hMAO-B can exert neuroprotective effects and prevent the degradation of vital neurotransmitters, such as dopamine and serotonin, which are essential for optimal brain function. The selectivity of compounds **3** and **7** for hMAO-B over hMAO-A is of particular importance in the management of AD. The antioxidant effects of compounds **3** and **7** were then compared. Both compounds demonstrated a reduction in toxicity and enhanced resilience to neurotoxicity induced by oxidative toxins, including H_2_O_2_, rotenone, and oligomycin-A. It is noteworthy that compound **3** demonstrated a slightly enhanced antioxidant capacity compared to compound **7** in a range of assays, including DPPH, ABTS, and FRAP assays. Moreover, it is noteworthy that compound **3** demonstrated substantial biological activity and was highly efficacious in protecting neuronal cells against oxidative toxins and inflammation induced by LPS stimulation and H_2_O_2_ stimulation. This performance was superior to that of compound **7**. Compound **3** exhibited superior permeability across the blood–brain barrier, indicating its potential as a neuroprotective agent for the treatment of AD [273].

Tang et al. (2019) conducted an evaluation of the cytotoxic concentrations of compounds **3** and **7**, with the objective of determining their impact on cell viability [273]. The compounds were evaluated at a concentration of 30 mM on PC12 cells to assess their cytotoxicity. The results demonstrate that the majority of the compounds tested did not exhibit significant neurotoxicity at this concentration. This indicates that compounds **3** and **7** exhibit reduced toxicity and are well-tolerated by the cells. This information is of great importance for the comprehension of the safety profile of these compounds and their potential for further development as neuroprotective agents. In conclusion, although both compounds exhibited antioxidant effects and neuroprotective properties, it can be argued that compound **3** demonstrated a slightly superior antioxidant capacity and biological activity compared to compound **7** in the cellular assay. Moreover, both compounds exhibited remarkable antioxidant effects in cellular assays. The results of this study indicate that these derivatives may have the potential to serve as neuroprotective agents in the treatment of AD [273]. This study evaluates the neuroprotective effects of synthetic stilbene derivatives (with particular reference to compounds **3** and **7**) against AD. These derivatives exhibit lower toxicity and higher neuroprotective activity compared to natural stilbenes, and are also distinguished by their ability to reduce ROS and cross the blood–brain barrier. The presence of an isoprenyl group, a characteristic feature of certain structural modifications, has been identified as a significant factor contributing to the observed biological activity. However, the study acknowledges certain limitations, including target-specific effects and the potential for adverse effects. Furthermore, the absence of in vivo data engenders uncertainty with regard to clinical applications. Consequently, further in vivo studies and mechanistic analyses are required to fully elucidate their potential therapeutic applications.

Li et al. (2014) investigated the design, synthesis, and biological evaluation of iminoresveratrol derivatives as multi-targeting agents against AD [274]. The study outlines the design and synthesis of imine resveratrol derivatives (compounds **1**–**20**) using a classical method of imine formation. The method entails the condensation of aromatic amines and aromatic aldehydes in ethanol under reflux conditions, yielding imines with an (E)-configuration as the sole product. In their 2014 study, Li et al. demonstrated that the imine resveratrol derivatives they had synthesized were effective in inhibiting Aβ aggregation, which is a crucial factor in the progression of AD. The compounds demonstrated considerable efficacy in inhibiting both spontaneous and Cu^2^⁺-induced Aβ aggregation. Compound **9** ((E)-4-(((2-hydroxyphenyl)imino)methyl)benzene-1,2-diol) has been identified as a highly promising treatment for AD, given that it has demonstrated potent inhibition of Aβ_1-42_ aggregation (68.1%). Furthermore, the compound displays antioxidant properties, which serve to reinforce its potential therapeutic value. The antioxidant activity of compound **9** was found to be noteworthy, with an IC_50_ of 14.1 mM, as determined by the DPPH free radical method. This activity is crucial in reducing oxidative stress, which is a pivotal factor in the pathogenesis of AD. It has been demonstrated that compound **9** is capable of effectively chelating metal ions, including Cu^2+^ and Fe^2+^, which in turn inhibits Cu^2+^-induced Aβ_1-42_ aggregation. Furthermore, it has been observed that the compound reduces the production of hydrogen peroxide (H_2_O_2_) by Cu^2^⁺-Aβ_42_ species, which suggests its potential to regulate the production of ROS. The multifunctional properties exhibited by compound **9** render it a highly promising candidate for the treatment of AD. The synthesized imine resveratrol derivatives have demonstrated the potential to inhibit Aβ aggregation and act as antioxidants, indicating their potential as treatment options for AD [274]. Li et al. (2014) focus on the neuroprotective capacity of synthetic stilbene derivatives and the advantages and disadvantages of these compounds as potential AD drugs compared to natural stilbenes. Synthetic stilbenes stand out as promising candidates for AD treatment, mainly due to their ease of design and modification, as they offer multiple targeting mechanisms such as inhibition of Aβ aggregation, chelation of metal ions, and reduction in oxidative stress. Structural variations in these hybrid derivatives can have significant effects on their activity profiles. For example, the presence of imine groups and metal binding sites can increase the affinity of stilbene, which may enhance the neuroprotective effect. However, the chemical stability and bioavailability of these compounds compared to natural stilbenes are among the major challenges. In addition, the potential toxicity and side-effect profile of some synthetic derivatives may be a major obstacle to clinical application. Strengths of the study include the development of novel molecules that can target different neuroprotective mechanisms and the efficacy of these compounds demonstrated by in vitro studies. However, weaknesses include the fact that the in vivo efficacy and safety of these molecules have not been fully evaluated, and their clinical validity is uncertain. In conclusion, this review demonstrates the potential of synthetic stilbenes in the treatment of AD, but also highlights the need for further clinical and preclinical research [274].

The studies by Tang et al. (2019) and Li et al. (2014) both focus on the synthesis and assessment of resveratrol derivatives for the treatment of AD [273,274]. The two studies emphasize the potential of resveratrol derivatives for the treatment of AD, although they adopt disparate approaches. Tang et al. (2019) focused on the inhibition of hMAO-B and the antioxidant properties of isoprenylation-resveratrol dimers. It was observed that compound **3** demonstrated enhanced biological activity and blood–brain barrier permeability [273]. The study by Li et al. (2014) focused on investigating the inhibitory effect of imine-resveratrol derivatives on Aβ aggregation, their capacity for metal chelation, and their antioxidant potential [274]. The researchers identified compound **9** as a promising candidate for the treatment of AD due to its multifunctional properties. In conclusion, Tang et al. investigated the neuroprotective effects of MAO-B inhibition and antioxidant activity, whereas Li et al. emphasized the pivotal role of Aβ aggregation inhibition and metal chelation as therapeutic mechanisms. While both studies have identified promising compounds, their focus is on disparate pathological targets in AD [273,274]. Lu et al. (2013) design, synthesize, and assess multi-target resveratrol derivatives to treat AD [275]. The synthesized resveratrol derivatives were found to significantly inhibit self-induced Aβ aggregation and Cu(II)-induced Aβ_1−42_ aggregation. Furthermore, these compounds exhibited antioxidant activity and the capability to chelate biometals such as Cu(II), Fe(II), Fe(III), and Zn(II). Compounds **5d** ((E)-2-((4-(3,5-dimethoxystyryl)phenylamino)methyl)-4-(dimethylamino)phenol) and **10d** ((E)-5-(4-(2-Hydroxy-5-nitrobenzylamino)styryl)benzene-1,3-diol) have been identified as potential lead compounds for AD therapy. The derivatives showed significant antioxidant activity, with Trolox equivalents ranging from 2.37 to 6.27, indicating their effectiveness in scavenging free radicals. Additionally, compounds **5d** and **10d** demonstrated noteworthy inhibition of Aβ aggregation, with IC_50_ values of 7.56 μM and 6.51 μM, respectively, for self-induced Aβ aggregation. Furthermore, the derivatives, including compounds **5d** and **10d**, have exhibited metal-chelating properties by binding to biometals such as Cu(II), Fe(II), Fe(III), and Zn(II). Additionally, compound **5d** has been observed to cross the blood–brain barrier in vitro, indicating its potential for drug delivery to the central nervous system. The study shows that the multitarget-directed resveratrol derivatives possess antioxidant activity, inhibit Aβ aggregation, chelate metal, and permeate the blood–brain barrier. In particular, compounds **5d** and **10d** have exhibited pronounced inhibitory effects on Aβ aggregation, with IC_50_ values of 7.56 μM and 6.51 μM, respectively. They have also effectively impeded both self-induced and Cu(II)-induced Aβ aggregation, with 87.51% and 94.23% inhibition, respectively. The results suggest that compounds **5d** and **10d** may be capable of targeting multiple pathways involved in Aβ aggregation and neurotoxicity. These findings illustrate the considerable potential of these compounds in the field of Aβ aggregation research. They have demonstrated the capacity to inhibit Aβ aggregation, act as antioxidants, and disassemble Aβ fibrils generated by self- and Cu(II)-induced Aβ aggregation. The in vitro crossing of the blood–brain barrier and the absence of acute toxicity in mice at high doses indicate promising results for compound **5d** [275]. The finding is of significant importance, as it has the potential to serve as a lead compound for the treatment of AD. It is important to note that a drug’s ability to traverse the blood–brain barrier (BBB) is a crucial factor in its efficacy in treating central nervous system disorders such as AD, as it allows the drug to reach the brain and target the underlying mechanisms of the disease. This finding may contribute to the development of more efficacious treatments for AD. The capacity of compound **5d** to traverse the BBB indicates the potential for direct access to the brain, which could enhance its efficacy in addressing the pathological processes associated with AD and improve treatment outcomes [275].

Chao et al. (2010) compared the neuroprotective effects of pinostilbene, a methylated derivative of resveratrol, with those of resveratrol and other derivatives against 6-hydroxydopamine-induced neurotoxicity in SH-SY5Y cells, suggesting that pinostilbene might have potential as a neuroprotective agent [276]. The study demonstrated that exposing neurons to 25 μM 6-hydroxydopamine (6-OHDA) resulted in a 4.1 ± 0.0-fold increase in LDH activity and a 2.4 ± 0.1-fold increase in caspase-3 activity in comparison to the control group. These findings indicate that resveratrol may have a potential therapeutic effect in reducing neurodegeneration caused by 6-OHDA. Furthermore, the study demonstrated that pretreatment with resveratrol at concentrations of 1 to 10 μM markedly diminished LDH release and caspase-3 activity, with 1 μM being the most efficacious dosage. The results are presented with a balance of confidence and diplomacy, acknowledging the potential implications of the study without overstating them. In comparison to resveratrol, the methylated derivative R3 exhibited superior efficacy in the reduction of 6-OHDA-induced LDH release and the elevation of caspase-3 activity across a broad concentration range of 0.1–10 μM. Furthermore, it is noteworthy that exposure to 6-OHDA resulted in a significant reduction in the phosphorylation of mTOR and GSK-3β in neurons. However, this effect was successfully reversed by pretreatment with either resveratrol or R3. The findings of the study indicate that pinostilbene may be a highly efficacious neuroprotective agent, exhibiting a broader range of effective concentrations and more pronounced properties than resveratrol. The results demonstrate that pinostilbene significantly reduced LDH release and caspase-3 activity in a dose-dependent manner, indicating its potential for neuroprotective purposes. Furthermore, pinostilbene has been demonstrated to enhance bioavailability and reduce the phosphorylation of JNK and c-Jun induced by 6-OHDA, indicating its potential as a neuroprotective agent [276]. This study investigates the neuroprotective potential of synthetic stilbenes in relation to AD, and draws parallels with their natural counterparts. In particular, the investigation reveals that methylated derivatives exhibit the capacity to enhance bioavailability and target cellular pathways such as JNK and mTOR, thereby promoting cell survival. Noteworthy among the derivatives are pinostilbene and its derivatives, which demonstrate a more extensive range of activity. However, the full scope of their effects and the mechanisms underlying their activity remain to be fully elucidated, and their direct transferability from in vitro to clinical applications remains limited. Consequently, further research is necessary to thoroughly examine the mechanisms involved.

Belmonte-Reche et al. (2021) studied the neuroprotective activity of O-silyl resveratrol derivatives against oxidative stress using SH-SY5Y neuroblastoma cells [277]. Additionally, the toxicity and neuroprotective capacity of these derivatives were investigated in zebrafish models challenged with hydrogen peroxide and pentylenetetrazole (PTZ), respectively. The therapeutic effects of the compounds were also evaluated in a mouse model of neurodegenerative disease, specifically Huntington’s disease. The study revealed that O-silyl resveratrol derivatives exhibited enhanced neuroprotective efficacy compared to resveratrol alone. Prodrugs of these derivatives were designed and prepared with the objective of enhancing their bioavailability for potential therapeutic use. The research evaluated the toxicity, neuroprotective capacity, and anti-inflammatory activity of the silyl resveratrol derivatives in vitro and in zebrafish embryo models. The di-triethylsilyl and di-triisopropylsilyl RES derivatives demonstrated superior in vitro neuroprotective and anti-inflammatory properties compared to resveratrol. Among the O-silyl RES derivatives and their corresponding prodrugs, compound **26** (3,5-triethylsilyl-4′-(6-O-octanoylglucopyranosyl) resveratrol) exhibited the most favorable profile in terms of toxicity and neuroprotective activity in zebrafish embryo assays. The administration of compound **26** was observed to mitigate the loss of motor coordination in a 3-nitropropionic acid mice model of Huntington’s disease, exhibiting a profile of efficacy comparable to that of resveratrol. Compound **26** demonstrated a greater reduction in the pro-inflammatory cytokine IL-6 in comparison to resveratrol, and exhibited an improved performance in the rotarod test by 10%. Furthermore, compound **26**, a silyl resveratrol prodrug with the chemical formula C_20_H_26_O_3_Si, has been identified as the most effective resveratrol derivative. Its performance in preclinical models of Huntington’s disease and multiple sclerosis has yielded promising results, indicating its potential as a therapeutic option for these neurodegenerative conditions. In an experimental autoimmune encephalomyelitis (EAE) multiple sclerosis mouse model, compound **26** was observed to significantly reduce the progression of EAE severity and the percentage of animals with moderate to severe clinical scores. Moreover, all measured compounds demonstrated low toxicity in zebrafish embryo toxicity studies, with LC_50_ values falling between those of resveratrol and piceid octanoate 2 [277]. This report provides a comprehensive review of the neuroprotective effects of silyl derivatives and their advantages and disadvantages as potential AD drugs. The synthetic stilbene derivatives examined have been developed through various chemical modifications compared to natural stilbenes and claim to have increased bioavailability and efficacy. The addition of silyl groups has been demonstrated to enhance the neuroprotective activity of these compounds and improve their anti-inflammatory potential. This study systematically explores the relationship between the structural features and functional properties of these hybrid derivatives. It has been determined that structural features of the derivatives, such as the presence of silyl groups, have important effects on neuroprotection. For instance, certain silyl compounds have been shown to possess advantageous properties, such as the capacity to reduce oxidative stress and enhance cell viability in both in vitro and in vivo models. Conversely, certain derivatives have been observed to exhibit disadvantages, including high lipophilicity or the potential for toxic effects. The study’s strengths lie in its comprehensive evaluations in both cellular and animal models and the detailed data provided on the mechanisms of neuroprotection. However, the study is not without its limitations, which include the side effects of certain silyl compounds and uncertainties about their efficacy that may not directly translate to clinical practice. In conclusion, this study, along with analogous studies, suggests that synthetic stilbene derivatives should be investigated further as potential AD treatment options.

The studies by Lu et al. (2013), Chao et al. (2010), and Belmonte-Reche et al. (2021) all investigate the potential of different resveratrol derivatives as a treatment for neurodegenerative disorders such as AD and Huntington’s disease (HD) [275,276,277]. The objective of each study is to synthesize, evaluate and assess the potential therapeutic applications of these compounds, with a particular focus on their antioxidant and neuroprotective properties. Lu et al. (2013) concentrated their research on the inhibition of Aβ aggregation and metal chelation as potential treatments for AD, with compound **5d** exhibiting encouraging results in crossing the BBB [275]. Chao et al. (2010) demonstrated that pinostilbene exhibited superior neuroprotective effects compared to resveratrol in reducing oxidative stress and neurotoxicity, particularly in Parkinson’s-like models [276]. Belmonte-Reche et al. (2021) emphasized the potential of O-silyl resveratrol derivatives, particularly compound **26**, as neuroprotective and anti-inflammatory agents, with applications in the treatment of Huntington’s disease and multiple sclerosis [277]. Each study provides valuable insights into different therapeutic targets and strategies for addressing neurodegeneration. Yuan et al. (2014) focused on the synthesis and evaluation of pterostilbene and resveratrol carbamate derivatives as potential dual ChE inhibitors and neuroprotective agents [278]. The researchers designed a series of novel carbamate derivatives of pterostilbene and resveratrol with the objective of creating compounds that would exhibit ChE inhibitory activity, antioxidant properties, and neuroprotective effects. The synthesized compounds were evaluated for their inhibitory potency against AChE and butyrylcholinesterase (BChE), as well as their neuroprotective effects against hydrogen peroxide-induced PC12 cell injury in vitro. The study revealed that certain compounds exhibited dual inhibitory potency against AChE and BChE, with compound **7h** (N-benzylpiperazine-N’-carbonyl) displaying particularly promising activity in both aspects. Furthermore, the neuroprotective effects of the synthesized compounds were investigated, with compound **7h** exhibiting potential in this regard. In their study on pterostilbene and resveratrol carbamate derivatives, Yuan et al. (2014) presented a series of key findings with numerical values. The reference compound rivastigmine tartrate exhibited IC_50_ values of 6.3 µM for AChE and 1.37 µM for BChE. The compounds **2** (pterostilbene) and 3 (resveratrol) did not exhibit any activity against AChE and BChE. Compound **7h** exhibited the most potent inhibitory activity, with IC_50_ values of 4.11 µM for AChE and 1.13 µM for BChE, which surpassed the reference compound’s values [278]. Furthermore, compounds **6g** and **7g** exhibited moderate AChE inhibitory activity, with IC_50_ values of 8.58 µM and 6.97 µM, respectively. With regard to neuroprotective effects, compound **7h** demonstrated a cell viability of 81.30% in the context of hydrogen peroxide-induced PC12 cell injury, indicating the potential for neuroprotective properties. The varying cell viability percentages of other compounds also reflected their diverse degrees of neuroprotective effects. These results provide quantitative insights into the ChE inhibitory activity and neuroprotective potential of the synthesized compounds, suggesting their promise as multi-target-directed agents for the treatment of AD [278].

Jung et al. (2009) conducted a study on the synthesis of novel trans-stilbene derivatives and evaluated their potent antioxidant and neuroprotective effects [279]. Synthetic strategies employed included the Wittig–Horner reaction for olefin generation and a coupling reaction for trans-stilbene derivative production, with high yields achieved. One of the derivatives, specifically the amide derivative **15g** (trans-3,4-dihydroxy-40-[N-(2-fluorobenzyl)aminocarbonyl]stilbene), exhibited free radical-scavenging activity in vitro that was three times greater than that of resveratrol. Furthermore, another derivative, **15d** (trans-3,4-dihydroxy-40-[N-(furan-2-ylmethyl)aminocarbonyl]stilbene), demonstrated potent inhibitory activity against lipopolysaccharide (LPS)-induced nitric oxide (NO) generation. The allylamide analog **15a** (trans-3,4-dihydroxy-40-(N-allylaminocarbonyl)stilbene) demonstrated the most potent neuroprotective activity in glutamate-induced primary cortical neuron cells. In a study conducted by Jung et al. (2009), it was observed that the majority of trans-stilbene derivatives exhibited radical-scavenging activity that was 2–3 times greater than that of resveratrol [279]. Specifically, the amide derivative **15g** exhibited a free radical-scavenging activity that was three times greater than that of resveratrol in vitro. Furthermore, the scavenging ratios were found to be favorable, ranging from 30 to 75% at the highest concentration (200 mM), for compounds **15a–n**, 17 (trans-3,4-dihydroxy-40-(methoxycarbonyl)stilbene) and 18 (trans-3, The study also investigated the potential of 4-hydroxy-40-[2-(benzamido)acetoxy methyl]stilbene (**22**), trans-3,4-dihydroxy-40-(methoxycarbonyl-1-ethenyl)stilbene (**24**), trans-3,4-dihydroxy-40-(3-hydroxyprop-1-enyl)stilbene (**26**), and trans-3,4-dihydroxy-40-(2-formyl-1-ethenyl)stilbene (**28**). These compounds exhibited potent neuroprotective effects through the scavenging of free radicals and the inhibition of nitrite production, which are pivotal in the protection of neurons from excitotoxicity and inflammatory damage. The capacity of these derivatives to impede nitric oxide (NO) generation in microglia cells and avert excitotoxic neuronal demise indicates their prospective utility in attenuating neurodegenerative processes and conferring neuroprotection [279]. In this investigation, the analyses pertaining to the neuroprotective effects of synthetic stilbene derivatives are undertaken to illuminate both the advantages and disadvantages of the use of these compounds for the treatment of potential AD as opposed to natural stilbenes. Synthetic derivatives exhibit augmented biological activity through structural modifications. These modifications, including optimization of hydrogen bonds and electron density at specific sites, have been shown to enhance neuroprotective mechanisms. However, the advantages of these derivatives over natural stilbenes include better bioavailability and targeted action mechanisms. For instance, given the limited bioavailability and metabolism processes of natural stilbenes, synthetic derivatives have the potential to ameliorate these issues. Nevertheless, concerns persist regarding the toxicity and long-term effects of synthetic compounds. The potential for adverse effects at the cellular level may potentially negate the intended neuroprotective benefits. Furthermore, a more detailed investigation at the molecular level is required to establish the relationship between the structures and activities of hybrid derivatives. In particular, understanding the activity changes obtained by adding different functional groups is of great importance in the design of new adjuvants. Notwithstanding, the absence of a comprehensive understanding of the underlying mechanisms represents a significant lacuna within this domain. While there has been progress in the optimization of various neuroprotective pathways, such as the free radical scavenging effect, the full insight and clinical effects of these mechanisms remain to be elucidated. Consequently, while synthetic stilbene derivatives offer numerous advantages over natural derivatives in terms of neuroprotection, potential risks and as yet unelucidated mechanisms may limit the clinical application of this generation of health products. Consequently, further research is imperative to comprehensively evaluate the safety and efficacy of these compounds.

The studies by Yuan et al. (2014) and Jung et al. (2009) both focus on the synthesis and assessment of resveratrol or trans-stilbene derivatives with the objective of determining their potential as neuroprotective agents and treatments for AD [278,279]. However, the two studies target disparate mechanisms of neuroprotection and neurodegenerative processes. Yuan et al. (2014) concentrated their efforts on the synthesis of pterostilbene and resveratrol carbamate derivatives. These were designed with the specific intention of acting as dual ChE inhibitors (inhibiting both AChE and BChE), while also offering neuroprotective effects against oxidative stress [278]. The objective of synthesizing carbamate derivatives was to enhance ChE inhibition and antioxidant capacity. In a different approach, Jung et al. (2009) synthesized trans-stilbene derivatives through the use of strategies such as the Wittig-Horner reaction and coupling reactions, resulting in the production of significant quantities of olefins and trans-stilbenes [279]. The objective was to enhance antioxidant activity and neuroprotection by scavenging free radicals and inhibiting inflammatory markers such as nitric oxide (NO). The two studies investigated resveratrol-related derivatives for their neuroprotective effects, although they targeted different aspects of neurodegeneration. Yuan et al. (2014) focused on ChE inhibition and neuroprotection against oxidative stress, with compound **7h** exhibiting potential for the treatment of AD [278]. In contrast, Jung et al. (2009) concentrated on antioxidant activity and NO inhibition in inflammatory and excitotoxic models, with compounds **15a** and **15g** demonstrating robust neuroprotective and antioxidant effects [279]. The studies are mutually reinforcing, as one focuses on ChE inhibition while the other emphasizes antioxidant and anti-inflammatory mechanisms, both of which are pivotal in the treatment of neurodegenerative disorders. Mlakić et al. (2022) investigated the synthesis and characterization of novel hybrids of resveratrol-thiophene and resveratrol-maltol as ChE inhibitors and antioxidants [280]. The inhibitory and antioxidant properties of these compounds were evaluated through a series of experiments and compared to a number of reference drugs, including galantamine. Moreover, the researchers investigated the compounds’ capacity to form complexes with biometals and analyzed their crystal structures to ascertain their potential for medical applications. The resveratrol-thiophene hybrids, specifically heteroaromatic resveratrol analogs with one or more hydroxyl groups (compounds II and III), exhibited notable ChE inhibitory activity. The IC50 value for AChE inhibition for compound II was 15.7 μM, while the IC50 value for BChE inhibition was 4.6 μM for compound III. Additionally, compound III demonstrated antioxidant activity, with an IC_50_ value of 26.8 μM. In contrast, the resveratrol-maltol hybrids demonstrated comparatively weaker inhibitory potency. Only four of the 12 tested hybrids demonstrated activity towards BChE at relatively high IC_50_ concentrations. Of the resveratrol-maltol hybrids, only the trans-1 derivative demonstrated antioxidant activity, with a measurable IC50 value. The remaining derivatives exhibited IC50 values exceeding 1000 μM, indicating a lack of antioxidant efficacy. In conclusion, the crystal structure analyses of the synthesized compounds provide a more nuanced understanding of their interactions with ChEs. This paves the way for the rational design of more potent and selective inhibitors for therapeutic applications in neurodegenerative disorders such as AD. (Compounds II and III are derivatives of resveratrol with thiophene substitutions. They differ primarily in their functional groups and structural configurations. Compound II has an OH group at the ortho position and a trans-geometry of the stilbene moiety, while compound III has an additional OH group at position 4 on the phenyl moiety) [280].

Lan et al. (2018) focused on developing new compounds for treating AD. The synthesis of resveratrol-indazole hybrids was undertaken with the objective of inhibiting monoamine oxidases (MAOs) and Aβ aggregation, both of which are implicated in the disease’s pathogenesis [281]. The compound **6e** was identified as a potent inhibitor of both MAO-B and Aβ_1-42_ self-aggregation. The compounds **6e** (N-(3,4-dichlorobenzylidene)-1H-indazol-5-amine) and 9(3-(1H-indazol-5-yl)imino)methyl)-4H-chromen-4-one) were observed to exhibit no toxicity towards nerve cells and were able to penetrate the blood–brain barrier, indicating their potential as drug candidates for neurodegenerative disorders. The most potent inhibitor of both hMAO-B and Aβ_1-42_ self-aggregation was identified as compound **6e**. It exhibited competitive inhibition of hMAO-B with an IC_50_ value of 1.14 μM, thereby demonstrating its strong inhibitory activity against this enzyme. At a concentration of 20 μM, compound **6e** demonstrated a significant reduction of 58.9% in Aβ_1-42_ self-aggregation inhibition, indicating a potent inhibitory effect on Aβ aggregation. Moreover, no toxicity was observed in PC12 cells at concentrations ranging from 6.25 μM to 100 μM, indicating the compound’s potential for safe therapeutic use. The findings of the study indicate that compounds **6e** and **9** can protect neurons by inhibiting MAO-B, reducing Aβ aggregation, and providing anti-inflammatory and antioxidant effects. These compounds are capable of crossing the blood–brain barrier, which allows for direct effects on brain tissue. This makes them potential neuroprotective agents for neurodegenerative disorders [281]. This study investigates the neuroprotective properties of synthetic stilbene derivatives in relation to AD, and compares them with natural stilbenes with regard to their advantages and limitations. The design, synthesis and biological evaluation of resveratrol-indazole hybrids was conducted; these compounds were shown to inhibit Aβ aggregation and MAO enzyme. Molecular modeling and biological tests revealed that hydrogen bonds and π-π interactions play an important role in their mechanism of action. The study also found that the low toxicity levels of the compounds are positive in terms of potential safety. However, the study was limited to in vitro level, creating uncertainties for clinical application. Selectivity issues observed in certain compounds and the necessity for further exploration of structure–activity relationships represent significant limitations. Nevertheless, the findings obtained with the multi-target drug development strategy provide a promising basis for AD treatment.

The studies by Mlakić et al. (2022) and Lan et al. (2018) both investigate hybrid resveratrol derivatives designed to target neurodegenerative disorders, particularly AD [280,281]. However, the studies differ in their focus on different mechanisms of action, including ChE inhibition, MAO inhibition, Aβ aggregation, and antioxidant effects. Mlakić et al. (2022) synthesized resveratrol-thiophene and resveratrol-maltol hybrids and evaluated their ChE inhibition and antioxidant properties [280]. The hybrids were designed to target AChE and BChE, enzymes that play a role in the pathology of AD. In a further approach, Lan et al. developed resveratrol-indazole hybrids with the objective of targeting both MAO-B and Aβ aggregation. The objective of these compounds was to inhibit key processes that are implicated in the development of AD, including the degradation of neurotransmitters and the formation of amyloid plaques. Mlakić et al. (2022) determined that Compound II exhibited AChE inhibition with an IC_50_ of 15.7 μM, while Compound III demonstrated potent BChE inhibition with an IC_50_ of 4.6 μM. These findings highlight the efficacy of resveratrol-thiophene hybrids in targeting ChEs, which play a pivotal role in AD by facilitating the breakdown of neurotransmitters such as acetylcholine [280]. The study by Lan et al. (2018) did not focus on the inhibition of ChEs, but rather on the inhibition of MAO-B. Compound **6e** demonstrated an IC_50_ of 1.14 μM for hMAO-B, exhibiting potent inhibition compared to numerous standard drugs employed for the management of AD symptoms through the regulation of monoamine neurotransmitters. The two studies contribute to the advancement of novel resveratrol-based hybrids for neurodegenerative disease therapy, although they focus on disparate aspects of AD pathology [281]. Mlakić et al. (2022) focused their attention on the inhibition of ChEs and the antioxidant activity of resveratrol-thiophene hybrids. The findings revealed that Compound III was the most efficacious in inhibiting BChE, while Compound II demonstrated moderate inhibition of AChE. The objective of the study, as outlined by Lan et al. (2018), was to target MAO-B inhibition and Aβ aggregation [280,281]. Compound **6e** demonstrated a distinctive dual inhibitory action against MAO-B and Aβ_1-42_ aggregation, exhibiting robust neuroprotective potential and safety in cell models. In general, Mlakić et al. concentrated on the inhibition of enzymes and antioxidant properties pertinent to the regulation of neurotransmitters in AD, whereas Lan et al. focused on multiple pathways, including MAO-B and Aβ aggregation, with the objective of addressing both oxidative stress and amyloid plaque formation in AD. Mellado et al. (2022) conducted a study on the development of coumarin-resveratrol-inspired hybrids as potential inhibitors of MAO-B [282]. The objective of the study was to undertake a comparative analysis of the inhibitory activities of two compounds, 3-phenylcoumarin and trans-6-styrylcoumarin, in conjunction with trans-resveratrol. The research involved the design, synthesis and evaluation of these compounds for their MAO inhibitory activity. Computational studies, including CoMFA, CoMSIA, and molecular docking, were employed to examine the interactions between these molecules and the active sites of MAO isoforms. Trans-6-styrylcoumarin demonstrated notable potency as an inhibitor of MAO-B. The compound demonstrated a potency that was 107 times greater than that of 3-phenylcoumarin and 267 times greater than that of trans-resveratrol. Moreover, trans-6-styrylcoumarin displayed a high degree of MAO-B selectivity, with a pIC50 value of 6.959. The QSAR models developed in the study demonstrated high predictive power, with q2 values ranging from 0.523 to 0.841 and r2 test values ranging from 0.805 to 0.911. In conclusion, the study by Mellado et al. (2022) provides valuable insights into the development of novel MAO-B inhibitors, with a particular focus on the comparison between 3-phenylcoumarin and trans-6-styrylcoumarin [282]. The research underscores the notable potency of trans-6-styrylcoumarin as a selective MAO-B inhibitor, exhibiting substantially greater activity than 3-phenylcoumarin and trans-resveratrol. The study employed sophisticated computational techniques, including 3D-QSAR models, molecular docking, and molecular dynamics simulations, to examine the interaction of these compounds with MAO-B. The results indicate that trans-6-styrylcoumarin has the potential to be developed further as a therapeutic agent for neurodegenerative disorders, particularly Parkinson’s disease (PD). The inhibition of MAO-B by trans-6-styrylcoumarin is selective, highly potent, and specific, rendering it a valuable compound for future drug discovery efforts targeting MAO-B [282].

**Figure 11:**
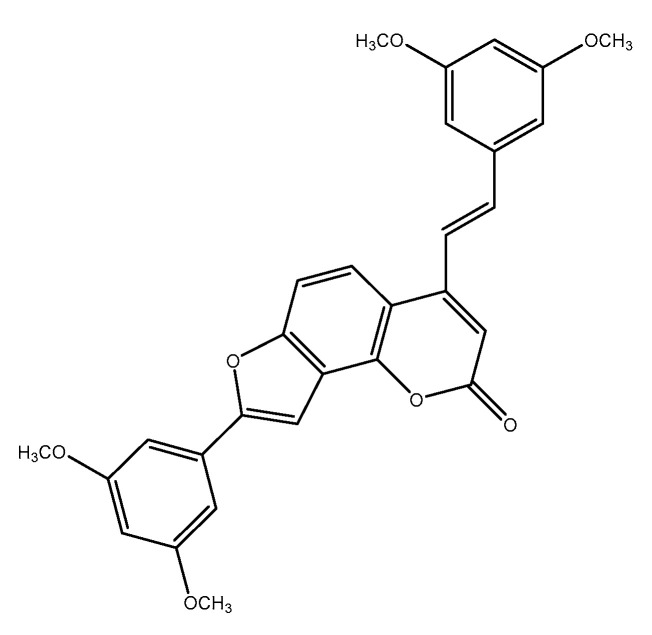
Chemical structure of Compound **4h**.

Agbo et al. (2020) carried out a study on the synthesis of furocoumarin-stilbene hybrids as potential multi-functional drugs for the treatment of AD [283]. These hybrids were synthesized and evaluated for their inhibitory effects on AChE, BChE, beta-secretase (BACE), cyclooxygenase-2 (COX-2), and lipoxygenase-5 (LOX-5) activities and their antioxidant properties. Agbo et al. (2020) demonstrated that compound **4h** (8-(3,5-dimethoxyphenyl)-4-(3,5-dimethoxystyryl)furochromen-2-one), a furocoumarin-stilbene hybrid, possesses significant anticholinesterase activity (Figure 11) [283]. Furthermore, the compound effectively inhibits the activities of β-secretase, COX-2, and LOX-5. Furthermore, compound **4h** displays robust antioxidant characteristics, as indicated by its DPPH radical scavenging capacity with an IC_50_ value of 6.8 μM. The results demonstrate that compound **4h** is effective against breast MCF-7 cancer cells and Hek293-T cells, with IC_50_ values of 1.4 ± 0.23 μM and 5.4 ± 0.62 μM, respectively. The study demonstrates that the furocoumarin-stilbene hybrids possess potential anti-Alzheimer’s effects through a range of activities. The compounds effectively inhibit AChE and BChE, which are essential enzymes for the breakdown of acetylcholine, a neurotransmitter that is crucial for memory and cognitive function. Furthermore, these hybrids display notable anticholinesterase activity. Furthermore, the hybrids demonstrated effective inhibition of β-secretase, an enzyme that plays a pivotal role in the production of Aβ peptides, which form plaques in the brains of Alzheimer’s patients. Furthermore, the compounds demonstrated the inhibition of cyclooxygenase-2 (COX-2) and lipoxygenase-5 (LOX-5) activities, which are associated with the progression of AD through the mediation of inflammation and oxidative stress. The compounds exhibited antioxidant effects by scavenging free radicals, which may potentially reduce oxidative damage in the brain. This common feature of AD was observed. These findings strongly suggest that compound **4h** has the potential to be a multifunctional drug candidate for AD treatment [283]. This study investigates the neuroprotective effects of synthetic furocoumarin-stilbene hybrids, which have the potential to serve as a treatment for AD. The hybrid compounds offer certain advantages over natural stilbenes, including higher bioavailability and the ability to cross the blood–brain barrier. The study established that these compounds exhibited potent inhibitory effects against AChE and β-secretase enzymes, in addition to possessing antioxidant properties. The mechanisms of action were elucidated through the integration of molecular modeling and kinetic studies. However, it should be noted that the majority of the data are in vitro and clinical efficacy is not yet known. The necessity for further research is indicated by the limited variety of samples available, the potential for adverse effects, and the need for exploration of bioavailability. Furthermore, it was observed that the DPPH test does not fully reflect physical conditions. Consequently, a comprehensive evaluation of the therapeutic potential of the compounds is imperative for future studies.

Mellado et al. (2022) and Agbo et al. (2020) investigate the synthesis and effects of resveratrol derivatives in the context of neurodegenerative diseases [282,283]. However, their respective approaches concentrate on disparate mechanisms and diseases. Mellado et al. (2022) developed MAO-B inhibitors for the treatment of PD. Trans-6-styrylcoumarin has been demonstrated to be highly effective in inhibiting MAO-B, exhibiting a greater degree of potency than both trans-resveratrol and 3-phenylcoumarin [282]. The interactions of the compounds with MAO-B have been subjected to analysis using advanced computational techniques. Trans-6-styrylcoumarin, a MAO-B inhibitor, has been identified as a promising candidate for the treatment of PD. In a separate study, Agbo et al. (2020) developed furocoumarin-stilbene hybrids with multiple targets for AD. The compound **4h** has been demonstrated to inhibit AChE, BChE, β-secretase, COX-2 and LOX-5 enzymes, while also exhibiting strong antioxidant properties [283]. It has the potential to be an efficacious treatment for AD by targeting multiple pathways, including ChE, Aβ aggregation and inflammation. In conclusion, Mellado et al. have concentrated their efforts on the development of MAO-B inhibitors for the treatment of PD, while Agbo et al. have focused their research on the potential of their compounds for the treatment of AD. The two studies have yielded promising results with regard to the development of compounds that may prove beneficial in the treatment of neurodegenerative disorders. Koukoulitsa et al. (2016) investigated the biological and computational evaluation of resveratrol inhibitors targeting BACE-1 as they relate to AD [145]. The researchers evaluated the inhibitory activity of various resveratrol derivatives on BACE-1, which is involved in the production of Aβ, a key factor in Alzheimer’s pathology. Notable discrepancies are evident between the BACE-1 inhibitory activities and oxytosis inhibitory activities of natural resveratrol (1) and its derived analogs. The IC50 value for BACE-1 inhibitory activity of natural resveratrol is 528 µM, while its oxytosis inhibitory activity is 54.667 mM. These values indicate that natural resveratrol exhibits a markedly lower degree of inhibitory activity in comparison to other analogs. Derived analog 11 exhibits an IC_50_ value of 3 µM for BACE-1 inhibitory activity and an EC_50_ value of 0.012 mM for oxytosis inhibitory activity. This evidence indicates that it is a markedly more efficacious inhibitor than natural resveratrol. Similarly, analog 10 has an IC_50_ of 10 µM for BACE-1 inhibitory activity and an EC50 of 0.030 mM for oxytosis inhibitory activity. Furthermore, this analog has been shown to be more efficacious than native resveratrol. Analog 5 has an IC_50_ of 18 µM for BACE-1 inhibitory activity and an EC_50_ of 0.432 mM for oxytosis inhibitory activity. These values indicate that it performs better than native resveratrol. Moreover, Analog 3 has an IC_50_ of 12 µM for BACE-1 inhibitory activity and an EC_50_ of 0.484 mM for oxytosis inhibitory activity. Additionally, these analog exhibits enhanced activity compared to native resveratrol. It can be concluded that natural resveratrol exhibits minimal inhibitory activity against BACE-1 and is therefore not an efficacious BACE-1 inhibitor. Derived analogs, in particular those designated as 10 and 11, demonstrate considerably elevated activity levels in comparison to those observed for natural resveratrol. For example, analog 11 is approximately 176 times more effective as a BACE-1 inhibitor than natural resveratrol. This indicates that structural modifications of natural resveratrol can markedly enhance the biological activity of the compound. The data demonstrate that when analogs are ranked in terms of BACE-1 inhibitory activity, analog 11 is the most effective. analog 10 is less effective than 11, yet still exhibits notable activity. analogs 5 and 3 display reduced activity compared to the other two analogs. Consequently, compared to natural resveratrol, derived analogs demonstrate enhanced efficacy as potential treatment options for AD [145].

Four compounds (**1**–**4**), with the potential to inhibit BACE 1 and AChE enzymes associated with AD, were synthesized and evaluated by Martinez (2024 [284]). The compounds were designed with caffeic acid and resveratrol derivatives and synthesized via a series of chemical reactions. The inhibitory activities of the compounds were evaluated and compared with those of known inhibitors. Moreover, the interactions of the compounds with enzymes and their similarity to existing drugs, as well as their toxicity profiles, were also analyzed. The objective of this research is to develop new treatment strategies for AD. The structure of compound **1** comprises a combination of 3,5-dihydroxybenzoic acid and 4-aminophenol. The structure of this compound comprises two hydroxyl groups and one amine group attached to a benzene ring. Compound **2** is a hybrid of caffeic acid and 2-aminophenol. Caffeic acid has a molecular structure comprising one hydroxyl group and one alkene group on a phenol ring. Compound **3** is a hybrid comprising caffeic acid and an aminoquinoline moiety. In addition to the caffeic acid structure, it contains an amino group and a quinoline ring. Compound **4** is a hybrid of caffeic acid and methoxyacridine. In addition to the caffeic acid structure, it contains a methoxy group and an acridine ring. The research indicates that the inhibitory activity of the synthesized derivatives (compounds **1**–**4**) is a crucial factor. As BACE 1 inhibitors, compounds **2**, **3** and **4**, which are hybrid derivatives of caffeic acid, demonstrated moderate to high inhibitory activity against BACE 1. These compounds exhibited significantly enhanced inhibitory activity compared to the parent components caffeic acid and chlorogenic acid. It is noteworthy that compound **4** (a caffeic acid-methoxyacridine hybrid) exhibited considerable potential, demonstrating complete inhibition of BACE 1 at a concentration of 10 μM. With regard to their inhibitory properties as AChE inhibitors, the caffeic acid derivatives (compounds **2**, **3** and **4**) exhibited inhibitory properties within the low micromolar range. The compounds under investigation were ranked according to their capacity to inhibit AChE, with compound **3**, comprising a combination of caffeic acid and an aminoquinoline moiety, emerging as the most potent. In contrast, resveratrol and its amide analog (compound **1**) exhibited only marginal potential for the inhibition of AChE. A comparison of the synthesized compounds with known inhibitors, such as LY2811376 and tacrine, revealed that the new derivatives exhibited superior inhibitory activity compared to the parent compounds, suggesting that they could potentially rival existing inhibitors. Furthermore, insights were provided regarding the molecular interactions of these compounds with the target enzymes. It was reported that compound **4** forms multiple hydrogen bonds and π-anion interactions at the active site of BACE 1 [284]. This article analyses the neuroprotective potential of synthetic stilbene derivatives and discusses the advantages and disadvantages that can be obtained by comparing these derivatives with natural stilbenes. The correlation between the structures and activities of hybrid derivatives provides a robust direction, particularly with regard to the inhibition of AD enzymes, including BACE 1 and AChE. The synergistic effects of different pharmacophores were evaluated by combining existing phenolic structures with amide and other functional groups in the design of hybrid derivatives. Such combinations allow the production of molecules with superior activity and brain-barrier cross-capacity. Nevertheless, the absence of data concerning the toxicological profiles and long-term effects of these synthetically developed derivatives constitutes a significant weakness, which may limit their clinical use. Although natural stilbenes offer the advantage of generally lower side effect profiles and long-term safety data, their biological activities are generally at lower levels. Consequently, the evaluation of hybrid derivatives as potential pharmaceuticals necessitates a thorough examination of their mechanisms of action and a comprehensive determination of their toxicity profiles. In conclusion, although the hybrid structures of synthetic stilbenes have produced remarkable results in terms of demonstrated efficacy, there is a need for the development of new strategies based on basic research as well as the development of existing natural compounds. The findings emphasize the necessity for the identification of more effective and safe treatment options, despite the challenges associated with the treatment of AD.

The studies by Koukoulitsa et al. (2016) and Martinez (2024) examine the potential of resveratrol derivatives as a treatment for AD [145,284]. The two studies examined the inhibition of BACE-1 and demonstrated that the biological activity of resveratrol can be enhanced through structural modifications. In the study by Koukoulitsa et al. (2016), it was observed that the inhibitory effect of natural resveratrol on BACE-1 was relatively weak (IC_50_ = 528 µM) [145]. However, one of the analogs, designated “Analog 11”, was identified as a 176-fold more potent inhibitor than natural resveratrol, with an IC_50_ value of 3 µM. The findings of this study illustrate that BACE-1 inhibition can be significantly augmented through structural modifications to resveratrol. In a further development, Martinez (2024) synthesized new derivatives that target both the AChE enzyme and BACE-1. Derivatives comprising a combination of caffeic acid and resveratrol have demonstrated notable efficacy in the inhibition of BACE-1 and AChE [284]. “Compound **4**” demonstrated complete inhibition of BACE-1, while “Compound **3**” exhibited the greatest efficacy in AChE inhibition. The studies conducted by Martinez et al. (2024) indicated that these hybrid compounds may have multifunctional capabilities for the treatment of AD [284]. The results of both research projects indicate that resveratrol and caffeic acid derivatives have the potential to function as enhanced therapeutic agents for AD by targeting key enzymes involved in the disease’s pathology. However, the research conducted by Koukoulitsa et al. (2016) focused more on enhancing BACE-1 inhibition [145]. Cheng et al. (2018) investigated the development of resveratrol-maltol hybrids as multi-target-directed agents for AD [285]. The 3-hydroxypyran-4-one moiety (maltol) was integrated into the structure of resveratrol to create a series of compounds (**8a–8k**) with potential therapeutic effects against AD. The compounds were evaluated for their ability to inhibit the self-induced aggregation of Aβ_1-42_, for their antioxidant properties and for their ability to chelate metals. In a study conducted by Cheng et al. (2018), it was found that compounds **8i** (E)-5-hydroxy-2-[2-(4-hydroxy-phenyl)-vinyl]-4H-pyran-4-one) and **8j** (E)-5-hydroxy-2-[2-(2-hydroxy-phenyl)-vinyl]-4H-pyran-4-one) effectively inhibited self-induced Aβ1–42 aggregations, with IC_50_ values of 7.20 μM and 8.29 μM, respectively [285]. Furthermore, these compounds demonstrated notable ABTS%+ scavenging effects, with values of 1.94% and 1.18%, and efficacious metal chelating properties. The multi-target mechanism of action of compounds **8i** and **8j** makes them suitable for the treatment of AD. The compounds under examination have been demonstrated to inhibit the aggregation of Aβ1–42 peptides, which are responsible for the formation of amyloid plaques in the brain, a defining feature of AD. Additionally, these compounds exhibit robust antioxidant properties that assist in combating oxidative stress, a pivotal factor in neurodegenerative disorders such as AD. Moreover, compounds **8i** and **8j** have the capacity to complex with metal ions, including Fe^3^⁺ and Cu^2^⁺, which are implicated in the pathology of AD. These compounds provide a comprehensive approach to addressing the complex mechanisms involved in AD, targeting Aβ aggregation, oxidative stress, and metal ion imbalance [285].

Carradori et al. (2022) examined the structure–activity relationships (SARs) of RSV derivatives (comprising compounds **1**–**9**), with a particular focus on their potential to inhibit two enzyme types, namely MAOs and carbonic anhydrases (CAs) [286]. The objective of the research was to identify the most suitable candidates for further development as potential treatments for neurodegenerative disorders. A series of RSV derivatives, designated as compounds **1**–**9**, were synthesized and evaluated for their biological activities, with a particular focus on their ability to inhibit MAOs and CAs. It is noteworthy that these compounds contain modifications in the resorcinol group of RSV_S_, specifically substitutions at positions 2 and/or 4 of the aromatic ring, which is referred to as ring A. Additionally, some compounds contain naphthyl or pyridyl moieties. Of the compounds evaluated in the study, compound **4** was identified as the most promising. This compound demonstrated low IC_50_ values of 0.43 μM for MAO-A and 0.011 μM for MAO-B. These values indicate that compound **4** is a significantly more potent inhibitor than RSV, with IC_50_ values of 13.5 μM for MAO-A and >100 μM for MAO-B. The presence of a trifluoromethyl group and a 3′-chlorine group in compound **4** resulted in a notable enhancement of inhibitory activity. Moreover, this compound displays a high MAO-B selectivity index. The number of chlorine atoms was found to exert a significant influence on the activity of other compounds, particularly compounds **1**, **2**, and **3**. However, the presence of a chlorine atom at the 3′-position had a more pronounced effect on MAO-B activity than was observed for other compounds. In comparison to compound **4**, compound **5** exhibited diminished inhibitory activity. The MAO inhibitory activities of compound **9** were less pronounced (21.3 μM for MAO-A, 11.9 μM for MAO-B). In summary, compound **4** is distinguished from the other compounds by its robust inhibitory effect on both MAO-A and MAO-B, as well as its high selectivity. Consequently, it is regarded as a prospective candidate for the treatment of neurodegenerative disorders [286]. The development of synthetic stilbenes has been motivated by the need to address the limitations of natural compounds, including their poor bioavailability and low water solubility. Nevertheless, it has been posited that hybrid derivatives, obtained through novel structural modifications, have the potential to offer enhanced neuroprotective effects in accordance with structure–activity relationships. The potential for enhancing the therapeutic efficacy of hybrid compounds in various neurological conditions is associated with a more profound understanding of the underlying mechanisms. Notably, the capacity of resveratrol and its derivatives to function as MAO-B inhibitors, their involvement in monoamine neurotransmitter metabolism, and their role in protecting against oxidative stress are particularly salient in this context. The potential for hybrid compounds to develop synergistic interactions with other natural components opens new avenues for neuroprotection. However, it is important to note that there are also some challenges in the development of synthetic stilbenes. Low bioavailability and stability problems are important factors that need to be addressed during the development phase. Furthermore, there is a necessity for increased scientific research on the long-term effects and safety profiles of synthetic derivatives. Consequently, hybrid and synthetic stilbene-based compounds are anticipated to assume a more prominent role in future neuroprotective research. While the potential advantages of synthetic stilbenes in AD treatment are significant in terms of providing a significant improvement and targeted approach compared to their natural counterparts, their development process involves some difficulties. The development of effective and safe neuroprotective treatment strategies in the future will be greatly informed by studies on structure–activity relationships.

The study by Pukasook et al. (2017) examined the design, semi-synthesis and biological evaluation of prenylated resveratrol derivatives for the potential treatment of AD [287]. The study involved the synthesis of two series of resveratrol derivatives, namely C-alkyl and O-alkyl derivatives, and an evaluation of their biological activities. The most notable findings included the identification of compound **4b** (E)-3,5,4′-trihydroxy-4-prenylstilbene as an effective Aβ aggregation inhibitor, with an IC_50_ value of 4.78 µM. Additionally, the compound exhibited antioxidant activity with an IC_50_ value of 41.22 µM, and demonstrated BACE1 inhibitory activity of 23.70% (at 50 µM). These findings indicate that compound **4b** may act as a multi-targeted agent against AD. In conclusion, the results of this study indicate that prenylated resveratrol derivatives may be effective against AD via multiple targeted mechanisms [287]. This study provides substantial evidence that prenylation can enhance the neuroprotective properties of stilbene derivatives. This comprehensive analysis elucidates the merits and limitations of these compounds as potential therapeutic agents for neurodegenerative diseases, including AD. The findings of the article demonstrate that stilbene derivatives obtained by the prenylation process exhibit increased bioactivity and versatility in comparison to natural stilbenes. Notably, compound **4b** has been observed to demonstrate remarkable inhibitory effects on Aβ aggregation, antioxidant activity, and its capacity to function as a β-secretase inhibitor. The impact of molecular modification, characterized by the presence of alkyl groups, is evident in conjunction with the retention of the fundamental stilbene structure. However, it is important to note that these studies also exhibit certain weaknesses. For instance, it has been established that certain prenylated resveratrol derivatives may possess undesirable characteristics, such as neurotoxicity. This is a matter that should be given due consideration in the context of the safety of potential therapeutic applications. Additionally, there is a possibility that in-depth mechanism studies may be lacking, which may prevent a clear understanding of the mechanisms of action of the compounds. In conclusion, this study demonstrates the potential of prenylated stilbene derivatives as multi-target agents in the treatment of AD, and also provides an important basis for understanding the relationship between the structural properties and biological activities of these compounds. Further research in this area is necessary to develop strategies to increase both the efficacy and safety of these compounds.

The two studies, by Carradori et al. (2022) and Pukasook et al. (2017), both examine the potential of resveratrol derivatives as therapeutic agents for neurodegenerative disorders, albeit through an investigation of disparate mechanisms and diseases. The two studies investigate resveratrol derivatives, yet they focus on disparate neurodegenerative disorders and mechanisms [286,287]. Carradori et al. (2022) highlights the importance of MAO inhibition in the context of neurodegeneration, with compound **4** exhibiting high selectivity for MAO-B, which is of particular relevance in the context of diseases such as Parkinson’s [286]. In contrast, Pukasook et al. (2017) focus on AD, with compound **4b** targeting Aβ aggregation and other associated mechanisms [287]. Both compounds have the potential for further development, although their therapeutic targets differ according to the disease and underlying mechanisms. Pan et al. (2014) identified a series of resveratrol derivatives as multi-targeted agents for the treatment of AD [288]. The objective of the study was to evaluate the inhibitory activities of the compounds against ChEs, with a particular focus on AChE and BChE, as well as their effects on Aβ aggregation and MAO inhibition. In the study, resveratrol analogs with a 3,5-dimethoxy-trans-stilbene structure were designed. The compounds under examination were constructed with spacers of varying lengths (2 to 8 carbons) and attached with different amine groups. Compound **6r** is a resveratrol derivative that offers a multi-targeted therapeutic approach to the treatment of AD. In comparison to other derivatives, compound **6r** demonstrated notable efficacy in addressing AD, particularly in several pivotal areas. Firstly, **6r** exhibited moderate inhibitory activity on AChE, with an IC_50_ value of 6.55 mM. The inhibitory activity on BChE was determined to be 8.04 mM, as indicated by the IC_50_ value. This suggests that 6r exerts a balanced inhibitory effect on both ChE types. Moreover, 6r exhibited a significant inhibitory effect on Aβ aggregation, with an inhibitory rate of 57.78% at a concentration of 20 mM. This property is of critical importance, as Aβ aggregation represents a prominent feature of Alzheimer’s pathology. Moreover, the inhibitory activity on MAO-A and MAO-B was also within the acceptable range, with IC_50_ values of 17.58 mM for MAO-A and 12.19 mM for MAO-B being obtained. This illustrates that **6r** employs a multifaceted strategy to address the various facets of AD. Furthermore, it was established that **6r** did not elicit any toxic effects on SH-SY5Y neuroblastoma cells at concentrations ranging from 1 to 50 mM. This indicates that it presents a favorable safety profile for potential therapeutic use. In conclusion, compound **6r** is distinguished from other synthesized derivatives by its balanced activities in inhibiting ChEs, reducing Aβ aggregation, and offering an acceptable safety profile. It is therefore considered a promising candidate for development against AD [288].

In conclusion, the results of studies on resveratrol derivatives indicate that these compounds have the potential to be used in the treatment of neurodegenerative disorders in a variety of ways. Carradori et al. (2022) focused their research on MAO inhibitors and identified several promising candidates for the treatment of neurodegenerative disorders such as PD, with compound **4** displaying particularly high selectivity against MAO-B [286]. In contrast, Pukasook et al. (2017) and Pan et al. (2014) focused their research on AD, investigating various aspects of Alzheimer’s pathology, including Aβ aggregation, antioxidant activity, and BACE1 inhibition [287,288]. Of particular note are compounds **4b** and **6r**, which have been identified as promising agents with multiple targets against AD. The design, synthesis, and biological evaluation of new resveratrol analogs were investigated by Mlakić et al. (2024) [289]. The researchers synthesized a series of compounds, consisting of heteroaromatic resveratrol analogs (compounds **1**–**22**), utilizing techniques such as Wittig and McMurry reactions. The objective of the synthesis was to introduce structural modifications with the potential to enhance biological activity. The synthesized compounds were evaluated for antioxidant activity using the DPPH and CUPRAC tests. Additionally, the capacity of the compounds to inhibit AChE and BChE enzymes was evaluated, and IC_50_ values were determined. In the DPPH test of the synthesized compounds, some analogs exhibited radical scavenging (quenching) rates in excess of 50%. For example, compound **3** exhibited the highest antioxidant activity, demonstrating a 70% scavenging effect on the DPPH radical. Moreover, the IC_50_ values were observed to be below 10 µM for multiple compounds, which is indicative of high activity. The inhibitory potential of the compounds on AChE and BChE was also evaluated, with some analogs exhibiting IC_50_ values below 5 µM. For instance, compound **7** demonstrated an IC_50_ value of 4 µM for AChE and 3 µM for BChE. These values were found to be lower than those of known inhibitors, namely trans-resveratrol and galantamine. Molecular docking studies have demonstrated that certain compounds are capable of forming three to five hydrogen bonds with the active sites of ChE enzymes. These interactions serve to enhance the efficacy of the compounds in inhibiting enzyme activity. The results indicate that these novel resveratrol analogs may represent a promising avenue for the management of neurological disorders, given their antioxidant and ChE inhibitory properties [289].

The research presented by Cheng et al. (2018) and Mlakić et al. (2024) focused on the development of resveratrol derivatives for the treatment of AD [285,289]. In their 2018 study, Cheng et al. investigated the inhibition of Aβ_1-42_ aggregation, antioxidant properties, and metal chelating abilities of resveratrol-maltol hybrids. In particular, compounds **8i** and **8j** were observed to exhibit pronounced inhibitory effects on Aβ_1-42_ aggregation and metal chelation. These hybrids offer a versatile mechanism for the treatment of AD. Mlakić et al. (2024) synthesized heteroaromatic derivatives of resveratrol and investigated the antioxidant properties and AChE and BChE inhibition of these compounds. The findings revealed that compound **7** exhibited considerable inhibitory activity against both AChE and BChE, whereas compound **3** demonstrated the highest antioxidant capacity [289]. In conclusion, Cheng et al. (2018) concentrated on the inhibition of Aβ aggregation and metal chelation, whereas Mlakić et al. (2024) focused on the inhibition of ChE and the evaluation of antioxidant activity [285,289]. The findings of both studies provide promising new compounds for further investigation as potential treatments for AD. Chen et al. (2017) discovered that certain hydroxyl-functionalized stilbenes and 2-arylbenzo[b]furans have been found to have neuroprotective effects against Aβ and glutamate-induced neurotoxicity [290]. Specifically, the efficacy of compound **5** (ethyl (E)-3-(6-((E)-3,4,5-trimethoxy styryl)benzo[d][1,3]dioxol-5-yl)acrylate), Compound **11** ((E)-ethyl 3-(2-(benzofuran-2-yl)-4, The compounds 5-dihydroxyphenylacrylate and 3-(3,4-dimethoxystyryl)-4-hydroxy-5-methoxybenzaldehyde were found to be effective. The compounds identified as neuroprotective agents are 5, 11, and 37, which are derivatives of 4-dimethoxystyryl)-4-hydroxy-5-methoxybenzaldehyde. The study revealed that treatment of cortical neurons with these compounds at a concentration of 50 μM resulted in a reduction in neuronal death induced by fAβ25−35 and glutamate. This suggests that these compounds may have potential for use in the treatment of neurodegenerative disorders. It has been observed that hydroxyl-functionalized stilbenes and 2-arylbenzo[b]furans, specifically compounds **5**, **11**, **37**, **40** and **42**, protect neurons against Aβ-mediated neurotoxicity. It has been demonstrated that compounds **5**, **11**, and **37** protect cortical neurons by reducing Aβ-mediated neurotoxicity. Of these, compound **37** was identified as the most effective in counteracting Aβ- and glutamate-mediated toxicity. The administration of compounds **5**, **11**, and **37** was observed to markedly attenuate the reductions in glial function induced by lipopolysaccharides (LPS). It was demonstrated that hydroxyl-functionalized stilbenes and 2-arylbenzo[b]furans exert anti-neuroinflammatory effects on glial cells. The study identified compounds **5**, **11**, and **37** as significant due to their neuroprotective effects against Aβ-mediated neurotoxicity and glutamate-mediated excitotoxicity. These compounds demonstrate promise in protecting cortical neurons from cell death caused by these neurotoxic stimuli. The neuroprotective effects of compounds **11** and **37** have been demonstrated in models of Aβ- and glutamate-induced toxicity. It is important to note that this assertion is based on objective evidence and not on subjective evaluation [290].

In a recent study, Firdoos et al. (2023) investigated the in silico identification and characterization of novel stilbene analogs as potential multi-target drugs for the treatment of AD [291]. The results of the study indicated that WS6 (a stilbene analog comprising a biphenyl structure with a double bond between two phenyl rings) demonstrated a robust binding affinity for both AChE and BChE, with binding energies of −10.1 kcal/mol and -7.8 kcal/mol, respectively. These values indicate that WS6 has a superior binding potential in comparison to standard drugs such as resveratrol and galanthamine. In comparative docking analyses, WS6 demonstrated a more favorable binding affinity to neurotrophin targets than resveratrol, indicating that it may possess enhanced therapeutic efficacy [291]. The advantages of synthetic stilbenes over their natural counterparts are twofold. Firstly, they exhibit superior biomolecular interaction strengths, and secondly, they offer target-oriented design opportunities. This facilitates the development of multi-target treatment strategies, with the objective of effectively addressing the various pathological mechanisms that contribute to AD. However, synthetic stilbenes also have some disadvantages. In comparison to natural stilbenes, there is a necessity for further research on the biocompatibility and side effects of certain synthetic analogs. Furthermore, the relationship between structure and activity in hybrid derivatives is generally complex, and a full understanding of these structure–activity relationships requires a clearer definition of interactions on target proteins. The present study investigates the effects of stilbenes on AChE and BChE in detail using molecular docking analyses and dynamic simulation methods. It was observed that certain synthetic stilbenes, such as WS6, exhibited significant potential with their low binding energies on AChE and BChE. Furthermore, the antioxidant and anti-inflammatory properties of these compounds are of particular significance in the context of AD treatment. Consequently, while hybrid stilbene derivatives can be evaluated as potential treatment methods for AD, further research is needed to determine the suitability and efficacy of these compounds for clinical applications.

Nagumo et al. (2019) performed a comparative analysis of stilbene and benzofuran neolignan derivatives as AChE inhibitors with neuroprotective and anti-inflammatory activities [292]. The findings demonstrate the potential of these derivatives as effective inhibitors of AChE, with promising neuroprotective and anti-inflammatory properties. The objective of the study was to assess the inhibitory impact of naturally occurring stilbene and benzofuran neolignan derivatives on the enzymatic activity of AChE. It was discovered that certain derivatives of stilbene and benzofuran neolignan possess the ability to inhibit AChE. A number of compounds, including δ-viniferin, pterostilbene trans-dehydrodimer, pallidol, grossamide and boehmenan, were observed to exert inhibitory effects on AChE activity. Furthermore, these compounds have exhibited neuroprotective properties, demonstrating the capacity to safeguard against cellular damage induced by t-BHP exposure and to inhibit NO production in vitro. The study reported the inhibitory effects of the compounds at a concentration of 12.5 μM. In particular, compounds such as δ-viniferin, pterostilbene trans-dehydrodimer, and pallidol exhibited inhibition of AChE activity in excess of 50% at this concentration. The study revealed that particular compounds derived from stilbene and benzofuran neolignans exhibited notable inhibitory activity against AChE. Grossamide and boehmenan were identified as potent inhibitors of AChE, exhibiting dose-dependent inhibition with IC_50_ values of 39.3 ± 1.2 μM and 29.8 ± 0.9 μM, respectively. Furthermore, compounds **6**, **14**, and **15** exhibited comparatively weaker inhibitory effects. It is noteworthy that the combination of two molecules of tyramine or ferulic acid resulted in an enhancement of the inhibitory efficacy of specific compounds. In the study, compound **6** is a specific benzofuran neolignan derivative that was evaluated as a racemic mixture of trans-diastereoisomers. The synthesis involved the dimerization process, which was mediated by silver (I) oxide (Ag_2_O), resulting in the formation of a dihydrobenzofuran skeleton. Compound **14** is a specific derivative that was synthesized from dimeric compound **6** through certain chemical transformations, including catalytic hydrogenation and reduction using LiAlH4. Compound **15** is another derivative that was prepared from dimeric compound **6** through specific chemical reactions, including catalytic hydrogenation and reduction using LiAlH4. The research indicated that oligomerization of plant phenolics favored AChE inhibition, suggesting a potential role in treating neurodegenerative disorders like Alzheimer’s and Parkinson’s. Notably, compounds such as pterostilbene trans-dehydrodimer, pallidol, and boehmenan were highlighted for their potential as multifunctional nutraceuticals for managing neurodegenerative disorders [292]. The present article investigates the neuroprotective potential of synthetic stilbenes, comparing them with natural stilbenes. It was asserted that synthetic derivatives offer advantages in terms of bioavailability and pharmacological activity with structural changes. Nevertheless, the costs and complexity of these processes may create difficulties in terms of marketing. The investigation further emphasized the impact of structural differences between stilbene and benzofuran neolignans on their neuroprotective activities. Oligomeric compounds have been shown to be effective as AChE inhibitors, while some synthetic derivatives have been reported to have toxic effects on the cell. Consequently, while synthetic stilbenes possess considerable potential, further research is necessary to enhance their efficacy and safety. The strengths of synthetic stilbenes include their broad spectrum of activity, while their weaknesses are associated with cost and toxicity.

Braymer et al. (2011) investigated that the effects of two stilbene derivatives, L1-α and L1-β, on Aβ aggregation and neurotoxicity in the context of AD [293]. These findings suggest that L1-β may have potential therapeutic benefits for AD. The study revealed that L1-β, which contains a dimethylamino group, demonstrated more robust interactions with the Aβ peptide, particularly in the N-terminal region and inner helix, in comparison to L1-α. Both compounds have been demonstrated to inhibit the aggregation of Aβ induced by metal ions. Nevertheless, L1-β has been demonstrated to exert a more pronounced influence on metal-induced Aβ processes and neurotoxicity in comparison to L1-a. These findings underscore the importance of subtle structural differences in small molecules, such as L1-α and L1-β, in influencing their reactivity towards metal-Aβ species and their potential therapeutic implications for AD. As reported by Braymer et al. (2011), bifunctional stilbene derivatives L1-α and L1-β were observed to be more efficacious than traditional metal chelators in regulating metal-induced Aβ aggregation processes and neurotoxicity [293]. This indicates that L1-α and L1-β may be valuable candidates for further investigation in the context of metal-induced Aβ aggregation. These compounds were observed to prevent metal-induced Aβ fibrillogenesis and generate smaller Aβ species more effectively than conventional metal chelating agents under metal-free conditions. Furthermore, it can be stated with confidence that both L1-α and L1-β possess the capability to dismantle preformed metal-mediated Aβ aggregates. This illustrates their potential to alter the configuration of metal-induced Aβ aggregates and facilitate their dissolution. It is noteworthy that L1-β demonstrated a more pronounced effect on the dissolution of preformed metal-Aβ aggregates in comparison to L1-α. The study highlights the importance of the bifunctionality of the compounds, which enhances their reactivity with metal-Aβ species. The research provides evidence that L1-α and L1-β can effectively regulate metal-induced Aβ aggregation processes, as demonstrated by inhibition and disaggregation experiments. This indicates that they have the potential to act as modulators. (L1-α does not have a dimethylamino group in its structure, while L1-β does contain this specific functional group. When exposed to copper chloride (CuCl_2_) or zinc chloride (ZnCl_2_), L1-α displayed only modest changes in its absorption spectra. In contrast, L1-β exhibited more significant alterations in its absorption spectra when interacting with both CuCl_2_ and ZnCl_2_) [293]. This study evaluates the neuroprotective potential of bifunctional synthetic stilbene derivatives designed to target metal-bound Aβ species associated with AD pathogenesis. Synthetic stilbenes have demonstrated the capacity to impede the aggregation of Aβ by forming complexes with metal ions, while the consequences of derivatives such as L1-b, which exhibit elevated bioactivity, have become evident. However, these compounds are associated with limitations, including a lack of selective activity and the potential for adverse side effects. The present study demonstrates that the structure of synthetic stilbene derivatives can influence their interactions with Aβ and neurotoxicity, thus providing a foundation for the development of more effective compounds. In conclusion, although these compounds are promising as potential AD drugs, they present certain challenges and unknowns. Consequently, further research is necessary to enhance our comprehension of their mechanisms of action.

**Figure 12:**
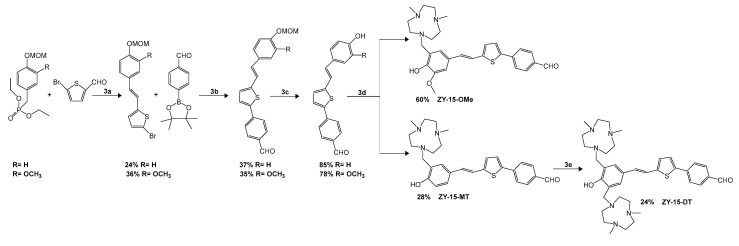
Synthetic route for the ZY-15-OMe/MT/DT amphiphilic compounds.

The studies reported by Chen et al. (2017), Firdoos et al. (2023), Nagumo et al. (2019), and Braymer et al. (2011) have concentrated on the advancement and assessment of stilbene and benzofuran derivatives as prospective therapeutic agents for the management of AD [290,291,292,293]. These compounds have been demonstrated to exert a range of neuroprotective effects, including the inhibition of Aβ aggregation, the reduction in neurotoxicity, the inhibition of AChE, and the chelation of metal ions. These effects have great consequences on the progression of AD. The findings of all studies suggest that stilbene derivatives and related compounds have the potential to be developed as multi-targeted agents for the treatment of AD, addressing different pathological processes. Chen et al. (2017) focused on the inhibition of Aβ aggregation and neuroprotection, with compounds such as Compound **37** exhibiting notable efficacy against Aβ and glutamate toxicity [290]. Firdoos et al. (2023) highlighted the potential of WS6 as a highly efficacious AChE and BChE inhibitor, exhibiting superior binding affinity in comparison to conventional pharmaceutical agents [291]. Nagumo et al. (2019) highlighted the AChE inhibitory and anti-inflammatory properties of specific stilbene and benzofuran derivatives [292]. Braymer et al. (2011) demonstrated the metal-chelating abilities of L1-α and L1-β, particularly in the context of regulating metal-induced Aβ aggregation [293]. In conclusion, the studies demonstrate the potential of stilbene derivatives and benzofuran neolignan derivatives as promising therapeutic candidates for AD, addressing key factors such as Aβ aggregation, ChE activity, oxidative stress, neuroinflammation, and metal ion imbalance. Yu et al. (2022) investigated twelve novel amphiphilic compounds with varying hydrophobic and hydrophilic fragments to target Aβ oligomers associated with AD [294]. According to the study, some compounds, such as ZY-12-OMe, exhibited cytotoxicity at a concentration of 10 mM, resulting in less than 50% cell viability. However, the study also found that at a concentration of 5 mM, the cell viability was more than 75%. Moreover, certain compounds, including ZY-12-MT, ZY-15-MT, ZY-15-OMe, ZY-17-MT, ZY-5-MT, ZY-5-DT, and ZY-5-OMe, showed no significant cytotoxicity, as indicated by >80% cell viability, even at a concentration of 10 mM. As reported by Yu et al. (2022), compounds ZY-15-MT and ZY-15-OMe were found to disrupt the interactions between Ab oligomers and human neuroblastoma SH-SY5Y cell membranes through specific mechanisms [294]. It was observed that these compounds reduced the binding of Aβ oligomers (AβO) to cell membranes, which may have been due to their interaction with the AβO themselves. The formation of hydrogen bonds and pi-pi interactions with crucial residues, including His6, Asp7, and Tyr10, resulted in the effective disruption of the affinity of AβO for cell membranes. Although the binding of Ab fibrils to cell membranes was not significantly reduced, ZY-15-MT and ZY-15-OMe (Figure 12) exhibited selectivity in alleviating the neurotoxicity of Ab oligomeric species, indicating their potential for disrupting interactions with oligomers specifically. In general, it can be stated that the compounds interact with AβO through specific residues and mechanisms, which are likely to interfere with the harmful interactions between AβO and cell membranes, thereby reducing neurotoxic effects. This indicates that the compounds may offer therapeutic benefits in the treatment of neurodegenerative disorders associated with AβO. The research findings indicate that these compounds exhibit high binding affinity to both Aβ plaques and oligomers, with six of them demonstrating selective binding towards AβO. Moreover, the compounds exhibited the capacity to label Aβ species in brain sections of transgenic AD mice, thereby indicating their potential to interact with Aβ aggregates in a biological context. Molecular docking studies yielded valuable insights into the structure-affinity relationships of the compounds, thereby elucidating their binding mechanisms to Ab structures. Four of the compounds demonstrated potential neuroprotective effects by alleviating Cu^2+^- Aβ induced toxicity in cell viability assays. Confocal fluorescence imaging studies revealed that compounds such as ZY-15-MT and ZY-15-OMe were able to disrupt interactions between AβO and cell membranes, providing a mechanism for mitigating neurotoxicity. (ZY-15-MT: This compound likely consists of a specific molecular structure where “ZY-15” represents the compound identifier or series, and “MT” could indicate a specific functional group or moiety attached to the molecule. ZY-15-OMe: Similarly, this compound may have a structure where “ZY-15” is the compound identifier, and “OMe” typically denotes a methoxy group (-OCH3) attached to the molecule) [294].

Liu et al. (2014) studied the protective effects of MStMp, a stilbene derivative, on SH-SY5Y cells against oxidative stress induced by hydrogen peroxide [295]. They investigated the impact of MStMp (2-Methoxy-5-(2,3,4-trimethoxyphenyl)styrylpyridine) on cell viability, apoptosis, and various molecular mechanisms involved in protecting neuronal cells from oxidative damage. Additionally, they examined the activities of lactate dehydrogenase, superoxide dismutase, and nitric oxide in response to MStMp treatment in their study. They found that treatment with MStMp reduced malondialdehyde (MDA) content by 34.9% (at 25 μmol/L), 39.4% (at 50 μmol/L), and 42.3% (at 100 μmol/L) compared to treatment with H_2_O_2_ alone. The activities of superoxide dismutase (SOD) were increased by 47.5% (25 μmol/L), 65.3% (50 μmol/L), and 82.7% (100 μmol/L) following treatment with MStMp. Moreover, the levels of reduced glutathione (gSH) were increased by 23.1% (25 μmol/L), 38.5% (50 μmol/L), and 47.4% (100 μmol/L) by MStMp. Furthermore, MStMp demonstrated superior inhibitory effects on nitric oxide (NO) content and inducible NOS activity compared to tetramethylpyrazine (TMP). MStMp was observed to mitigate the deleterious effects of hydrogen peroxide (H_2_O_2_) on cell viability in a concentration-dependent manner. The administration of MStMp proved to be more efficacious than tetramethylpyrazine (TMP) in safeguarding SH-SY5Y cells from H_2_O_2_-induced damage. Additionally, MStMp demonstrated superior protection against H_2_O_2_-induced reduction in cell viability and inhibition of lactate dehydrogenase (LDH) release, indicating its potential for preserving cell membrane integrity. Moreover, MStMp demonstrated the capacity to inhibit apoptosis induced by H_2_O_2_, as evidenced by a reduction in the number of apoptotic cells. The protective effects of MStMp on neuronal cells are attributed to its antioxidant properties and the inhibition of the ROS-NO pathway. MStMp has been demonstrated to reduce oxidative damage by decreasing lipid peroxidation and increasing free radical scavenging activity. Furthermore, it has been demonstrated that MStMp inhibits apoptosis induced by hydrogen peroxide (H_2_O_2_) through the inhibition of caspase-3 and caspase-9 activation, which are key markers of apoptosis. Furthermore, MStMp has been demonstrated to restore endogenous antioxidant activity and decrease lipid peroxidation in neuronal cells exposed to H_2_O_2_; thus, indicating its cytoprotective effects. In response to MStMp treatment, alterations in the activities of lactate dehydrogenase (LDH), superoxide dismutase (SOD), and nitric oxide synthetase (NOS) were observed. The administration of MStMp resulted in a notable reduction in LDH release, which suggests the preservation of cellular membrane integrity. Additionally, MStMp increased the activities of SOD and GSH, which are important antioxidant enzymes. Furthermore, MStMp decreased the content of nitric oxide (NO) by inhibiting inducible NOS (iNOS) expression and activity in a time-dependent manner, suggesting a role in modulating the ROS-NO pathway [295]. In comparison with natural stilbenes (e.g., resveratrol), synthetic stilbenes offer certain advantages for the treatment of AD due to their enhanced bioavailability and prolonged duration of action. Nevertheless, these synthetic derivatives are associated with the potential for adverse effects and toxicity, which may give rise to concerns regarding their efficacy and safety in clinical settings. The efficacy of MStMp can be enhanced by structural modifications and bioisosteric substitution methods of hybrid derivatives. An effective understanding of these mechanisms is critical to enhance the therapeutic potential of such compounds. However, the toxicological profiles of these synthetic derivatives remain comparatively understudied in relation to their natural forms. Their strengths include effective neuroprotective properties and potential therapeutic effects, while their weaknesses are limited by a paucity of data on long-term efficacy and safety. Consequently, the evaluation of hybrid stilbene derivatives in light of current research is imperative for the advancement of future drug development [295].

Azmi et al. (2021) investigated the synthesis of indolostilbenes via FeCl_3_-promoted oxidative cyclization and their biological effects on NG108-15 cell viability and H_2_O_2_-induced cytotoxicity [296]. The objective of the research was to develop a novel class of compounds, indolostilbenes, through a radical cation cyclization method utilizing FeCl₃ under mild conditions. Subsequently, the synthesized compounds were evaluated for their protective activity against H_2_O_2_-induced cytotoxicity in neuronal cells. The indolostilbenes synthesized in the study exhibited a pronounced impact on the viability of NG108-15 cells when subjected to H_2_O_2_-induced cytotoxicity. Pre-treatment with indolostilbene 8 at a concentration of 100 μM resulted in a notable increase in cell viability, with a mean value of 72 ± 1% compared to cells treated with H_2_O_2_ alone. Similarly, pre-treatment with indolostilbene 9 at a concentration of 50 μM demonstrated a significant enhancement in cell viability, with viability of 80 ± 1% observed in treated cells, compared to those treated with H_2_O_2_ alone. In conclusion, the protective effects of both indolostilbenes resulted in an increase in cell viability of between 21% and 31% when compared to cells exposed to H_2_O_2_ alone. These findings indicate that the indolostilbenes may possess neuroprotective properties, suggesting a promising avenue for further investigation into potential therapeutic applications targeting conditions associated with oxidative stress-induced cell damage. The study involved the synthesis of indolostilbene 8 and 9, which exhibit a key structural difference in the coupling positions between the indole and stilbene moieties. Indolostilbene 8 exhibits an ortho-ortho coupling, whereas indolostilbene 9 displays an ortho-para coupling. Additionally, Indolostilbene 8 forms an intramolecular hydrogen bond between the N-H amide in the stilbene moiety and the C=O amide in the indole moiety, leading to a more rigid molecular structure. On the other hand, indolostilbene 9 does not have this intramolecular hydrogen bond, which allows for greater flexibility in its molecular conformation) [296]. This article reviews the neuroprotective effects of synthetic indolostilbenes and the advantages and disadvantages of these derivatives as potential AD drugs compared to natural stilbenes. Synthetic indolostilbenes have been shown to enhance cellular survival through the augmentation of free radical scavenging and antioxidant properties, a consequence of the incorporation of indole. The limited bioavailability and stability of natural stilbenes suggests that synthetic derivatives may be a more effective alternative. However, the production costs and long-term safety profiles of synthetic compounds are among the disadvantages. Consequently, further research is necessary to determine the clinical utility of these synthetic derivatives. In conclusion, a more in-depth study of the structural diversity and mechanisms of indolostilbenes may contribute to the identification of their strengths and weaknesses and encourage the development of new strategies for the treatment of AD [296].

**Figure 13:**
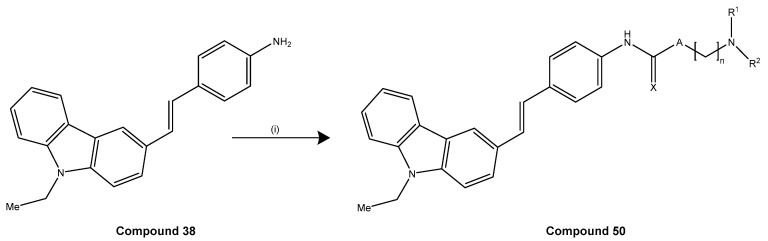
Synthetic route for the synthesis of compound (**50**).

The studies by Yu et al. (2022), Liu et al. (2014), and Azmi et al. (2021) investigated novel compounds targeting neurodegenerative disorders such as AD by examining their neuroprotective effects in cell-based models [294,295,296]. Despite the different structural types and mechanisms, all studies converge on the mitigation of oxidative stress, cell toxicity, and Aβ aggregation, which are pivotal in the pathology of AD. The three studies collectively emphasize the development of novel neuroprotective compounds that address key pathological mechanisms of AD, including Aβ aggregation, oxidative stress, and cell toxicity. The studies by Yu et al. (2022) focused on the interaction of AβO with cell membranes, whereas those by Liu et al. (2014) and Azmi et al. (2021) concentrated on the neurotoxic effects of oxidative stress [294,295,296]. These compounds demonstrate promise as potential therapeutic agents for the reduction in neurodegeneration, exhibiting a range of mechanisms and targets that are tailored to address the diverse aspects of AD pathology. Patel et al. (2020) investigated novel carbazole-stilbene hybrids as multifunctional anti-Alzheimer’s agents [297]. The compounds were designed, synthesized, and evaluated to target multiple aspects of AD, including ChE inhibition, Aβ aggregation inhibition, antioxidant properties, and metal chelation abilities. The compound (**50**), (E)-1-(4-(2-(9-ethyl-9H-carbazol-3-yl)vinyl)phenyl)-3-(2-(pyrrolidin-1-yl)ethyl)thiourea exhibits potent inhibitory activity against AChE and BChE, with IC50 values of 2.64 μM and 1.29 μM, respectively (Figure 13). Furthermore, it has been demonstrated to exert a considerable inhibitory effect on the self-mediated aggregation of Aβ1–42. The carbazole-based stilbene derivatives have been demonstrated to possess highly efficacious anti-Alzheimer’s properties. They have the capacity to inhibit ChE, prevent Aβ aggregation, exhibit antioxidant properties, and chelate metals. In AD, carbazole stilbene derivatives have been observed to exert a multifunctional mechanism of action. They have the potential to act as ChE inhibitors, targeting both AChE and BChE in order to enhance cholinergic neurotransmission. Additionally, they inhibit Aβ aggregation, thereby reducing neurotoxicity. Moreover, the derivatives exhibit antioxidant properties, whereby they scavenge ROS in order to protect neurons from oxidative damage. Some compounds have been observed to exhibit metal chelation, whereby they bind to metal ions in order to reduce the aggregation and neurotoxicity of Aβ. In conclusion, carbazole-stilbene derivatives represent a promising avenue for the development of novel AD therapies, offering the potential to target multiple pathological pathways simultaneously [297].

**Figure 14:**
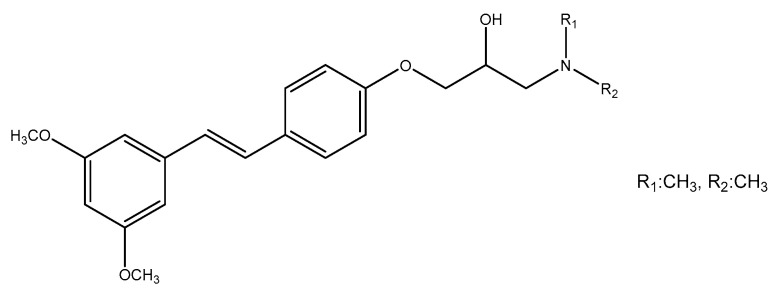
Chemical structure of compound **5f** [300].

Zheng et al. (2018) examined the design, synthesis, and evaluation of pterostilbene b-amino alcohol derivatives as multifunctional agents for the treatment of AD [298]. The derivatives were assessed for their inhibitory effects on AChE and BChE, as well as their antioxidant properties and neuroprotective effects. The study on pterostilbene b-amino alcohol derivatives for the treatment of AD revealed promising findings. The synthesized derivatives displayed selective inhibitory activity against AChE, with compound **5f** [(R,E)-1-(diethylamino)-3-(4-(3,5-dimethoxystryl)phenoxy) propane-2-ol] demonstrating the most potent inhibitory effect on EeAChE compared to pterostilbene (Figure 14). Moreover, compound **5f** exhibited notable antioxidant properties and neuroprotective effects, effectively protecting against H_2_O_2-_induced PC-12 cell injury. Additionally, it demonstrated the capacity to inhibit self-induced Aβ_1-42_ aggregation and exhibited high permeability across the blood–brain barrier in vitro. Furthermore, it was identified as a highly potent inhibitor of AChE, with an IC50 value of 24.04 μM, exhibiting substantial inhibitory activity against EeAChE. Molecular modeling studies indicate that the compound functions as a mixed-type inhibitor, simultaneously binding to the catalytic active site (CAS) and the peripheral anionic site (PAS) of AChE. Furthermore, at a concentration of 25 μM, compound **5f** was observed to exert a moderate inhibitory effect on self-induced Aβ_1-42_ aggregation, with an inhibition ratio of 40.23%. The distinctive chemical structure of compound **5f** is the primary factor contributing to its superior inhibitory activity in comparison to other derivatives. The study revealed that compound **5f**, a derivative of pterostilbene β-amino alcohol, demonstrated superior efficacy in the treatment of AD compared to pterostilbene alone. The derivatives, including compound **5f**, demonstrated robust inhibitory activity against AChE, whereas pterostilbene exhibited minimal inhibitory potency when administered alone. Moreover, the derivatives display multifunctional properties, including antioxidant activity, neuroprotective effects, and the capacity to inhibit self-induced Aβ_1-42_ aggregation. These attributes are vital for the effective treatment of AD. Compound **5f** demonstrated high blood–brain barrier permeability in vitro, indicating its potential to penetrate the central nervous system effectively. The analysis of the structure–activity relationship indicates that including the b-amino alcohol group in the derivatives could enhance their therapeutic potential for treating AD. The derivatives were found to be moderate inhibitors of EeAChE, with selectivity towards AChE over BChE. Molecular modeling studies provided insights into the potential binding mechanisms of the compounds to AChE. The results indicate that pterostilbene derivatives possess multifunctional properties and selectivity towards AchE, which suggests their potential as novel drugs for the treatment of AD [298]. This article assesses the potential use of pterostilbene β-amino alcohol derivatives in the treatment of AD. Compound **5f** is of particular interest due to its AChE inhibitory and neuroprotective properties. These synthetic derivatives have the potential to interact with multiple biomolecules, offering a targeted design advantage over natural stilbenes. Structure–activity relationships have been identified, highlighting the mechanisms by which the alcohol group enhances interaction with AChE by hydrogen bonding. The strengths of this compound include significant neuroprotective effects and specific interactions, while its weaknesses include lack of clinical efficacy and side effects. In conclusion, although these derivatives may contribute to the development of new strategies in the treatment of AD, further research and clinical studies are required.

**Figure 15:**
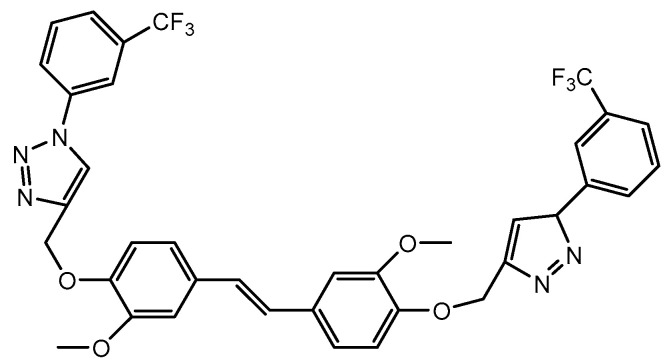
Chemical structure of 3,3′-dimethoxy-4,4′-dihydroxy-stilbene triazole (DMDHSB).

The studies by Patel et al. (2020) and Zheng et al. (2018) both focus on the synthesis of multifunctional compounds designed to target multiple pathological pathways involved in AD [297,298]. Notwithstanding the disparate structural backbones employed, namely carbazole-stilbene hybrids in the Patel study and pterostilbene β-amino alcohol derivatives in the Zheng study, both sets of compounds exhibit a range of therapeutic effects, including ChE inhibition, Aβ aggregation inhibition, antioxidant properties, and neuroprotective effects. The objective of both studies is to develop multifunctional agents that can target various aspects of AD. The carbazole-stilbene hybrids described by Patel et al. (2020) have been observed to exhibit enhanced ChE inhibition, particularly against both AChE and BChE, in addition to metal chelation and Aβ aggregation inhibition [297]. The pterostilbene β-amino alcohol derivatives from Zheng et al. (2018), particularly compound **5f**, have been observed to demonstrate selective AChE inhibition, antioxidant properties, and neuroprotection with notable blood–brain barrier permeability [298]. Both sets of compounds offer promising potential for the development of novel Alzheimer’s treatments, although they emphasize different mechanisms and structural designs in achieving their multifunctionality. Li et al. (2023) evaluated the potential neuroprotective effects of synthesized 3,3′-dimethoxy-4,4′-dihydroxystilbene triazole (DMDHSB) against Aβ-induced neuronal damage in PC12 cells (Figure 15) [299]. The study demonstrated that DMDHSB has the capacity to reverse the up-regulation of iNOS production induced by Aβ in a dose-dependent manner. Furthermore, the study investigated alterations in cell viability, PGE2 expression, and nitric oxide synthesis in PC12 cells treated with DMDHSB and Aβ. The exposure of PC12 cells to DMDHSB resulted in a significant dose-dependent reversal of the loss of viability induced by Aβ, as demonstrated by Li et al. (2023) [299]. Furthermore, the overproduction of PGE2, COX-2, NO and iNOS in PC12 cells induced by Aβ was effectively inhibited by DMDHSB. Furthermore, DMDHSB exposure prevented the Aβ treatment-associated increase in NF-κB nuclear translocation, indicating that DMDHSB may offer potential therapeutic benefits for the treatment of Aβ-induced neurodegenerative disorders. At a concentration of 8 μM, DMDHSB was observed to fully reverse the loss of PC12 cell viability associated with Aβ incubation. Furthermore, exposure to 8 μM of DMDHSB was observed to effectively suppress the up-regulation of PGE2 production promoted by Aβ, resulting in a level of production that was close to that observed in control PC12 cells. Furthermore, DMDHSB was observed to effectively reverse the dose-dependent up-regulation of COX-2 expression promoted by Aβ. At a concentration of 8 μM, DMDHSB was found to suppress COX-2 expression to a level approaching that of control PC12 cells. The results suggest that DMDHSB has the potential to reverse the inflammatory damage caused by Aβ in PC12 cells in a dose-dependent manner. As proposed by Li et al. (2023), DMDHSB may serve as a promising therapeutic agent for AD. These findings suggest that DMDHSB could be a promising candidate for further research and development in the treatment of AD [299]. The compound has demonstrated neuroprotective effects by targeting inflammatory responses associated with Aβ-induced neuronal apoptosis. It has been shown to effectively reverse the loss of viability in PC12 cells, inhibit the overproduction of inflammatory mediators such as PGE2, COX-2, iNOS, and NO, and prevent NF-κB nuclear translocation [299].

The effects of bifunctional stilbene compounds on AβO and the modulation of toxicity caused by these oligomers were investigated by Hilt et al. (2022) [300]. The objective of the research was to gain insight into the potential impact of these compounds on the conformational toxicity of AβO and the oxidative stress it induces. The compounds synthesized in the study are designated as stilbene-nitroxyl hybrid compounds. These compounds represent a novel class of molecules with the potential to modulate the toxicity and structure of AβO. In particular, various stilbene compounds were highlighted, including PMT-301, PMT-302, PMT-303, PMT-401 and PMT-402. The ability of these compounds to inhibit the inflammatory response and gene expression triggered by TGRL lipolysis products in HBMECs was investigated. The study offers valuable insights into the potential of these compounds as therapeutic agents for neurodegenerative disorders, particularly in terms of their structural properties and biological activities. The aim of this study was to examine the interactions between stilbene-nitroxyl hybrid compounds and AβO, as well as the biological activities of these compounds. The study employed a total of five distinct stilbene compounds. The compounds under investigation were designated PMT-401, PMT-402, PMT-301, PMT-302 and PMT-303. The observed effects on AβO differed between the compounds under investigation. The findings revealed that PMT-402 demonstrated the greatest potential for interaction with AβO, while PMT-401 exhibited the lowest potential. The low solubility of PMT-401 may have resulted in a reduction in its effective concentration. PMT-401 was observed to exert a pronounced inhibitory effect on the gene expression of ATF3, E-selectin, IL-6, IL-8, and COX-2, which were induced by TGRL lipolysis products. The expression of these genes was found to be reduced by 50% in the presence of PMT-401. An examination of the size distribution profiles of stilbene compounds revealed that PMT-402 and PMT-303 exhibited the lowest full width at half maximum (FWHM) values, which was associated with more effective bioactivity. It was demonstrated that TL elevated the transcription of stress response factors and pro-inflammatory genes in HBMECs. The effect of TL was observed to result in a 30–40% increase at the mRNA level. These findings demonstrate the potential of stilbene compounds to modulate the toxicity of AβO and illustrate their potential utility in the treatment of neurodegenerative disorders [300]. The structural and bioactive properties of hybrid stilbenes are associated with their capacity to mitigate the toxicity of AβO and to contend with oxidative stress. The multi-target activity of these synthetic molecules allows for intervention in the complex etiology of AD. Their strengths include the molecular study of these molecules and their structural analysis using biophysical tools, while their weaknesses include the lack of in vivo evidence and the lack of detailed understanding of mechanisms. Consequently, while synthetic stilbenes show promise in the treatment of AD, further clinical and mechanism-oriented studies are required to fully evaluate their potential.

**Figure 16:**
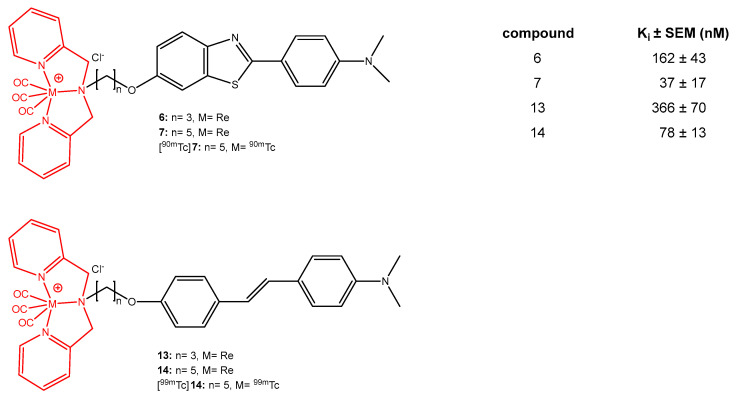
The Ki values against Aβ_1-42_ aggregates have been determined for rhenium complexes 6, 7, 13 and 14.

The studies by Li et al. (2023) and Hilt et al. (2022) both investigate the potential of stilbene-based compounds for the treatment of neurodegenerative disorders, particularly AD [299,300]. The two studies investigate the modulatory effects of these compounds on inflammatory responses and Aβ-induced toxicity, although they employ different approaches with regard to the specific compounds used and the mechanisms targeted. In their study, Li et al. (2023) investigated the potential neuroprotective effects of the synthesized compound DMDHSB (3,3′-dimethoxy-4,4′-dihydroxystilbene triazole) against Aβ-induced damage in PC12 cells [299]. The study examined the manner in which DMDHSB counteracts the inflammatory response and neuronal damage caused by Aβ. This entailed an investigation of the upregulation of iNOS, COX-2, and PGE2 production, as well as the prevention of NF-κB nuclear translocation. In a separate study, Hilt et al. (2022) investigated the impact of stilbene-nitroxyl hybrid compounds on AβO, which are highly toxic and a primary contributor to AD progression [300]. The capacity of compounds such as PMT-401, PMT-402, PMT-301, PMT-302, and PMT-303 to modulate the conformational toxicity of Aβ was evaluated. Moreover, they inhibit the expression of inflammatory genes in human brain microvascular endothelial cells (HBMECs) induced by toxic lipolytic products. The two studies focus on stilbene-based compounds and their neuroprotective potential for the treatment of AD, although they emphasize different mechanisms. Li et al. (2023) highlight the anti-inflammatory and anti-apoptotic effects of DMDHSB, particularly in reversing Aβ-induced neuronal damage in PC12 cells [299]. Hilt et al. (2022) focuses on the modulation of AβO and the reduction in endothelial inflammation through stilbene-nitroxyl hybrids, with a particular emphasis on PMT-402 [300]. Both sets of compounds demonstrate promise in addressing Aβ-related toxicity and inflammation, making them valuable candidates for further research in AD treatment. Jia et al. (2014) is aimed at developing and evaluating imaging agents for selective targeting of Ab plaques in the blood vessels of the brain [301]. The study examines the potential of rhenium complexes 6, 7, 13, and 14 as imaging agents for targeting Ab plaques in cerebral amyloid angiopathy using positively charged 99m Tc/Re-labeled benzothiazole and stilbene derivatives. The present study examines the potential of rhenium complexes 6, 7, 13, and 14 as imaging agents for targeting Aβ plaques in cerebral amyloid angiopathy. These complexes represent a series of 99m Tc-labeled benzothiazole and stilbene derivatives that carry a positive charge. In vitro binding studies were conducted to assess the affinity of rhenium complexes 6, 7, 13, and 14 for Aβ (1–42) aggregates. The study revealed that the rhenium complexes 6, 7, 13, and 14 demonstrated a moderate affinity for Aβ_1-42_ aggregates, with Ki values ranging from 37 to 366 nM. Among the complexes under examination, complex 7 exhibited the highest binding affinity, with a Ki value of 37 nM, while complex 14 demonstrated a Ki value of 78 nM. The affinity of complex 6 was moderate, with a Ki value of 162 nM, while complex 13 displayed the lowest binding affinity, with a Ki value of 366 Nm (Figure 16). These discrepancies in binding affinity indicate varying degrees of interaction and specificity of these rhenium complexes with Ab aggregates. Furthermore, in vitro fluorescent staining of compounds **7** and **14** on brain sections of AD patients demonstrated intense labeling of Aβ plaques in proximity to blood vessels. The initial brain uptake of [99m Tc]7 and [99m Tc]14 is relatively low, and their fast blood washout suggests that they may be suitable as SPECT imaging agents for the selective detection of Ab plaques in cerebral vessels. Moreover, the study demonstrated that the imaging agents were unable to traverse the intact blood–brain barrier, thereby enabling the selective labeling of Ab plaques on cerebral blood vessel walls. This finding demonstrates the potential of the agents for targeted imaging of Aβ plaques in the brain’s blood vessels with high specificity and accuracy [301].

Ono et al. (2003) investigated the development and evaluation of C-labeled stilbene derivatives as PET imaging agents for targeting amyloid plaques in AD [302]. A series of stilbene derivatives was synthesized and their binding affinities towards A-aggregates were assessed in vitro. The study illustrates the potential of these compounds as positron emission tomography (PET) tracers for imaging A-aggregates in the brains of patients with AD. The 4-N,N-dimethylamino-4′-methyoxy stilbene demonstrated a Ki value of 6.0 ± 1.5 nM, whereas the 4-N-monomethylamino-, 4′-hydroxy stilbene exhibited a Ki value of 1.3 ± 0.4 nM towards A-aggregates in vitro. The 4-N,N-dimethylamino-4′-methyoxy and 4-N-monomethylamino-, 4′-hydroxy stilbenes exhibited favorable binding affinities towards A-aggregates in vitro, with Ki values less than 10 nM. In particular, compound **4** exhibited a Ki value of 6 nM, thereby establishing its potential as a promising candidate for further testing and C-11 labeling as a PET imaging agent for A-aggregates in AD research. The synthesized stilbene derivatives demonstrate a high affinity for A-aggregates, particularly Aβ_1-40_ aggregates, which are linked to AD pathology. The compound 4-N-monomethylamino-, 4′-hydroxy stilbene has been demonstrated to exhibit a robust binding affinity to A-aggregates, with a Ki value of 1.3 ± 0.4 nM. This stilbene derivative, along with other compounds in the series, has the potential to serve as an effective positron emission tomography (PET) imaging agent for mapping Aβ plaques in the brains of AD patients [302]. According to the article, synthetic derivatives have the potential to increase binding affinity to Aβ plaques by allowing for structural modifications. Nevertheless, disadvantages such as high lipophilicity may lead to undesirable side effects and further research is required to elucidate the mechanisms of these compounds. The establishment of a relationship between the structures and activities of hybrid derivatives may offer significant opportunities for the development of new therapeutic strategies. The strengths of this body of research include robust experimental data, while its weaknesses include limited in vivo data and the need for deeper analysis of structure–activity relationships. In conclusion, although synthetic stilbenes appear to be a promising avenue for the treatment of AD, further research is required.

**Figure 17:**
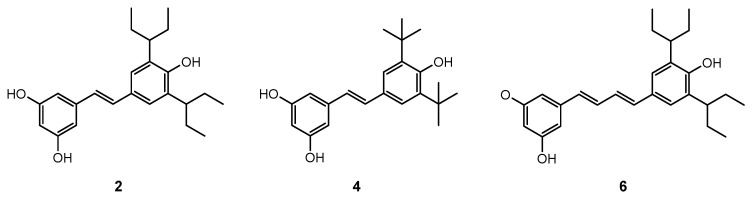
Novel neuroprotective hydroxystilbenoid derivatives.

Villalonga-Barber et al. (2011) studied the synthesis and evaluation of new hydroxystilbenoid derivatives with neuroprotective activity [303]. The objective of the research was to assess the neuroprotective properties of these compounds using glutamate-challenged HT22 hippocampal neurons as a model for oxidative stress-induced neuronal cell death. The objective of the study was to identify compounds that could provide enhanced neuroprotection without exhibiting cytotoxicity or interfering with estrogen and aryl hydrocarbon receptor-mediated transcription. In their study, Villalonga-Barber et al. identified that certain hydroxystilbenoid derivatives demonstrated notable neuroprotective efficacy against oxidative stress-induced neuronal cell death. Among the synthesized compounds, three were identified as being particularly potent. The EC50 value for compound **2** (5-{(E)-2-[3,5-Bis(1-ethylpropyl)-4-hydroxyphenyl]ethenyl}-1,3-benzenediol) was 30 nM. The EC50 value for compound **4** (5-{(1E,3E)-4-[3,5-dimethoxyphenyl]-1,3-butadienyl}-1, The EC50 value for 3-bis(1-ethylpropyl)-2-methoxybenzene was 45 nM, while that for Compound **6** (5-{(1E,3E)-4-[3,5-bis(1-ethyl propyl)-4-hydroxyphenyl]-1,3-butadienyl}-1,3-benzenediol) was 12 nM, representing the highest potency (Figure 17). The results demonstrate that these derivatives have the potential to serve as an effective neuroprotective agent against damage caused by oxidative stress. In particular, Compound **6** exhibited the most pronounced neuroprotective efficacy among the tested compounds. The numerical values provide concrete evidence of the efficacy of the compounds in safeguarding neurons under conditions of oxidative stress, suggesting their potential as candidates for the development of neuroprotective therapies [303]. In the following article, an evaluation is provided of the neuroprotective activities of the recently synthesized hydroxystilbene derivatives, and a comparison is made of the advantages and disadvantages of the derivatives in the treatment of AD with those of natural stilbenes. The derivatives exhibit 100 to 400 times stronger neuroprotective effects in comparison to resveratrol, whilst demonstrating no interaction with estrogen and aryl hydrocarbon receptors, thereby reducing the risk of hormonal cancer. Their lipophilic properties facilitate their targeting of ROS production in mitochondria. Further investigation into their in vivo activities and potential toxicity profiles is warranted. The ineffectiveness of certain compounds underscores the necessity for the optimization of these structures.

The studies by Jia et al. (2014), Ono et al. (2003), and Villalonga-Barber et al. (2011) each focus on the development and evaluation of stilbene derivatives or related compounds [301,302,303]. However, the objectives and methodologies employed by these studies vary, reflecting disparate approaches to addressing neurodegenerative disorders. In particular, AD has been the subject of investigation. Jia et al. (2014) and Ono et al. (2003) both investigated the potential of imaging agents for the detection of Aβ plaques [301,302]. However, Jia et al. focused their attention on SPECT imaging of cerebral amyloid angiopathy, whereas Ono et al. (2003) concentrated their efforts on the development of PET tracers for brain imaging [301,302]. The stilbene derivatives investigated by Ono et al. (2003) demonstrated superior binding affinities in comparison to the rhenium complexes explored by Jia et al. (2014) [301,302]. In contrast to the aforementioned studies, Villalonga-Barber et al. (2011) concentrated on the neuroprotective characteristics of hydroxystilbenoid derivatives, which have the potential to be employed in the treatment of oxidative stress, a common feature of AD and other neurodegenerative disorders [303]. All three studies emphasize the potential value of stilbene derivatives in AD research, whether for imaging or neuroprotection. Each set of compounds has been designed to address specific aspects of AD pathology. Cui et al. (2014) reported the development and evaluation of carbon-11-labeled stilbene derivatives obtained from natural products as potential imaging agents for the detection of Aβ plaques associated with AD [304]. The investigation examined four stilbene derivatives for their capacity to bind to Aβ aggregates, with a particular emphasis on (E)-1-methoxy-4-stylylbenzene (compound **8**). The primary findings regarding the binding affinity of stilbene derivatives to Aβ aggregates were as follows: the methylated ligand (E)-1-methoxy-4-stylylbenzene (compound **8**) demonstrated a high binding affinity to Aβ_1-42_ aggregates, with an inhibition constant (Ki) value of 19.5 nM. This suggests that compound **8** has a robust interaction with Aβ aggregates. The binding affinities of other stilbene derivatives (5, 6, and 7) were also assessed. However, the glucose-containing derivatives 5 and 6 exhibited markedly reduced binding affinities, with Ki values exceeding 10,000 nM and 7355 nM, respectively. This suggests that modifications to the chemical structure of these compounds reduce their ability to interact with Aβ aggregates. The in vitro neuropathological staining of a transgenic mouse model corroborated the observation that both ligands 7 and 8 exhibited marked staining of Aβ plaques and demonstrated specific binding with minimal background staining. In conclusion, the results demonstrate the potential of compound **8** as a selective and high-affinity ligand for Aβ imaging, and illustrate that structural modifications can markedly influence binding affinity. In vitro binding studies were designed to evaluate the binding affinities of these stilbene derivatives to Aβ_1-42_ aggregates. The results demonstrated that compound **8** exhibited a high degree of affinity. Furthermore, in vivo biodistribution studies examined the pharmacokinetic properties of the carbon-11 labeled ligand [11C]8 in normal mice, exhibiting favorable properties such as brain uptake and rapid clearance. In conclusion, the results of the study indicate that [11C]8 can be used to specifically label Aβ plaques in transgenic mouse models, suggesting that it may be a suitable tracer for imaging Aβ plaques in living individuals [304].

The studies concentrate on the synthesis, biological assessment, and computational analysis of an array of stilbene derivatives and resveratrol analogs, with the objective of developing MTDLs for the treatment of AD and other neurodegenerative disorders. The objective of these compounds is to address multiple pathological mechanisms of AD, including Aβ aggregation, oxidative stress, ChE inhibition, and metal ion dysregulation, which are critical in disease progression. The capacity of resveratrol derivatives to impede the self-aggregation of Aβ_1-42_, a process that culminates in the formation of amyloid plaques, a defining feature of AD, was assessed in numerous instances. The compounds **8i** and **8j** [285] demonstrated strong inhibitory activity with IC50 values of 7.20 μM and 8.29 μM, respectively. This evidence supports the proposition that structural modifications to resveratrol can significantly enhance its anti-amyloidogenic properties. A number of studies have concentrated on the inhibition of AChE and BChE, given that these enzymes are responsible for the breakdown of acetylcholine, a neurotransmitter that plays a vital role in memory and cognitive function. In particular, Mlakić et al. (2024) reported that compound **7** exhibited potent AChE and BChE inhibition with IC50 values of 4 µM and 3 µM, respectively, surpassing the activity of known ChE inhibitors such as galantamine and trans-resveratrol [285]. This highlights the potential of these derivatives as a promising class of ChE inhibitors for the treatment of AD. Moreover, a number of the derivatives were assessed for their antioxidant properties, which are of paramount importance in combating oxidative stress, a significant contributor to neuronal damage in AD. The DPPH and ABTS tests were frequently employed for the purpose of measuring the capacity of the compounds to scavenge free radicals. Compounds such as compound **3** [289] demonstrated robust antioxidant activity, with a 70% DPPH radical scavenging effect, indicating their capacity to neutralize ROS and safeguard neurons from oxidative damage. Metal ion dysregulation, particularly involving Fe^3^⁺ and Cu^2^⁺, has been demonstrated to play a role in the promotion of Aβ aggregation and oxidative stress in AD. It was demonstrated that compounds such as **8i** and **8j** were capable of effectively chelating the relevant metal ions, thereby preventing them from catalyzing the formation of toxic free radicals and amyloid plaques. This additional therapeutic potential is derived from the metal chelation property, which is capable of mitigating the neurotoxic effects of metal ion imbalances in the AD brain [285].

In several studies, molecular docking and computational techniques were employed to assess the interaction of these compounds with target enzymes, including ChE and BACE-1. These studies provided insights into the binding affinities and molecular interactions, including the formation of hydrogen bonds and π-anion interactions, which enhance the inhibitory potency of these compounds. For example, compound **4** from Martinez et al. (2024) demonstrated multiple interactions with the active site of BACE-1, indicating its potential as a potent BACE-1 inhibitor [284]. The resveratrol and stilbene derivatives under investigation provide a multifaceted approach to addressing the complex pathogenesis of AD. By targeting Aβ aggregation, ChE inhibition, antioxidant defenses, and metal ion chelation, these compounds offer a comprehensive therapeutic strategy. The findings of these studies illustrate that structural modifications to natural resveratrol can significantly enhance its biological activity, thereby rendering it a more efficacious candidate for the treatment of AD. In conclusion, these derivatives demonstrate considerable potential as multifunctional agents for the treatment of AD. The capacity to inhibit amyloid aggregation, prevent ChE activity, scavenge free radicals, and chelate toxic metal ions indicates that these compounds could potentially impede the progression of AD. It is recommended that future research should focus on optimizing these compounds for improved bioavailability, blood–brain barrier penetration, and long-term safety, in order to fully realize their therapeutic potential in clinical applications.

Table 5 summarizes the described compounds and their putative modes of action.

## 9. Nanotechnology-Based Systems for Stilbenes Delivery on Neurodegenerative Disorders

Nanotechnology is one of the methods that affect human life in different ways and is an important approach that helps overcome the multiple limitations of various diseases, especially neurodegenerative disorders. Encapsulation technology allows making active compounds more soluble, slowing down their degradation, reducing and/or eliminating their toxicity, and controlling their absorption and the emergence of their biological activity [305].

Although stilbenes are structures with a wide spectrum of action, some of their properties such as sensitivity to physiological conditions, low solubility, poor permeability, instability, and low bioavailability, limit their benefits in clinical applications [306,307,308]. Nanoformulating stilbenes are a potential alternative to overcome these challenges, thus extending their circulation time, increasing their delivery to the target site, and effectiveness [305].

There are some limitations in drug delivery to the CNS, one of which is the blood–brain barrier (BBB), which is the main disadvantage in the treatment of neurodegenerative disorders [308]. Previous studies in the treatment of neurodegenerative disorders support the use of nanotechnology that allows bioactive substances such as stilbenes to cross the blood–brain barrier, some plant-based nanosystems, and even targeted systems alone or in combination with other drugs.

Resveratrol is the most remarkable compound of the stilbene class and has promising therapeutic potential for a wide range of neuropsychiatric and neurological conditions. However, rapid metabolism, poor solubility, and low bioavailability are the main disadvantages of resveratrol that need to be overcome [307,309,310,311,312]. Resveratrol is classified in the biopharmaceutical classification system as a class II drug, which has low solubility and high permeability and whose bioavailability is limited by dissolution rates [313].

In the literature, resveratrol, whose effectiveness is increased in the treatment of neurodegenerative disorders by nanoformulation to overcome these disadvantages, has been identified as the main compound of the stilbene class. One of them, it was aimed to develop a targeted therapeutic system for intravenous administration of resveratrol and grape skin and seed extracts, solid lipid nanoparticles (SLNs) functionalized with an antibody (anti-transferrin receptor monoclonal antibody (OX26 mAb)) have been developed for the inhibitory effect of resveratrol and grape skin and seed extracts on the aggregation of Aβ, which is associated with the formation of neuritic plaques, which are pathological hallmarks of AD. Cellular uptake of SLNs, thought to be a possible carrier for transport to target the brain, has been shown to be significantly more efficient than normal SLNs and SLNs functionalized with a nonspecific antibody in experiments on human brain-like endothelial cells. Also, when resveratrol and grape extract were encapsulated, a 26% and 31% reduction in peptide aggregation, respectively, was observed after 3 days of incubation. It was concluded that SLNs can release beneficial compounds in a controlled manner by preventing Aβ aggregation [314].

Frozza et al. developed a lipid-core nanocapsule formulation to stabilize resveratrol, protect its biological activities, and increase its bioavailability. They achieved significantly higher resveratrol concentrations in the brains of healthy rats treated with nanocapsules compared to rats treated with unformulated resveratrol [315]. In their follow-up work, researchers compared the neuroprotective effects of free resveratrol treatment with those of resveratrol-loaded lipid core nanocapsule treatment versus intracerebroventricular injection of Aβ_1-42_ in rats. Animals vaccinated with Aβ_1-42_ showed a significant impairment in learning memory ability and a parallel significant decrease in hippocampal synaptophysin levels. At the end of the study, it was determined that resveratrol managed to save the harmful effects of Aβ_1-42_ using lipid core nanocapsules, while treatment with resveratrol offered only partial beneficial effects. Results can be explained by the fact that lipid core nanocapsules provide a strong increase in resveratrol concentration in brain tissue. The data not only confirms the potential of resveratrol in the treatment of AD but also appears to offer an effective way to increase the effectiveness of resveratrol via the use of nanodrug delivery systems [316].

Resveratrol was reported to protect different cell types against Aβ toxicity by scavenging ROS and inactivating caspase-3. To increase the effectiveness of resveratrol in this aspect, nanoparticles were developed and characterized using poly-caprolactone (PCL) as the hydrophobic core of the block copolymer and polyethyleneglycol as the hydrophilic shell by Lu et al. Cell viability, analysis of intracellular ROS level, and caspase-3 activity were performed in comparison with free resveratrol. It was found that 48 h incubation of resveratrol at concentrations of 5 and 10 μM significantly attenuated the viability of PC12 cells, but 12 h pre-incubation of resveratrol did not protect PC12 cells against Aβ exposure. While 48 h incubation of nanoparticles containing similar concentrations of resveratrol did not show toxicity to cells, 12 h pre-incubation protected PC12 cells from Aβ-induced damage and reduced intracellular oxidative stress and caspase-3 activity in a dose-dependent manner [317].

Sun et al. developed a new mesoporous nano-selenium (MSe) system based on the borneol (Bor) target, β-cyclodextrin nanovalves (Fc-β-CD) with loaded resveratrol, stands out with its controlled release and high targeting ability which are the main factors associated with the success of AD drugs. The system inhibited the aggregation of Aβ, decreased oxidative stress, and repressed tau hyperphosphorylation, and also successfully improved memory impairment by protecting nerve cells in APP/PS1 mice. It has been stated that MSe-Res/Fc-β-CD/Bor is an effective, safe therapeutic system for AD and a carrier that can improve the targeted delivery of drugs [183].

To evaluate the neuroprotective effect of resveratrol, nanoparticles formulated with selenium, an essential micronutrient for the brain, were used to determine neurochemical and neurochemical effects associated with aluminum chloride-induced AD model in rats at a dose of 100 mg/kg/day for 60 days. Its effect on histopathological approaches was evaluated. With nanoparticle administration, improvements in impaired oxidative markers and mitochondrial dysfunction were observed, clearance of Aβ was possible, and neuroinflammation in AD was alleviated. The results demonstrated that nanoformulation of resveratrol with selenium maximized the therapeutic potential of resveratrol against AD not only through antioxidant effect but also anti-inflammatory effect by modulating signaling pathways and improving neurocognitive function [318].

In another similar study, selenium nanoparticles of resveratrol, which have low bioavailability and solubility, were prepared to increase their ability to inhibit Aβ42 aggregation. As a result of in vitro evaluation by combining the unique Aβ absorption property of selenium nanoparticles (SeNP) with natural antioxidant properties of resveratrol, modification of resveratrol with SeNP was found to be effective compared to unformulated resveratrol in reducing Aβ42 toxicity in long-term use [319].

Resveratrol is also known for its effect on improving the function of brain cells in acquiring new things or learning, against the degeneration of brain cells that results in memory loss in AD. Its rapid degradation poses a significant problem when applied internally as a nutraceutical, and nanoemulsion formulation of resveratrol was prepared to overcome problems such as water solubility, chemical instability, and bioavailability. Formulated resveratrol was proven effective when exposed to ultraviolet rays and other sources of degradation and was physically stable at different temperatures (30, 40, and 45 °C) during storage. The strong effect of nanoemulsion-based resveratrol extracted from grape seed oil was revealed [320].

Bilosomes are carrier systems that prevent drugs from being broken down in the stomach and allow oral drug delivery as an alternative to injectable therapy. They contribute to higher biocompatibility and solubility enhancers due to their stability in the gastrointestinal tract [321]. Abbas and coworkers developed resveratrol-loaded bilosomes for oral use in order to increase resveratrol bioavailability. After in vitro characterization studies evaluating different formulation variables of resveratrol-loaded bilosomes prepared using the thin film hydration technique, the optimum formulation with the particle size of 189 ± 2.14 nm, PDI of 0.116 and encapsulation efficiency of 76.2 ± 1.36% was determined. In vivo studies on the streptozotocin-induced AD animal model demonstrated the superiority of bilosomes compared to traditional drug suspension in the memory improvement of mice evaluated through Y-maze and Morris water maze tests. In addition, a decrease in oxidative stress markers (COX-2, IL-6) and Tau and Aβ levels, which play an important role in AD, was detected in mice treated with the optimized bilosomal formulation compared to suspension [322].

Nasal drug delivery provides a remarkable route, noninvasive, and alternative technique for delivery to the brain by bypassing the BBB and directly targeting the CNS. This is an important potential application for surface-modified drug nanocarriers that can also actively cross the BBB. Drug delivery in the treatment of various diseases affecting the CNS [313]. One of the studies was conducted with intranasal application, an in situ gel-based formulation containing the resveratrol-loaded nanostructured lipid carrier developed and characterized, and then a higher drug distribution in the brain was seen with in situ gel pharmacokinetic study. The results showing the safety and effectiveness of nasal administration of the developed formulation led to the conclusion that the intranasal route may be a promising approach for the treatment of AD [323].

In the study developing the nanoemulsion of resveratrol, a powerful natural antioxidant with low oral bioavailability, for better management of neurodegenerative disorders, a nanoemulsion (*o*/*w*) containing vitamin E was formulated. The nanoemulsion was prepared by the self-emulsification method followed by high-pressure homogenization, and within the scope of in vitro characterization, globule size, zeta potential, refractive index, viscosity, surface morphology, and in vitro and ex vivo release studies were performed. Pharmacokinetic studies with the optimized formulation showed that the concentration of the drug in the brain was higher (brain/blood ratio: 2.86 ± 0.70) following intranasal administration. Histopathological studies also showed that degenerative changes decreased in resveratrol nanoemulsion-applied groups. GSH and SOD levels were significantly higher and MDA level was significantly lower in the resveratrol nanoemulsion-applied group [324].

In a similar study, a hyaluronic acid-based mucoadhesive lipidic nanoemulsion containing resveratrol was developed for the transnasal treatment of neurodegenerative disorders. The mucoadhesive nanoemulsion was reported to be safe on the nasal mucosa and managed to increase the amount of RSV in the brain (approximately 7-fold increase in AUC_0–7_). Hyaluronic acid-based lipidic nanoemulsion has proven itself as a successful carrier that increases the solubility, stability, and targetability of RSV in the brain [325].

Cubosomes are different nanovesicles of two continuous cubic structures with high surface area, formulated by dispersing the liquid crystal in an aqueous environment. It is expressed as a unique drug delivery system and its use in different treatment areas has increased significantly recently [326]. In one study, which aimed to deliver Resveratrol loaded on cubosomes was dispersed into the gelling polymer (poloxamer 407) to form in situ gel and deliver it to the brain transnasally. After determining the optimum formulation by in vitro characterization, ex vivo permeation and in vivo biodistribution studies were performed. Significantly higher transnasal penetration and better distribution to the brain were achieved compared to the oral route. It was concluded that cubosomal gel and resveratrol have the potential for transnasal brain targeting and use in neurodegenerative disorders [327].

Apart from resveratrol, the most remarkable compound of the stilbene class and the one that has been studied extensively, successful results have also been obtained with the nanoformulation of some other stilbenes. The chemical instability and low solubility of oxyresveratrol, a polyphenolic stilbene with a wide range of biological activities, in aqueous solutions, reduces its bioavailability and may prevent it from exhibiting neuroprotective activities. In a study, an inclusion complex was prepared with β-CD to increase the dissolution rate and oral availability of oxyresveratrol alone and together with alkoxy glycerol (AKG) obtained from shark liver oil, a compound that has attracted attention in terms of immune defense, anticancer, and anti-inflammatory activities, to reduce the level of Tau protein. According to the data obtained from the studies, dietary supplementation of oxyresveratrol-CD together with AKG improved learning and memory abilities during the climbing test in Tau flies. The study shows that oxyresveratrol-CD and AKG are effective as neuroprotective agents and that they have positive effects on the increased permeability of pharmacological agents across the blood–brain barrier (BBB), a critical barrier for the CNS, induced by AKG [328].

In another study focusing on the unclear role of oxyresveratrol-β-cyclodextrin on cognition and histone deacetylase activity in AD, in silico docking and molecular dynamics simulation analysis indicated that oxyresveratrol potentially targets histone deacetylase-2 (HDAC2) and therefore, its in vivo ameliorative effect was evaluated in rats with experimental AD induced by intracerebroventricular injection of streptozocin (STZ) (3 mg/kg). Low-dose (200 mg/kg), high-dose (400 mg/kg) oxyresveratrol-CD, and donepezil (10 mg/kg) were administered to rats for 21 days. Interestingly, oxyresveratrol with CD inclusion reversed the cognitive-behavioral deficit and significantly reduced the levels of MDA and HDAC, similar to the effect of the standard drug donepezil. The findings suggest that the efficacy of oxyresveratrol is enhanced by the CD complex, and that low-dose administration (200 mg/kg) may have more attractive effects than high-dose administration; however, the mechanisms underlying the protective effect of oxyresveratrol-CD remain to be elucidated [329].

Self-microemulsifying drug delivery system (SMEDDS) is an effective approach to improve poor water solubility and oral bioavailability, which has been recently developed specifically for oral use and is an isotropic mixture of oil, surfactant, and co-surfactant along with the drug [330]. In the study aiming to determine the neuroprotective effects in mice by analysis of memory impairment, brain tissue oxidation, and histological features, free oxyresveratrol or oxyresveratrol-SMEDDS were administered orally to mice at different doses (90, 180 or 360 mg/kg) once daily for 14 days, and a single intracerebroventricular injection of neurotoxic Aβ (25–35) peptide was administered on day 8 during oral treatment. The oxyresveratrol-SMEDDS formulation allowed a fourfold reduction in the dose of free oxyresveratrol required to prevent neurotoxicity effects caused by Aβ (25–35) peptide, and a significant decrease in behavioral disorders, lipid oxidation levels, and neuronal cell loss was observed in all hippocampal subfields (*p* < 0.0001). These results indicated that SMEDDS is a suitable formulation for the oral administration of oxyresveratrol and has the potential to increase the efficacy of the compound in the treatment of AD [331].

Liu et al. developed a nanoemulsion for higher therapeutic efficacy of pterostilbene, a stilbene compound that exhibits neuroprotective effects with its antioxidant and anti-inflammatory properties but has poor bioavailability. The results obtained from various behavioral tests, immunofluorescence analysis, Western blot, and quantitative reverse transcription polymerase chain reaction showed that pterostilbene nanoemulsion was more effective in improving learning and memory function, protecting hippocampal neurons, and preventing apoptosis and oxidative stress compared to unformulated pterostilbene. The increased efficacy was interpreted as the stronger promotion of nuclear factor erythroid 2-related factor 2 signaling pathway by pterostilbene nanoemulsion. The increased bioavailability of pterostilbene suggests that it is a potentially safe, effective, and useful treatment option for AD [332].

**Figure 18:**
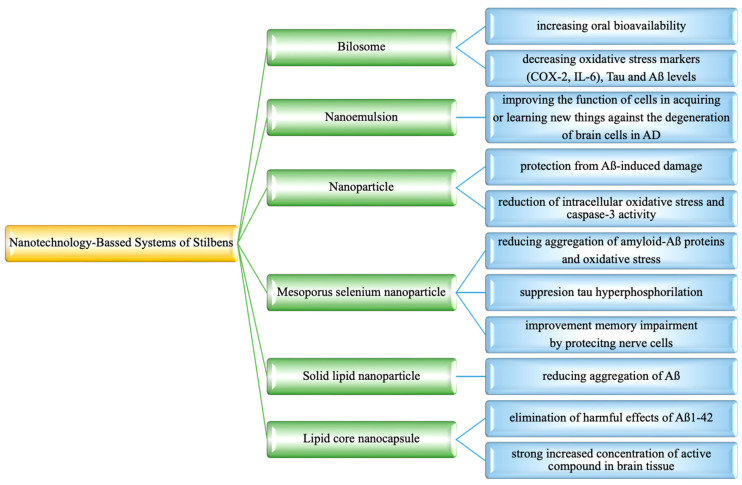
Nanotechnology-based systems containing stilbenes.

To sum up, when the results of all the mentioned studies were evaluated, it was concluded that by nanoformulating stilbenes, their low bioavailability, brain uptake, and therapeutic effect were increased, and the symptoms that caused AD decreased and/or disappeared (Figure 18). Besides the need for further studies, various nanoformulations with different routes of administration appear to help overcome the current bioavailability issue, taking into account patient compliance.

## 10. Conclusions

Alzheimer’s disease is a neurological disorder affecting especially older adults, leading to severe memory loss, confusion, social withdrawal, and changes in behavior. Key factors in its progression include Aβ buildup, NFTs and SPs that disrupt brain function as well as oxidative stress, genetics, and inflammation. Although there are FDA-approved treatments, many patients see minimal benefits. Care strategies should prioritize the enhancement of cognitive function while simultaneously aiming to slow the progression of neurodegenerative diseases. The weight of compelling evidence emphasizes the need for alternative therapeutic approaches that can address these complex health challenges. In this context, researchers are actively exploring natural compounds derived from various plants, which have demonstrated significant potential in alleviating symptoms and effectively decelerating the advancement of these debilitating conditions. The pharmaceutical research sector has justifiably spotlighted these natural compounds, particularly those sourced from the plant kingdom, due to their remarkable potential to treat various ailments with minimal adverse side effects. Among the noteworthy candidates in this area are stilbenes, a particularly intriguing class of natural phenolic compounds that warrant further exploration. Stilbenes are distinguished by their unique molecular structure, which consists of a biphenyl core interconnected by an ethylene bridge. The unique arrangement of atoms within stilbenes contributes to a rich array of biological activities, setting the stage for their promising role in pharmacological research and developing innovative therapeutic agents to combat various health issues. Stilbene derivatives exhibit a wide range of structural diversity, from individual units to octamers. They can encompass a variety of substituents, such as glycosyl, hydroxyl, methyl, or isopropyl groups positioned at different locations. The modifications of stilbenes exhibit a wide range of properties, enabling potential therapeutic activities. For instance, the positioning of hydroxyl groups within the stilbene structure is essential for determining anti-inflammatory characteristics and cellular permeability. Stilbene derivatives possess antioxidant properties and can modulate inflammation markers, suggesting potential protection against chronic diseases. Numerous studies have also demonstrated that stilbenes can have neuroprotective effects on the molecular mechanisms that cause AD. They can reduce the generation and aggregation of Aβ, a protein associated with toxic plaques, and enhance its clearance from the brain. Additionally, stilbenes help manage tau neuropathology by preventing the abnormal phosphorylation of tau proteins, further linked to Alzheimer’s progression. They also scavenge ROS, reducing oxidative stress that contributes to neuronal damage and behaving as anti-neuroinflammatory drugs. Numerous comprehensive reviews emphasize resveratrol as the most abundant and well-known natural stilbene derivative due to its extensive research and potential health benefits. However, there is a significant gap in the current literature that investigates the biological activity of other stilbene compounds, including less-studied natural variants and synthetic stilbenes. Additionally, examinations of their nanoformulations, which could enhance their effectiveness and bioavailability, remain largely overlooked. This lack of exploration limits our understanding of the full spectrum of stilbene compounds and their potential applications in various fields.

This review provides a comprehensive analysis of the advancements in treatment strategies for AD by delving into various aspects of stilbenes. It examines the intricate chemistry behind these compounds and outlines their synthesis pathways. Furthermore, this review evaluates the findings from preclinical studies on both natural and synthetic stilbenes, highlighting their potential effectiveness in combating AD. Additionally, it explores the promising benefits of employing nanoformulations of stilbenes, emphasizing how these innovative delivery methods could enhance therapeutic outcomes from a broader perspective. This review discusses both naturally obtained stilbene derivatives and semi-synthetic derivatives, as well as their potential therapeutic effects in treating AD, by compiling numerous in vitro and in vivo studies. By detailing the mechanisms through which stilbenes exert their effects, this review aims to contribute to the development of next-generation therapeutic options for Alzheimer’s and related neurodegenerative disorders. In conclusion, stilbene derivatives appear to be highly promising for treating Alzheimer’s and similar neurological diseases, as supported by scientific evidence demonstrating various and several mechanisms of action. The effects outlined, which are strongly supported by extensive research, are irrefutable and clearly demonstrate their significance. This article also delves into the fascinating nanocapsulation process, a technique that has gained significant attention in recent years. This innovative method is celebrated for its remarkable ability to improve the bioavailability of a wide range of natural compounds, including stilbenes. By encapsulating these compounds at the nanoscale, the process enhances their absorption and efficacy, opening up new possibilities for their use. This process has consistently proven to be not only effective in delivering desired results but also essential for the overall functionality and success of treatment. Its careful implementation has been a key factor in overcoming challenges and driving progress, highlighting its critical role in the treatment of neurological disorders. The insights shared in this article may shed light on the intricate relationship between stilbenes and AD. By exploring this connection, the findings could inspire a new wave of research among scientists and researchers eager to deepen their understanding of the disease and its potential treatment options.

## Figures and Tables

**Table 1:** Summary of preclinical studies of resveratrol concerning AD.

Model	Concentration/Dose	Key Findings	Reference
**In vitro** **(Aβ_42_ aggregation inhibition)**	50, 100 µM	Prevented sufuranyl free radical formation and cross β-sheet structures Decreased secondary structure content Decline in unordered nontoxic aggregates	[185]
**In vitro** **(Aβ aggregation inhibition, microplate assay, tissue-based assay)**	40.8 nM	Blocked Aβ_1-42_-HiLyte488 molecules Aβ_1-42_ aggregation reduced by 50%	[146]
**In vitro** **(BACE-1 inhibition,** **oxytosis inhibition)**	IC50: 28 µM EC50: 4.667 µM	Inhibited BACE-1 Declined oxytosis	[145]
**In vitro** **(BACE 1 inhibition)**	11.9 µM	Inhibited β-site APP-cleaving enzyme 1 (BACE-1)	[144]
**In vitro** **(*Caenorhabditis elegans* AD model)**	100 µM	Mitigated Aβ toxicity Reduced Aβ aggregation and lysosomes amount Activated proteasomal degradation	[142]
**In vitro** **(HEK293 cells)**	20–40 µM	Lowered Aβ_40_ and Aβ_42_ Activated AMPK Inhibited mTOR	[172]
		Reduced secreted Aβ levels, activated AMPK, induced autophagy	[173]
	100 µM	Inhibited MID1-α4 complex Reduced MID1 transcript and protein level Increased PP2A activity and dephosphorylates Tau at PP2A-sensitive locations	[182]
**In vitro** **(HUVEC cells)**	30 µM	Reduced endothelin-1 secretion and mRNA levels	[164]
	10 µM	Increased t-PA and u-PA antigen levels Increased expression of surface-localized fibrinolytic activity	[167]
**In vitro** **(IMR-32 cells)**	30 µM	Upregulated Bcl-2, downregulated bax, protective against Aβ toxicity	[181]
**In vitro** **(N9 cell line, LPS stimulated)**	15 and 30 μM	Inhibited MMP-9, iNOS, IL-1β, and IL-6 Overexpressed SIRT1	[161]
**In vitro** **(Neuro2a cells)**	10 μM	Resveratrol activates AMPK independently of SIRT1, promotes neurite outgrowth DecreaseD PGC-1α acetylation Increased PGC-1α activity	[159]
**In vitro** **(PC12 cells, Aβ_25–35_ induced)**	25 μM	Deceased ROI formation Inhibited PARP cleavage and JNK phosphorylation Increased expression of Bcl-X_L_	[180]
**In vitro** **(PC12 cells, Aβ induced)**	Combinations 50 μM catechin and 10 μM resveratrol or 25 μM resveratrol and 10 μM catechin	Protective against ROS toxicity and Aβ toxicity	[179]
**In vitro** **(Thioflavin-T assay** **SH-SY5Y human neuroblastoma cells, Aβ_1-42_ induced)**	Molar ratios of 1:0.01, 1:0.1, 1:1, 1:10, and 1:100 1 µM	Cleaved Aβ_1-42_ peptide Reduced toxicity of Aβ_1-42_	[15]
**In vivo** **(C57Bl/6J mice)**	200–400 mg/kg/day for 15 weeks High fat diet with resveratrol	Increased PGC-1α protein Improved mitochondrial function Effective against obesity and insulin resistance	[154]
**In vivo** **(C57BL/6J SIRT 1 knockout mice)**	25–30 mg/kg/day and 215–230 mg/kg of body weight/day with high fat diet	Activated AMPK in a SIRT1-independent manner Improved mitochondrial function	[156]
**In vivo** **(LPS stimulated mice)**	4 mg/kg i.p. 7 days	Improved estrogen level Increased NEP level	[165]
**In vivo** **(p25 transgenic mice (model of AD and tauopathies)**	5 μg/μL for 3 weeks intracerebroventricular injection	Reduced neurodegeneration Enhanced learning Lower caspase-3 levels Decreased astrogliosis in hippocampal regions Inhibited SIRT 1 substrates PGC-1alpha and p53 acetylation	[148]
**In vivo** **(PS19 mice)**	40 mg/kg for 5 weeks	Reduced tau phosphorylation, neuroinflammation, and synapse loss, Restored cognitive deficits	[183]
**In vivo** **(SAMP8 mice)**	1 g/kg for 7 months supplemented with trans-resveratrol diet	Reduced tau phosphorylation by inhibiting CDK5 and GSK3β Activated AMPK pathways and SIRT1 Increased ADAM10 expression Reduces Aβ burden	[147]
**In vivo** **(Tg19959 mice)**	1 g/kg resveratrol-supplemented diet for seven months	Inhibited tau hyperphosphorylation and kinase activities such as CDK5, GSK3β No effect on JNK	[147]
	0.2% resveratrol with diet	Reduced plaque formation and Aβ deposition No significant changes on SIRT1 activation or APP processing	[174]
**In vivo** **(Tg2576 mice)**	0.2 mg/L in Cabernet Sauvignon	Positive effect on APP processing, preventing Aβ peptide generation	[175]
**In vivo** **(Tg6799 mice)**	60 mg/kg for 60 days oral gavage	Decreased amyloid plaques Reduced Aβ_42_ and Aβ_40_ levels in the hippocampus Decreased APP, sAPPα, sAPPβ, and BACE1 (β-secretase) expression levels	[14]
**In vivo** **(Wild-type C57BL/6 mice)**	25 mg/kg i.p. for 2 weeks	Reduced tau phosphorylation dephosphorylation of Tau in cortical neurons	[182]
**Lifespan determination using PSY316AT *MAT*α** **HEK 293 cells**	100–200 µM 0.5 µM	Lowered Michaelis constant of SIRT1 Increases cell survival Stimulated SIRT1-dependent deacetylation of p53 Stimulated Sir2, Increased DNA stability	[162]

**Table 2:** Summary of preclinical studies of piceatannol concerning AD.

Model	Concentration/Dose	Key Findings	Reference
**In vitro** **(PC12 cells, Aβ_25-35_-induced)**	10, 20 µM	Inhibited internucleosomal DNA fragmentation, nucleus condensation, PARP cleavage, caspase-3 Reduced ROS formation	[187]
**In vitro** **(Neuro2a cells, Hek 293 APPsw cells)**	10–80 µM (oxyresveratrol), 2.5–20 µM (piceatannol)	Prevented sAPP secretion, decreased γ-secretase activity, increased α-secretase activity Activated MMP-9 Reduced Aβ_1–40_ and Aβ_1–42_ levels	[188]
**In vitro** **(THP-1 cells)**	6–10 µM	Increased SIRT1 protein expression in a concentration-dependent manner	[189]
**In vitro** **(PC-12 cells, H_2_O_2-_induced)**	5–20 µM	Improved mitochondrial function Increased TFAM, PGC-1α Restored SIRT3 expression	[191]
**In vitro** **(Neuro2a cells, high glucose-induced)**	5, 10 µM	Reduced mitochondrial superoxide production Stabilized mitochondrial membrane potential	[192]
**In vitro** **(PC12 cells, Aβ-induced)**	25 µM	Enhanced phosphorylation of Akt and Bad Inhibited Bcl-2/Bax expression Suppressed cleavage of caspase-9, caspase-3 and PARP	[197]
**In vitro** **(ndSH-SY5Y cells, Aβ-induced)**	1 µM	Suppressed Aβ-induced neurite fragmentation and neuronal cell death	[196]
**In vitro** **(Rat primary cortical neurons, Aβ_25-35_-induced)**	20, 50 µM	Promoted cell survival, reduced ROS levels Activated PI3K/Akt pathway Inhibited caspase-9, caspase-3, and PARP cleavage	[198]
**In vitro** **(N2a Cells, Colistin-induced)**	5, 50 µM	Suppressed ROS, activated NRF2/HO-1 pathway Protected against apoptosis	[199]
**In vitro** **(Antimycin A-induced ROS model, C2C12 cells)**	10, 30 and 50 µM	Reduced ROS, inhibited apoptosis more effectively than resveratrol Induced heme oxygenase-1 (HO1) expression Prevention from mitochondrial ROS-induced cell death via SIRT1-dependent and -independent pathways	[193]
**In vitro** **(DPPH, AChE inhibition, amyloid aggregation)**	IC50: 40.2 µM DPPH 271.74 µM AChE Inhibition 0.48 µM amyloid aggregation	Piceatannol most active in DPPH scavenging, AChE inhibition, and Aβ peptide aggregation	[186]
**In vivo** **(Rat, γ-radiation/reserpine-induced)**	10 mg/kg BW/day (oral)	Enhanced mitochondrial biogenesis via SIRT1/p38-AMPK Reduced oxidative stress, inflammation, and apoptosis	[16]
**In vivo** **(CIRI mouse model)**	10, 20 mg/kg/day (oral)	Protected hippocampus neurons Improved neurological function Reduced ROS, Bax, and caspase 3	[190]

**Table 3:** Summary of preclinical studies of oxyresveratrol concerning AD.

Model	Concentration/Dose	Key Findings	Reference
**In vitro** **(BACE1 inhibition)**	1.5 × 10^5^ M	Oxyresveratrol was a specific BACE1 inhibitor with reduced effect on other proteases	[171]
**In vitro** **(BV-2 cells, LPS-induced)**	10 µM	Reduced inflammation via MAPKs and NF-κB signaling Inhibited pro-inflammatory mediators	[190]
**In vitro** **(Cortical astrocyte and neuron cultures)**	10 µM	Lower p62 expression and p62/SQSTM1 levels Increased the expression of lysosome-associated membrane protein 1 (LAMP1) Attenuated APP production Regulating AMPK/ULK1/mTOR-dependent autophagy induction	[17]
**In vitro** **(HMC3 cells, IL-1β-induced)**	10, 20, 40 µM	Suppressed PI3K/AKT/p70S6K pathway activation Reduced IL-6 and MCP-1 release in microglial cells	[201]
**In vitro** **(Rat cortical neurons, Aβ_25-35_-induced)**	1, 10 µM	Inhibited [Ca^2+^]c increase, glutamate release, ROS production Prevented apoptosis	[202]
**In vitro** **(SH-SY5Y cells, H_2_O_2-_induced)**	5–100 µM	Neuroprotective effect by mitigating oxidative stress, Reducing ROS Preventing lipid peroxidation	[207]
**In vitro** **(SH-SY5Y cells, rotenone-induced)**	10, 20 µM	Increased cell viability, GSH levels Decreased MMP, ROS, Bax, cytochrome C, and caspase-3 activity	[204]
**In vivo** **(MCAO rat, I/R damage)**	10, 20 mg/kg (oral)	Reduced brain infarct volume Improved neurological impairments Blocked caspase-3 activation and cytochrome C release	[203]

**Table 4:** Summary of preclinical studies of pterostilbene concerning AD.

Model	Concentration/Dose	Key Findings	Reference
**In vitro** **(Neuroblastoma cells, SH-SY5Y)**	2.5, 5, or 10 μM	Reduced ROS Delayed cell death Improved mitochondrial function Increased Nrf2, HO-1, and GST levels	[208]
**In vitro** **(HT22 cell neurons, glutamate-induced)**	1, 3 or 10 μM	Reduced oxidative stress through Nrf2 activation Decreased glutamate-induced apoptosis Increased GSH levels and SOD enzymatic activity Enhanced HO-1 expression and reversed NQO1 downregulation	[209]
**In vitro** **(PC12 cells, H_2_O_2_-induced)**	10 μM	Pinosylvin most effective in reducing cell death Improving mitochondrial function Activating Nrf2/PINK1/Parkin pathway	[210]
**In vitro** **(PC12 cells, Aβ_25-35_-induced)**	25, 50 μM	Increased cell viability, reduced apoptosis and ROS Activated PI3K/Akt pathway	[197]
**In vitro** **(BV-2 cells, Aβ_1−42_-induced)**	5, 10 μM	Suppressed iNOS/NO Reduced NLRP3/caspase-1 inflammasome activation Decreased TNF-α, IL-1β, IL-6	[13]
**In vitro** **(Isolated mouse neurons, Aβ_25-35_-induced)**	2 μM	Inhibited mitochondrial apoptosis Increased neural plasticity Slowed neuronal loss via SIRT1/Nrf2-mediated mechanisms	[217]
**In vivo** **(SAMP8 mice)**	120 mg/kg diet	Restored cognitive performance Rduced Tau phosphorylation No significant effect on SIRT1 or acetylated p53	[211]
**In vivo** **(Rat, streptozotocin-induced memory loss**	10, 30, 50 mg/kg (oral)	Improved memory Increased cholinergic transmission Enhanced brain antioxidant activity (SOD, catalase, GSH) Reduced nitrite, lipid peroxides	[212]
**In vivo** **(Rat, streptozotocin-induced)**	20 mg/kg/day (5 weeks)	Reduced Aβ_1−42_ accumulation, tau hyperphosphorylation, neuronal apoptosis, inflammation Increased SOD and GSH	[213]
**In vivo** **(Mice, Aβ_1-42_-induced)**	10, 20, 40 mg/kg (oral)	Mitigated neuron loss Reduced ROS, increased antioxidant genes Promoted Nrf2 translocation	[214]
**In vivo** **(Rat, Morris water maze)**	40, 160 mg/kg diet	Enhanced cognitive performance and working memory Reversed dopamine release	[215]
**In vivo** **(Aged rat, working memory test) and two declarative memory tests**	22.5 mg/kg (oral)	Reversed aging effects on memory Increased REST, PSD-95, mitochondrial porin1, CREB phosphorylation	[216]
**In vivo** **(Male KM mice, Aβ_25-35_-induced)**	40 mg/kg (oral)	Increased expression of SIRT1 Enhanced learning–memory, working-memory, spatial learning-memory Increased NeuN, PSD-95, and SYN-1 proteins Increased Bcl2/Bax	[217]

**Table 5:** Summary of the neuroprotective mechanisms of stilbene derivatives in neurodegenerative disorders.

Compound(s)	Target Action	Specific Effects	Concentration	Reference
**2-methoxy-5-(2,3,4-trimethoxypheny) styrylpyridine**	Reduction MDA, Increasing SOD activity and gSH levels, Inhibition of NO	Decrease lipid peroxidation and nitric oxide content by inhibiting inducible NOS (iNOS) expression and activity Increase free radical scavenging activity Restore endogenous antioxidation Inhibit apoptosis through caspase-3 and caspase-9 inhibition Protect cell membrane integrity	25–100 μmol/L	[295]
**3,3′-dimethoxy-4,4′-dihydroxystilbene triazole (DMDHSB)**	Aβ-induced neuronal damage protection	Reverses up-regulation of iNOS production Prevents Aβ treatment-associated increase in NF-kB nuclear translocation	8 μM	[299]
**Amphiphilic compounds**	Targeting AβO	Disrupt interactions between AβO and cell membranes	10 mM	[294]
**Carbazole-stilbene hybrids**	ChE inhibition, Aβ aggregation inhibition, Antioxidant, Metal chelation	Potent inhibitory activity against AChE and BChE Significant inhibition of self-mediated Aβ_1-42_ aggregation	IC50: 2.64 μM (for AChE), 1.29 μM (for BChE)	[297]
**Coumarin-resveratrol-inspired hybrids**	MAO-B inhibition	Significant potency as MAO-B inhibitors, high selectivity	pIC50: 6.959 (for trans-6-Styrylcoumarin)	[282]
**Furocoumarin-stilbene hybrids**	AChE, BChE, β-secretase, COX-2, and LOX-5 inhibition, Antioxidant	Significant anticholinesterase activity Effective inhibition of β-secretase, COX-2, and LOX-5 Strong antioxidant properties	6.8 µM (DPPH)	[283]
**Hydroxyl-functionalized stilbenes, 2-arylbenzo[b]furans**	Neuroprotection against Aβ and glutamate-induced toxicity	Protect neurons from Aβ-mediated and glutamate-induced toxicity Anti-neuroinflammatory effects	50 μM	[290]
**Pinostilbene**	6-hydroxydopamine-induced neurotoxicity protection	Reduces LDH release and caspase-3 activity	1 to 10 μM	[276]
**Pterostilbene β-amino alcohol derivatives**	AChE and BChE inhibition, Antioxidant, Neuroprotective	Potent inhibitory effect on EeAChE, antioxidant properties Neuroprotective against H_2_O_2-_induced injury	IC50: 24.04 μM (5f for AChE), 40.23% (Aβ_1-42_ aggregation)	[298]
**Pterostilbene/** **Resveratrol Carbamate derivative**	Inhibition of AChE and BChE, enhancing cholinergic neurotransmission	Dual ChE inhibitor Neuroprotective	6.3 µM (AChE, Rivastigmine Tartrate), 1.37 µM (BChE, Rivastigmine Tartrate)	[278]
**Pyridoxine-resveratrol hybrids**	Dual inhibition of AChE and MAO-B	High potency in inhibiting AChE and MAO-B	IC50: 2.11 μM (7d), 1.56 μM (8b) for AChE; 2.68 μM (7e) for MAO-B	[208]
**Rhenium complexes**	Targeting Aβ plaques in cerebral amyloid angiopathy	Moderate affinity towards Aβ_1-42_ aggregates, intense labeling of Ab plaques, potential as SPECT imaging agents	Binding affinity with a Ki value of 37 Nm (complex 7), Ki value of 78 Nm (complex 14)	[301]
**Stilbene, Benzofuran neolignan derivatives**	AChE inhibition, Neuroprotectiv, Anti-inflammatory	Inhibit AChE and NO production Protect against cell damage	IC50: 39.3 ± 1.2 μM (Grossamide), 29.8 ± 0.9 μM (Boehmenan)	[292]
**Tacrine-resveratrol hybrid**	AChE inhibition, Aβ aggregation inhibitionA	Potent inhibition of hAChE Impedes Aβ self-aggregation	IC50: 0.80 μM (5 for hAChE)	[271]
**Trans-stilbene derivative**	Neuroprotection without significant interference with estrogen receptor or aryl hydrocarbon receptor signaling	Neuroprotective	EC:12 µM	[303]
